# Adapting attentional control settings in a shape-changing environment

**DOI:** 10.3758/s13414-023-02818-x

**Published:** 2024-01-02

**Authors:** Yunyun Mu, Anna Schubö, Jan Tünnermann

**Affiliations:** Department of Psychology, Cognitive Neuroscience of Perception and Action, Philipps-University Marburg, Gutenbergstraße 18, 35032 Marburg, Germany

**Keywords:** Adaptive choice visual search, Selective attention, Attentional control, Shape, Online experiment

## Abstract

In rich visual environments, humans have to adjust their attentional control settings in various ways, depending on the task. Especially if the environment changes dynamically, it remains unclear how observers adapt to these changes. In two experiments (online and lab-based versions of the same task), we investigated how observers adapt their target choices while searching for color singletons among shape distractor contexts that changed over trials. The two equally colored targets had shapes that differed from each other and matched a varying number of distractors. Participants were free to select either target. The results show that participants adjusted target choices to the shape ratio of distractors: even though the task could be finished by focusing on color only, participants showed a tendency to choose targets matching with fewer distractors in shape. The time course of this adaptation showed that the regularities in the changing environment were taken into account. A Bayesian modeling approach was used to provide a fine-grained picture of how observers adapted their behavior to the changing shape ratio with three parameters: the strength of adaptation, its delay relative to the objective distractor shape ratio, and a general bias toward specific shapes. Overall, our findings highlight that systematic changes in shape, even when it is not a target-defining feature, influence how searchers adjust their attentional control settings. Furthermore, our comparison between lab-based and online assessments with this paradigm suggests that shape is a good choice as a feature dimension in adaptive choice online experiments.

**Supplementary Information:**

The online version contains supplementary material available at 10.3758/s13414-023-02818-x.

When searching for specific objects, humans are known to extract statistical regularities from the environment, such as the distractor locations and target location, frequency to guide their attention, which is known as *statistical learning* (Failing & Theeuwes, 2018; Ferrante et al., 2018; Wang & Theeuwes, 2018a). For instance, when targets appear in the same context, in which they have been selected before, they can be selected more quickly than in new contexts, even when participants are seemingly unaware of the repeating contexts (Chun, 2000; Chun & Jiang, 1998).

Color and shape are among the features that the human visual system registers preattentively to facilitate the deployment of attention (Adamo et al., 2010a, 2010b; Theeuwes, 2013; Treisman, 1988; Treisman & Gelade, 1980; Wolfe, 2021). Color can often capture attention even if it is not relevant to the task (Theeuwes, 2004, 2018; Theeuwes et al., 2006; Theeuwes & van der Burg, 2011). Abundant research has used the color dimension to define distractors (e.g., Feldmann-Wüstefeld et al., 2015; Irons et al., 2012; Moore & Weissman, 2014) or to combine it with other features like location (e.g., Adamo, et al., 2010a, 2010b; Adamo et al., 2008), motion (e.g., Harris et al., 2015), and size (e.g., Biderman et al., 2017) to study the attentional capture effect. This might be due to the versatile discriminability of color (Theeuwes, 1992) and the flexibility of color to be combined with other features.

Furthermore, this may be partly due to perceptual priming for color occurring at an earlier stage than that for shape in visual processing (Adamo et al., 2010a, 2010b; Breitmeyer et al., 2004). Color can also trigger larger capture effects that impact attentional control settings and receive more fixations as a target-defining feature than shape (Adamo et al., 2010a, 2010b; Theeuwes, 1992), and color distractors can impair performance to a stronger degree compared with shape when they are predictable factors (Feldmann-Wüstefeld et al., 2015). Similarly, Williams (1966) showed that in a field containing objects that differed widely in size, color, and shape, observers fixated more and better on the basis of color than on shape or size.

Previous studies have used shapes or forms to investigate implicit learning and the effect of context cueing (e.g., Chun & Jiang, 1998; Goujon et al., 2015). For example, when participants searched for a *T* target among many *L* distractors, repeated (previously seen) configurations could facilitate the search compared with novel configurations (Chun & Jiang, 1998). Different orientations of shapes (Ferrante et al., 2018) or different shapes like diamonds and circles (Wang & Theeuwes, 2018a, b) can also be used to manipulate or distinguish targets and distractors.

When it comes to dynamically changing environments, it has been found that changing colors are able to guide attention in visual search tasks (Irons & Leber, 2016, 2018) and that they affect attentional control settings even if color did not define the targets (Bergmann et al., 2020). This is not surprising given the strong impact of color on attention guidance in many situations described above. However, despite the large number of previous visual search studies that used shape to define targets and distractors, it is still unclear whether dynamical changes of shapes in the environment could affect observers’ attention similarly, especially when shape is not strictly task relevant. In the present study, we investigate whether the shape dimension can be used to tune observers’ attentional control settings when it is not a target-defining feature for the task and includes dynamical changes over the course of the experiment.

## Choice as a measure of attentional control settings

The adaptive choice visual search task (ACVS), a paradigm with choice as a measure, was put forward by Irons and Leber (2016) to investigate the degree of goal-directed attentional control in dynamically varying contexts. Their search displays consisted of around 50 large and small squares, each of which appeared in one of four possible colors. One small red square and one small blue square were targets in each trial. Participants were free to choose either of two targets without any restrictions. The searching environment was manipulated by a color change of distractors. A number of distractors changed their color over trials from red to blue and back. The color similarity of distractors with one or the other target type manipulated the difficulty and the efficiency of searching for the red and for the blue target. For instance, when the variable distractors were red (red plateau), searching for the blue target would be more efficient. Since participants were informed of the changing dynamics of the environment, participants were assumed to adapt their target choices by selecting the target that shared the color with a smaller distractor subset. The results showed that, on average, observers adjusted their target choices to the color change in the environment. They tended to select the blue target among increasingly red distractors and switched to red while blue distractors increased gradually over trials. The pattern of target preference followed the dynamical color manipulation with a slight delay of about one to two trials (Irons & Leber, 2016).

## Rationale of the present study

Observers can not only learn from target-defining features in the changing environments and adapt their attentional control settings correspondingly (Irons & Leber, 2016), but also adjust to changes in a dimension that does not define the targets (Bergmann et al., 2020). Though color could lead to adaptive behavior (Bergmann et al., 2020; Irons & Leber, 2016; Zhang et al., 2023), it is unclear whether shape could also result in adaptation. We hypothesized that people would also adapt to changing regularities of shape. This adaptation might be weaker than the adaptation to color, given the strength of the influence color has in other search paradigms. Nevertheless, we expected participants to prefer the uniquely shaped target over the one with more distractors and to continuously adjust their target selection to the shape changes in the environment.

Online experiments have become increasingly popular recently. While it is difficult to control the exact color in which stimuli are presented to remote participants in front of their own displays, stimuli controlled by the shape dimension can be rendered more stably. Hence, if shape works as a feature in ACVS, it might be a good choice as a feature in online search experiments using this or other similar paradigms. It is less prone to undesired variability that can arise from how the stimuli are rendered on remote devices. To assess this possibility, we ran the current study both online (Experiment 1) and in the lab (Experiment 2). In the online context, uncertainty and diversity among online participants’ experimental environments could lead to weaker adaptation. We expected that in the lab context, where there was more control and consistency over the experimental environment, the adaptation would be stronger.

## A shape-based adaptive choice visual search task

Participants searched for one of two color singletons (items with a dark blue contour) among black-contour distractors. The targets could be distinguished and selected purely based on their color without taking further account of any other dimension. The color targets always differed in shape: star-like or pentagon-like (for simplicity, we will call these shapes star and pentagon in the following) and were embedded in a variable number of distractors sharing the shape state with either of two targets. Across trials in one experimental block, the proportion of each shape state in distractors changed dynamically and systematically.

**Fig. 1:**
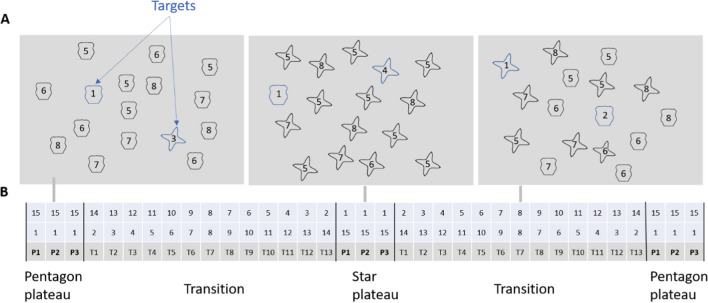
Example search displays and the trial sequence in one cycle in the search task. (**A**) The display always contained two blue targets and 14 black distractors. One target was always shaped as star and the other as pentagon. The example trial sequence started from pentagon plateau (no star distractors: **A** left display) and transitioned to star plateau (no pentagon distractors: **A** middle display) by increasing one star distractor per trial. (**B**) The numbers in the first row represent the number of pentagon items in each display, and the numbers in the second row represent the number of star items throughout the trial sequence of a block. The labels in the third row refer to the phase in a block, with “P” meaning plateau and “T” meaning transition. For instance, “P1” means the first trial of a plateau, and “T7” means the seventh trial in the transition phase. (Color figure online)

As Fig. 1 depicts, each block began with three plateau trials (P1, P2, and P3), in which all distractors had the same shape state, and only one target was unique in another shape state. In the example display, all distractors were in the shape of pentagon, while only one blue target was in the shape of star (“pentagon plateau”). In the subsequent transition trials (T1 to T13), the number of star distractors increased one by one over the trials, while the number of pentagon distractors decreased one by one until it came to another plateau, in which all distractors were shaped as stars and only one target was in the shape of pentagon (“star plateau”). A reverse transition then headed back to a pentagon plateau.

Although the task can be completed without taking the shape dimension into account by focusing on the color only, we assume that participants take advantage of the shape ratio change of the distractors. In particular, they might select the target that shares the shape state with the smaller distractor subset. By making use of the shape ratio change of distractors, the number of stimuli that has to be inspected can be reduced. As the number of distractors of a unique shape state increases gradually, the efficiency of selecting the target from this shape state decreases. If observers adapt to the shape ratio changes, they are likely to switch to another shape state target around the center of the transition. Observers adapt to the color (ratio) change of distractors while searching for shape (Bergmann et al., 2020; Irons & Leber, 2016). However, it could be the case that while searching for color, observers might not adjust to the shape changes at all or to a weaker degree, as shape might not be as prominent as color in visual search contexts. In tallies of the literature about which features guide attention, shape only ranks in the category of “probable guiding attributes,” while color is an “undoubted guiding attribute” (Williams, 1966; Wolfe & Horowitz, 2004, 2017). In addition to the degree of adaptation that might differ between color and shape, the delay with which attention is tuned could be different—again, possibly being weaker for changes in the less conspicuous shape feature.

## Experiment 1 (Online)

### Method

#### Participants

 Seventy-nine participants (32 female, 47 male) between 19 to 69 years old (*M* = 28, *SD* = 8.47) were recruited on Prolific.com (an online participant recruitment platform) to participate in the experiment for payment. All reported normal color vision and normal or corrected-to-normal visual acuity. Participants read and agreed to a consent form before taking part in the experiment. Since the individual sessions were only half as long as in typical lab-based ACVS experiments, we recruited substantially more than earlier lab-based studies that had 25 to 45 participants, to ensure that we would be able to obtain sufficiently precise parameter estimates (Bergmann et al., 2020; Lee et al., 2022; Zhang et al., 2023). The experiment was conducted in accordance with the Declaration of Helsinki and approved by the Ethics Committee of the Faculty of Psychology at Philipps-University Marburg.

#### Apparatus

 The experiment was conducted online. The participants were asked to sit in an appropriate working environment, avoid interruptions, and use laptops or desktop PCs with both mouse and keyboard. The experiment was programmed in OpenSesame (Version: 3.2.7; Mathôt et al., 2012) and served via JATOS (Lange et al., 2015).

#### Stimuli

**Fig. 2:**
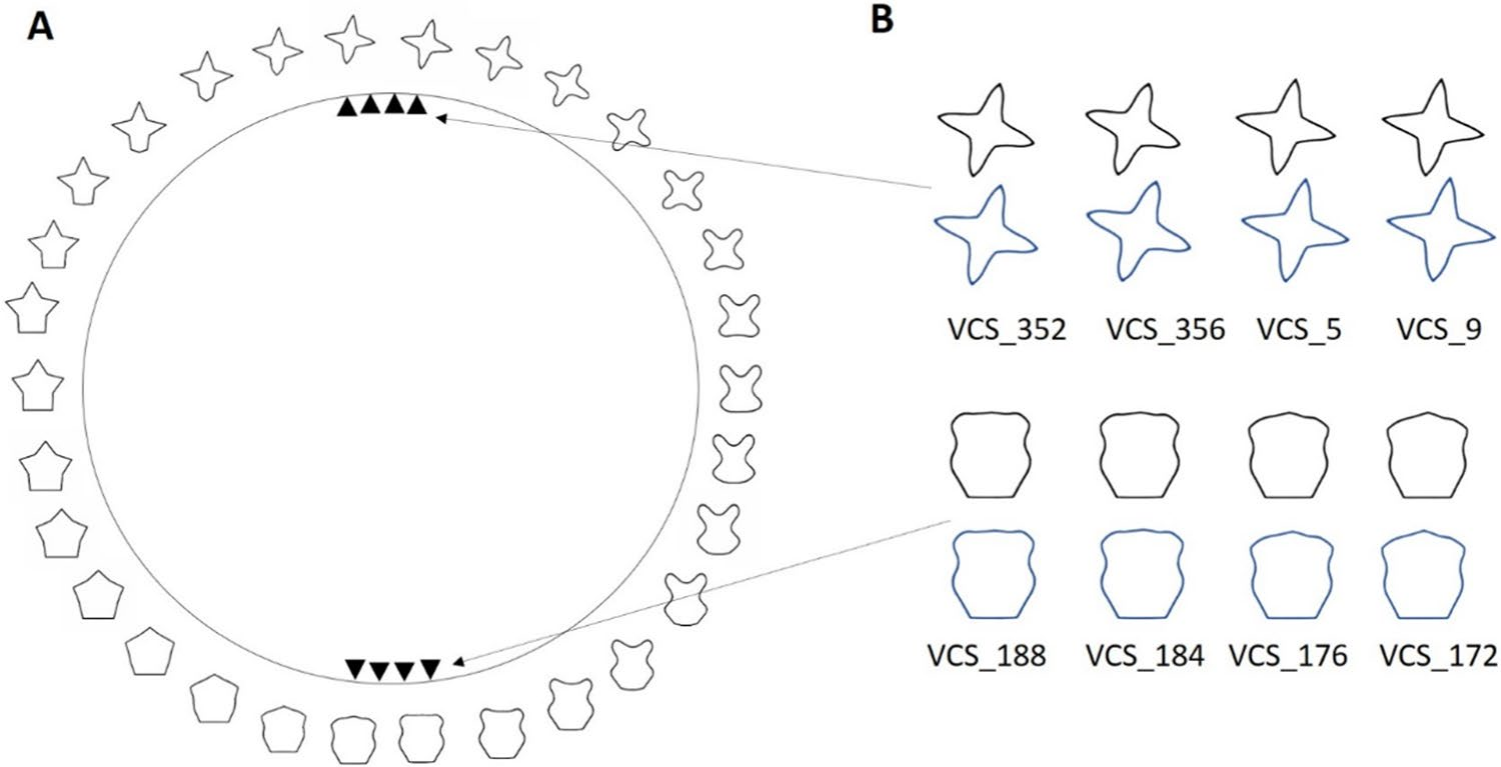
(**A**) Illustration of the VCS space with stimuli mapped to every 12 degrees of angular distance according to Li et al. (2020). The full VCS space circular contains 360 shapes. It is a validated circular shape space comparable to the color wheel, whereby angular distance along a circle is a proxy for visual similarity. The eight triangles on the circle represent the locations of eight shapes we used: four star shapes located on the north pole while four pentagons located on the south pole of the VCS space. (**B**) The eight shapes used in the current study. The digit in each position label represented the angle in the VCS space. The blue shapes (lower rows) were used as targets and the black shapes (upper rows) were used as distractors. (Color figure online)

 The visual search displays always consisted of 16 items—14 black shapes (distractors) and two blue shapes (targets) arranged on an imaginary 5 × 7 grid (Fig. 1). Each target was blue (RGB: 29, 73, 153), and each distractor was black (RGB: 0, 0, 0) on a gray background (RGB: 191, 191, 191). Due to anti-aliasing, some pixels on the contour had fainter colors. Each item was either star or pentagon shape. To achieve some variability within the shape categories, each of the two shape subsets contained four equidistant shapes taken from the validated circular shape space (VCS space; see Fig. 2) developed by Li et al. (2020). Based on the VCS space, every two adjacent shapes in each subset had the same just noticeable differences, a measure of visual similarity. The two targets were a blue star and a blue pentagon. Varying numbers of distractors shared the same shapes as the targets and were all colored in black. Each item had a black digit in the center. The two targets always contained random digits from one to four and differed in the digits they carried. The distractors were always assigned random digits between five and eight (see Fig. 1A).

#### Procedure

 The trial sequence consisted of cycles that contained “plateau” (three consecutive trials) and “transition” phases (13 consecutive trials). The sequence started with a plateau, in which all distractors shared one shape state (e.g., pentagon), and from trial to trial in the transition, the number of distractors in the other shape state (e.g., star) increased by one; the total number of distractors stayed the same. In this way, the distractor distribution approached the other shape plateau, in which all distractors had the other shape state (e.g., star; cf. Fig. 1). In pentagon plateaus, the star target was the only star-shaped item in the search display, while in star plateaus, the pentagon was the only pentagon item in the display. In sum, an experimental cycle consisted of 32 trials, starting with a plateau (three trials), then a transition of 13 trials, another opposite plateau (three trials) and a transition (13 trials) phase back toward to the original plateau. Whether cycles started with a “star plateau” or a “pentagon plateau” was alternated between blocks, and it was balanced over participants with which plateau participants started.

Participants were instructed that there were always two blue targets among black distractors in each search display, and they were free to choose either target. They were told to identify the digit inside the target they selected by pressing the associated key on the keyboard (1, 2, 3, 4). Participants were not informed of the trial sequence or the changing regularities of the shape proportion of distractors.

Each trial started with a fixation dot in the center of the display. After 495 ms, the fixation dot disappeared, and the search display was presented until participants reported a target digit. After each block, performance feedback (mean accuracy and response time) was presented. Altogether there were 466 trials: two practice blocks, each with 35 trials (one cycle and a final plateau) and four main blocks, each with 99 trials (three cycles and a final plateau).

#### Data analysis

 In the analysis, we used Bayesian estimation to obtain different parameters, quantify their uncertainties, and make comparisons. In textual reports, we describe the parameter posterior distributions by their modes and 95% highest (probability) density intervals (HDI; we report the mode followed by the HDI in square brackets). The models were implemented in PyMC (Salvatier et al., 2016) and fitted using Hamiltonian MCMC sampling (with the NUTS sampler; Hoffman & Gelman, 2014). We drew 20,000 samples after 1,000 tuning samples.

##### Response times

 The mean RT averaged across trials per participant per condition was calculated and compared via a Bayesian version of a two-sample *t* test (BEST procedure; Kruschke, 2013). Modes and the 95% HDIs of the RT differences between plateaus and transitions were reported.

##### Target choices 

**Fig. 3:**
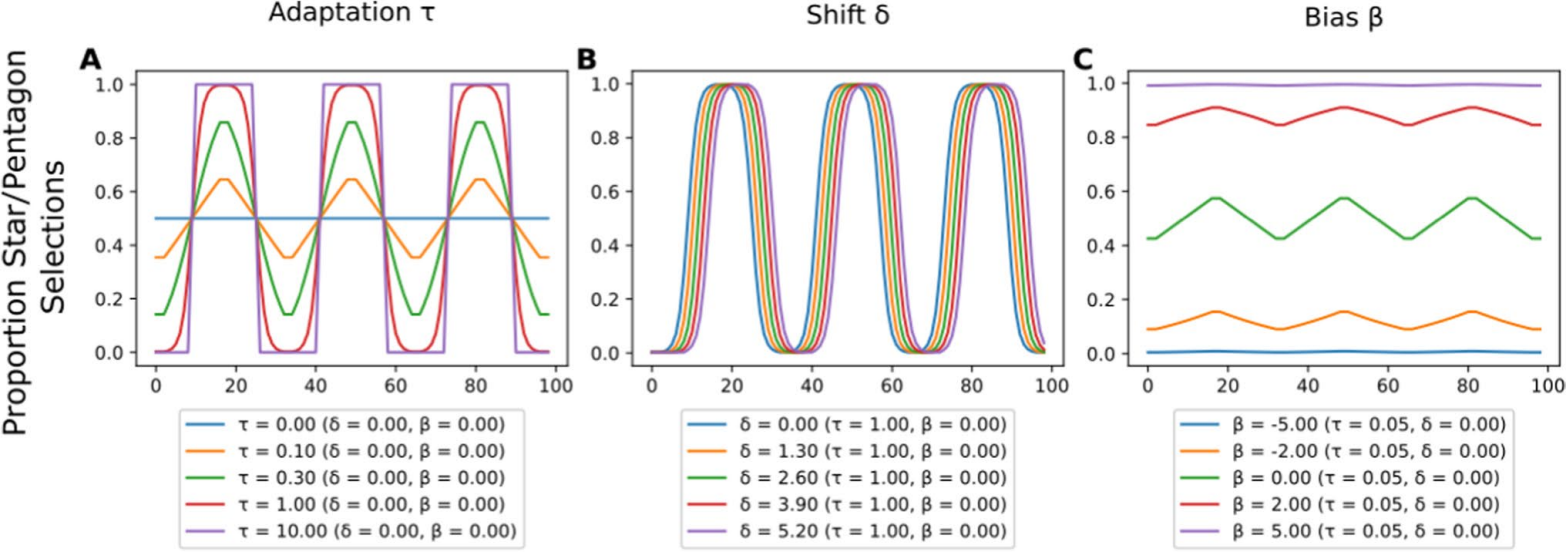
Visualizations of the adaptation curve with exemplary parameters. In each panel, the value of one parameter is varied while the others are held constant. (**A**) Different values for adaptation τ. (**B**) Different values for shift δ. (**C**) Different values for bias β. The curves depict the predicted proportion of selecting the star or pentagon target that shares the shape with a larger distractor set in a plateau, and the number of this distractor set declines at the start of the cycle. Figure style adapted from Tünnermann (2022), CC BY 4.0. (Color figure online)

**Fig. 4:**
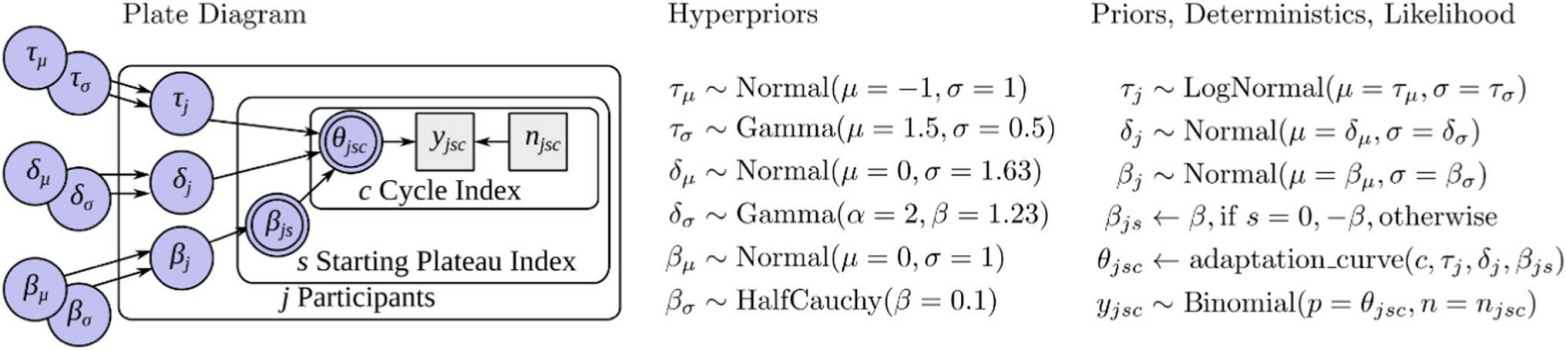
Hierarchical model structure that connects the parameters of interest at each level with (prior) distributions listed on the right. Refer to Supplement Section A2.1B concerning the *adaptation_curve* function. The plate diagram on the left follows the conventions from Lee & Wagenmakers (2014)

A model with three parameters was used to fit the target choice data. It was implemented using the ACVSfit framework (Tünnermann, 2022), and the model as well as the analysis are extensively described in Supplement A. The model is based on connected sigmoid functions that describe the rising and falling transitions and the plateaus. A formal description is provided in Supplement Section A2.1. The meanings of the parameters are explained in the following and are visualized in Fig. 3. Parameter “adaptation τ” models how much observers adapt to the objective shape ratio (see Fig. 3A). A τ of zero means no adaptation (i.e., random choice), and a τ rises toward positive infinity. The adaptation approaches that of a perfect discriminator that always selects the target in the small set. In practice, a τ of one roughly corresponds to an adaptation curve fully reaching the plateaus over the course of the transition, and a value of 10 represents already extremely strong adaptation that requires perfect discrimination to select the smaller set. Parameter “shift δ” describes the horizontal offset of the adaptation curve in trials (see Fig. 3B). The shift indicates the delay with which observers update their attentional control settings relative to the objective shape proportion. The “bias β” parameter quantifies the preference toward a particular shape state by shifting the adaptation curve vertically (see Fig. 3C). The equation for this function can be found in Supplement Section A2.1B. The adaptation function was embedded in a hierarchical Bayesian model to obtain group-level and participant-level estimates of adaptation τ, shift δ, and bias β. The model structure and priors are depicted in Fig. 4, and more details can be found in Supplement A2.1B. The priors were chosen to be sufficiently vague to let the data govern the results, and their selection is extensively discussed in Supplement A2.1C. Details on the software version, sampling process, and convergence diagnostics are provided in Supplement Section A2.2.

### Results

Blocks with an average accuracy of less than 75% were removed from all analysis (18.04% of all trials). This effectively excluded 14 participants whose accuracy was about chance level, indicating they did not follow the instructions. This criterion also excluded one more block from the other participant but the remaining three blocks of this observer remained in the analysis. From the remaining trials, incorrect trials in which participants reported a number not contained in the display (2.6%), and trials with response times longer than 5,000 ms (another 0.1%) were removed from analysis.

#### Target choices

**Fig. 5:**
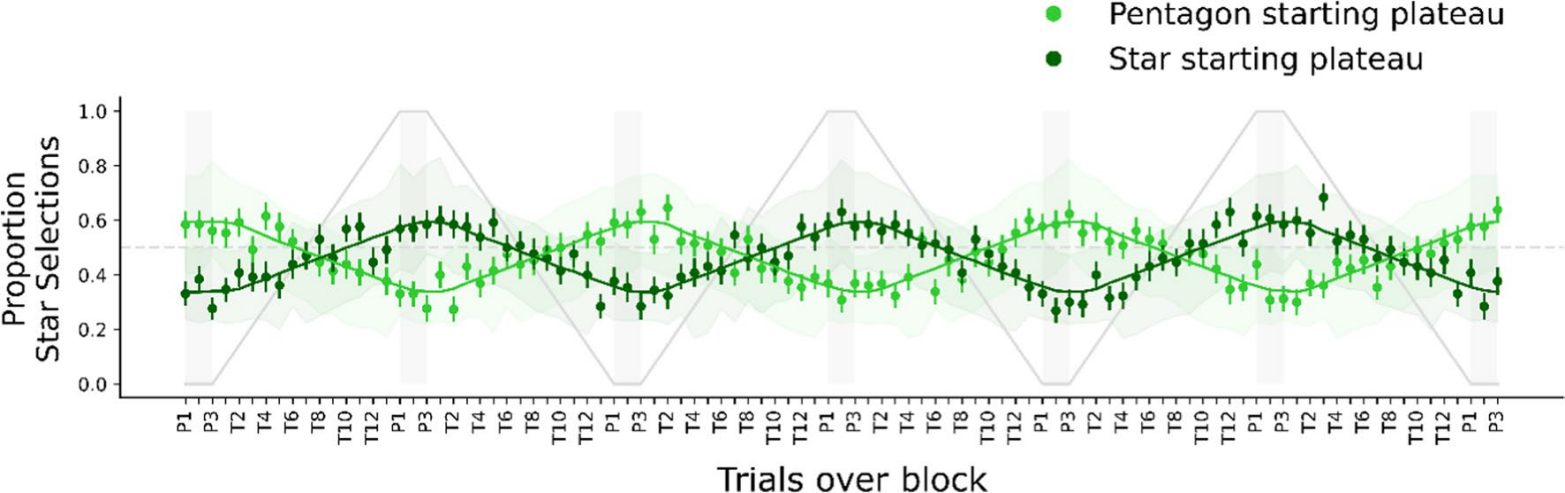
Star selections starting with two plateaus in Experiment 1 (lime green for “pentagon starting plateau” and dark green for “star starting plateau”). Observed data proportion is depicted as points, averaged across participants. Error bars on the points indicate the standard errors of the means. The solid green lines represent the mean model predictions of star selections and error bands represent the 95% highest density intervals of the mean predictions at each trial. The solid gray line illustrates the objective proportion of pentagon distractors from the star starting plateau over the block. The dashed horizontal line at *y* = 0.5 shows selection at the chance level. Light-gray rectangles highlight the areas that belong to plateaus. Figure style adapted from Tünnermann (2022), CC BY 4.0. (Color figure online)

 Target choices showed that participants adapted their selection to the dynamical changes in the environment over the trials (Fig. 5). On the plateaus, participants had the tendency to select the target that was in a unique shape state. The probability of selecting the pentagon target was slightly larger than that of selecting the star target over trials, indicating a slight, distractor-independent bias toward the pentagon targets. Overall, participants preferred the target that was from a smaller shape subset in the search display, and this tendency was stronger closer to the plateaus and weaker toward the center of the transitions, where it was possibly more difficult for observers to estimate the shape proportion.

**Fig. 6:**
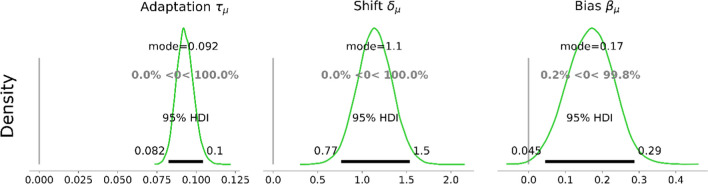
Group-level posterior distributions of adaptation τ, shift δ, and bias β estimates in Experiment 1. The horizontal black bars and the numbers on their ends indicate the boundaries of the 95% highest density interval (HDI). The gray percentages compared with zero represent the proportions of posterior distributions smaller (left) and larger (right) than zero

The estimate of adaptation τ = 0.092 [0.082, 0.1], which was shifted away from zero, further confirmed that participants adapted to the objective shape distractor proportion (Fig. 6, left panel). A τ of this magnitude reflects a degree of adaptation with which the average adaptation curves reach about 59% or 66% selections of the unique target in the plateau phases (depending on the starting plateau; cf. Fig. 5). The shift of δ = 1.1 [0.77, 1.5] in trials showed that there was a delay with which the adaptation followed the objective shape proportion in the search display (Fig. 6, middle panel). The bias β was estimated at 0.17 [0.045, 0.29] (Fig. 6, right panel). This bias β estimate indicates that participants had a slight bias toward pentagon shapes, independent of the shape proportion in the display.

In addition to the analysis over all blocks reported above we also analyzed the first and last block separately and compared the estimated parameters. This revealed that there was an increase in adaptation by 0.021 [−0.0042, 0.05], shift 0.75 trials [−0.35, 1.9]. The bias did not change by much (−0.031 [−0.23, 0.16]). Further details of this analysis can be found in Supplement B.

#### Response times

 To assess the search difficulty on plateaus and transitions, we compared the mean RTs of these parts of the cycle. The shortest mean RT was observed for plateaus (978 ms [952 to 1,007]), and responses were slower during the transitions (1,032 ms [1,007 to 1,064]). The RT difference between plateaus and transitions was 58 ms [16, 93] with zero, “no difference,” far outside the HDI.

### Discussion

The results of Experiment 1 showed that participants tuned their attentional control settings toward the changing shape ratio of distractors even when shape was not a target-defining feature, and even when the experiment was conducted online. Participants selected the star target more frequently when it was the unique shape in pentagon plateaus and the pentagon target when it was unique in star plateaus, and these preferences gradually changed over the course of the transition phases. The shift (parameter δ) estimates revealed that there was on average a delay of 1.1 trials for observers to adjust their target choice with respect to the changing environment. This shows that observers pick up the regularity of the dynamically changing environment and tune their attentional control settings accordingly but with some delay. If observers would merely take the current display into account, no shift would be expected. We also observed a slight bias β toward the pentagon shape. Even though the bounding boxes of the two shapes were of similar size, the pentagon shape was the more compact one, whose inner area was larger than the star shape, which might have rendered it more salient. However, there were also substantial individual differences in the bias parameter including two extreme cases that virtually always selected the pentagon shape (see Supplement Figs. S8A and S10A).

The RT results showed that the search difficulty increased as the trial went from plateaus to the center of the transition. This is consistent with the interpretation that the effective size of the distractor set which impairs search efficiency can be minimized by selecting the “more unique” targets toward the plateaus.

Taken together, the results of Experiment 1 are in line with earlier findings that showed that observers could adapt their behavior to the dynamical changes even if the changing feature is not necessary for solving the task (Bergmann et al., 2020). Importantly, the experiment confirmed that attentional control is adaptive in dynamically shape-changing environments as well. Moreover, the results highlight that registering the regularities does not require the variable environment features to be on the color dimension, nor does it require a highly controlled lab-based setup to tease out such effects.

## Experiment 2 (Lab-based)

The results of Experiment 1 suggested that participants adapted their behavior to the dynamical changes within the not-target-defining dimension in an online context. In Experiment 2, we conducted the same visual search task under highly controlled lab conditions. In addition, eye-tracking data were recorded to provide additional information about which items participants fixated during the search. This can help to shed light on the target choices which are not adaptive (see Fig. 5: Despite the adaptation, there is a large discrepancy between the objective shape ratio and the choices). The majority of fixations in a previous adaptive choice task were found to target the chosen target’s features rather than that of the nonchosen one (Irons & Leber, 2016), which we expect to be the case as well within the shape dimension.

We also increased the number of blocks. While we aimed to keep the online experiment short because remote participants might lose interest more quickly, this limitation was no longer present for the lab version. We expected that in the lab, with more control over the experimental environment, participants would show stronger adjustment of target choices to the changing shape ratio.

### Method

#### Participants

 Twenty-six participants (17 female, nine male) 18 to 31 years old (*M* = 22.88, *SD* = 3.05), naïve to the experiment design, participated in the experiment for course credit or payment. All had normal color vision and normal or corrected-to-normal visual acuity. Participants signed the written understanding and consent form before the experiment. The sample size is in the ballpark of similar studies that successfully demonstrated adaptive choice. The experiment was implemented in accordance with the Declaration of Helsinki and approved by the Ethics Committee of the Faculty of Psychology at Philipps-University Marburg.

#### Apparatus

 Participants completed the experiment in a sound-attenuated and dimly lit room. They were seated in a chair, placed their head on a chin rest, and were positioned 100 cm from an LCD-IPS screen (Cambridge Research System, 1,920 × 1,080 pixels, Display++LCD Monitor 32, 120 Hz). They responded to the target by pressing one of four keys on a main keyboard with the index or middle fingers of both hands (Dell Wired Keyboard, KB216t). The experiment was conducted via OpenSesame (Version: 3.2.7; Mathôt et al., 2012). An EyeLink 1000 Plus desktop-mounted eye tracker was used in the visual search task (SR Research Ltd., sampling rate 1000 Hz, spatial resolution 0.01°).

#### Stimuli and general procedure

 The shapes were identical to those in Experiment 1. The visual search displays always consisted of 16 items—14 black shapes (distractors) and two blue shapes (targets) arranged on an imaginary 5 × 7 grid (Fig. 1). Each target was blue (RGB: 29, 73, 153; 29.89 cd/m^2^) and each distractor was black (RGB: 0, 0, 0; 0.252 cd/m^2^) on a gray background (RGB: 191, 191, 191; 78.56 cd/m^2^). The resulting chromaticity was also measured with a colorimeter (see Supplement C). The star items had a maximal diameter of 1.15°, and all pentagon items had a maximal diameter of 1.38°. The experimental procedure was identical to that in the online experiment, except that an eye tracker was used for fixation control before each search display was presented. Each trial started with a central fixation mark. The fixation mark was only replaced with the search display once the participants fixated the mark. The search display was shown until participants reported the digit within the stimulus they selected by pressing a key on a PC keyboard that only included the four response keys. After each block, performance feedback for the current block was presented (mean accuracy and response time). Altogether there were 862 trials (two practice blocks with 35 trials each and 8 main blocks with 99 trials each).

#### Data analysis

 For target choices and response times, the same analyses were conducted as for Experiment 1.

#### Fixation data

 Each fixation the eye tracker detected (detected with the EyeLink online parser) was assigned to the closest stimulus in the display. Based on these fixation assignments, we calculated the proportions of first fixations, last fixations, and total fixations to the two shape types in a trial. The proportions were normalized by dividing them by the number of items with the same shape (see Irons & Leber, 2016). This step was performed because there were always more items that matched the less unique shape, and thus the chance for a random saccade to land on one of these items was higher than for targets (e.g., if selecting items by chance, participants would have been much less likely to saccade to a single star target than to land on one of the 15 pentagons in a pentagon plateau).

Based on Irons & Leber’s (2016) fixation data analysis, we also analyzed the fixation data restricted to plateaus where the numbers of two shape types were significantly different. We calculated the proportion of fixations assigned to the items matching the shape of the target participants eventually selected (chosen shape target) and to the items matching the shape of the target participants did not select (nonchosen shape target). This would show whether participants restrict their search to items matching the chosen shape and always fixate the chosen shape target first even when that target was from a larger shape subset.

### Results

All blocks had an average accuracy larger than 75% for each participant. Incorrect trials (reporting a number not contained in the display in 2.13% of trials) and trials with response times longer than 5,000 ms (another 0.02%) were removed from the analysis.

#### Target choices

**Fig. 7:**
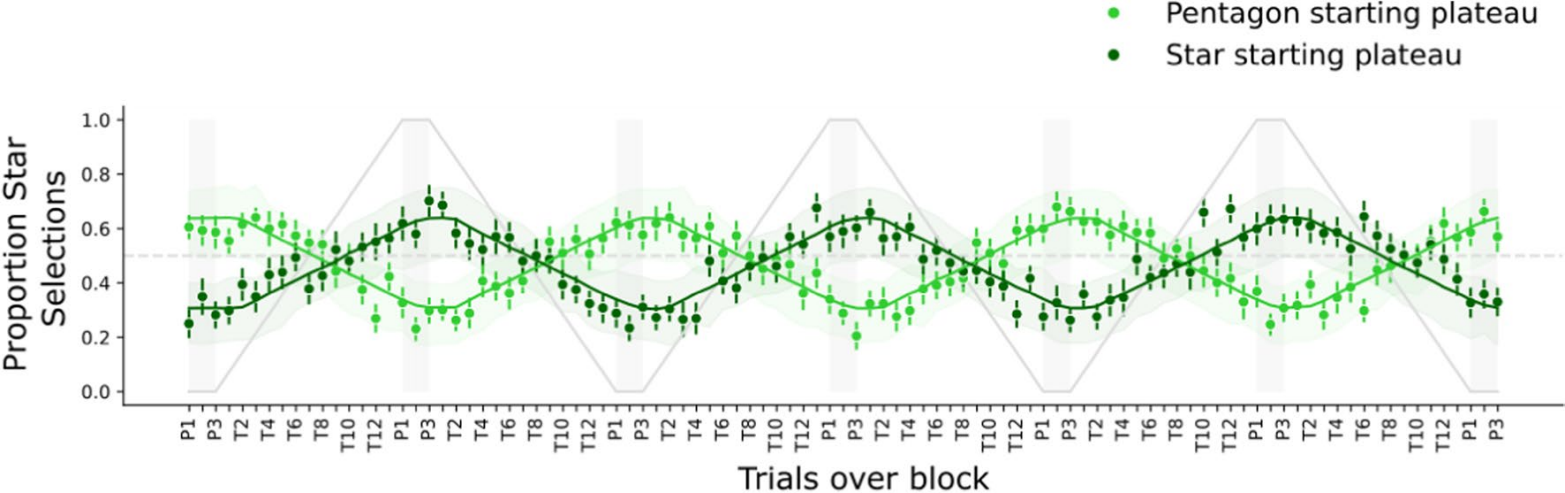
Star selections starting with two plateaus in Experiment 2 (lime green for “pentagon starting plateau” and dark green for “star starting plateau”). Observed data proportion was depicted as points, averaged across all participants. Error bars on the points show the standard errors of the means. Two solid green lines show the model prediction of star selections, and error bands represent the 95% confidence interval of the means. The solid gray line gives an example of objective proportion of pentagon distractors from the star staring plateau over the block. The dashed horizontal line at *y* = 0.5 shows selection at a random chance level. Figure style adapted from Tünnermann (2022), CC BY 4.0. (Color figure online)

 Overall, the results of target choices in Experiment 2 revealed the occurrence of adaptation to shape ratio changes although shape was not necessary to solve the task over the trial sequence (Fig. 7). On the plateaus, participants showed the tendency to select the target whose shape was from a smaller subset. Participants preferred to select the target that was more unique in the display, and this tendency became stronger close to the plateau and became weaker as approaching to the center of the transition where estimating the shape proportion became more difficult for participants.

**Fig. 8:**
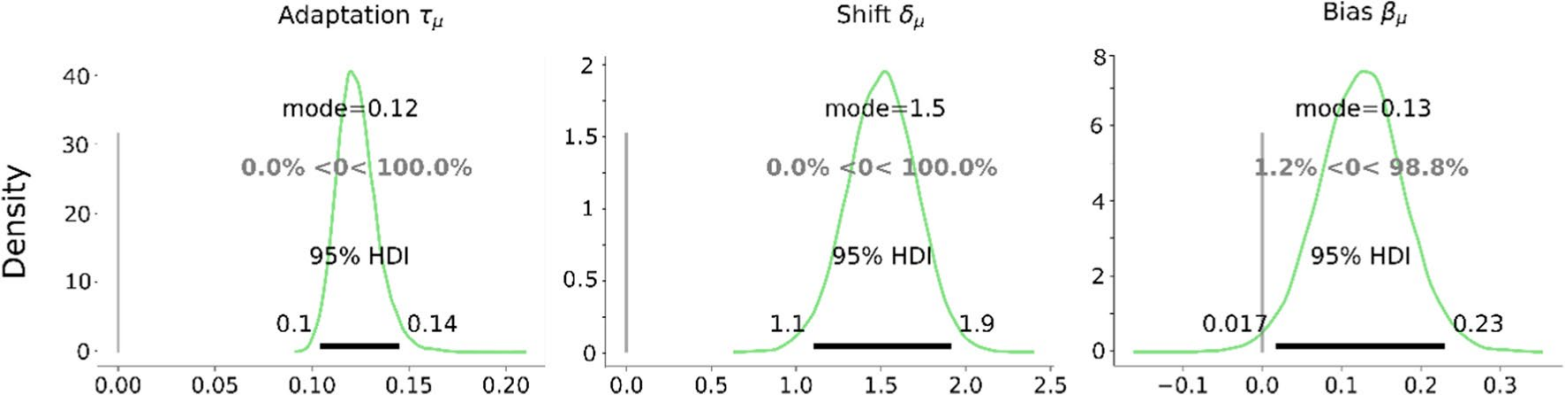
Group-level posterior distributions of adaptation τ, shift δ, and bias β estimates in Experiment 2. The black bar and numbers on its ends indicate the boundaries of 95% highest probability density. The gray percentages compared with zero represent the proportions of posterior distributions smaller (left) and larger (right) than zero

The estimated adaptation τ and shift δ reflected that participants adapted to the shape proportion change of distractors in the search task (Fig. 8). Adaptation τ was estimated at 0.12 [0.1, 0.14], and corresponded to the adaptation curves that favored the unique element in about 64% or 69% of the target reports on plateaus (depending on the starting plateau; cf. Fig. 7). shift δ was estimated at 1.5 [1.1, 1.9] trials, and bias β estimated at 0.13 [0.017, 0.23]. Again, we also analyzed the first and last block separately and compared the estimated parameters. The results showed that there was an increase in adaptation by 0.016 [−0.039, 0.072]. The shift changed only by −0.33 trials [−1.9, 1.1], similar to the bias (−0.038 [−0.22, 0.15]). Further details of this analysis can be found in Supplement B.

#### Response times

 As in Experiment 1 (online), we compared RTs between plateaus and transitions in the lab-based experiment. The shortest RTs were observed on the plateaus where one target’s shape was unique (893 ms [864 to 924]). Responses were slower during the transitions where there was no unique shape target (963 ms [936 to 997]). The RT difference between plateaus and transitions was 73 ms [32, 112] with zero, “no difference,” far outside the HDI interval. Consistent with RT results from Experiment 1 (online) the RTs on the plateaus were smaller than that in the transition phase, supporting that the search was more difficult in the transition phase.

The estimated mean of RTs averaged across the trials in the lab-based experiment was shorter (944 ms [907 to 985]) than that in the online experiment (1,027 ms [987 to 1,070]), with a difference of 86 ms [27 to 139].

#### Fixation data

 To analyze the fixation data, we excluded trials with incorrect responses as well as trials, in which the response time after the stimulus onset was faster than 100 ms (2.11% of trials were removed).

**Fig. 9:**
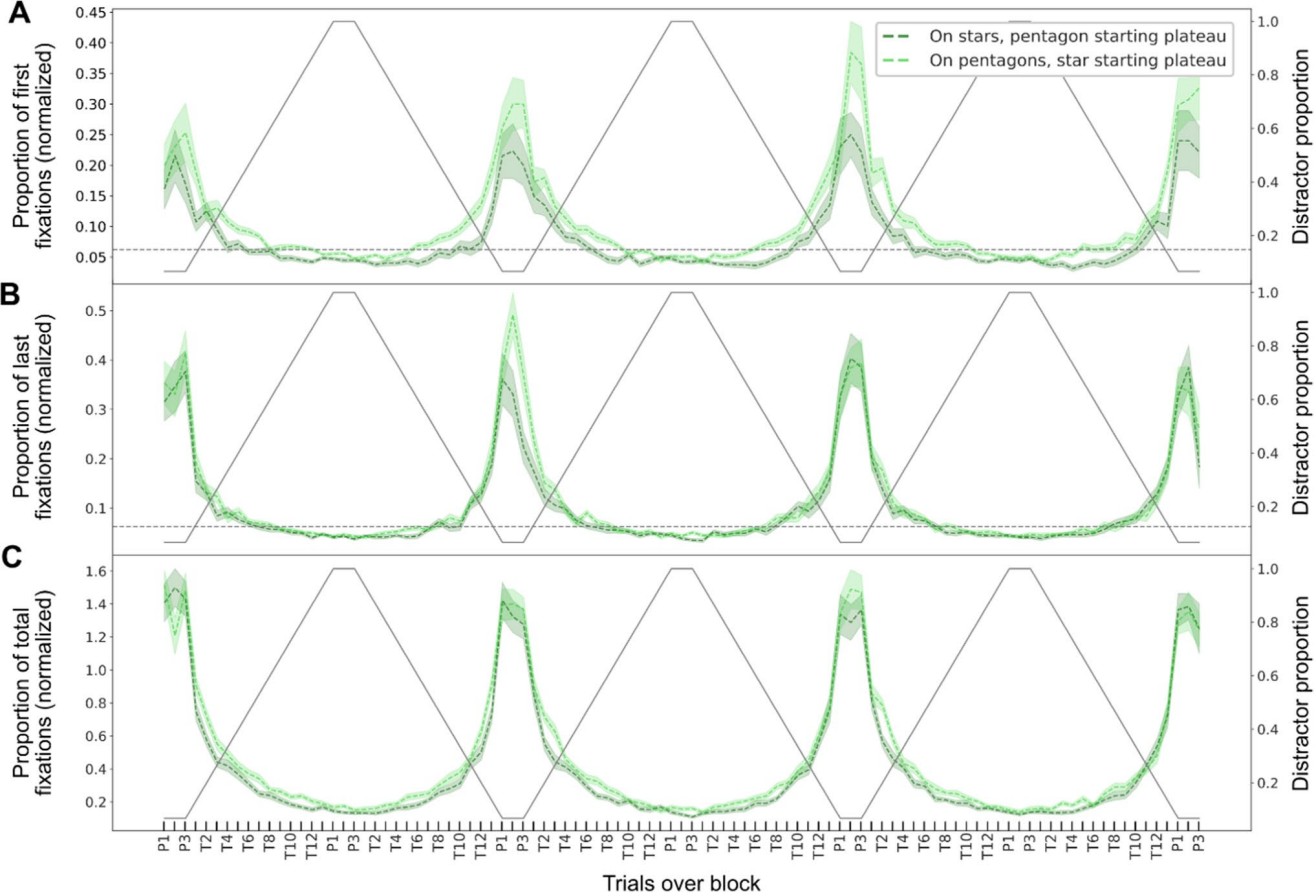
Fixation data from Experiment 2. Normalized proportions of first (**A)**, last (**B**), and the total number of fixations (**C**) averaged across trials in one block on pentagons (stars) for cycles starting with star (pentagon) plateaus. The shaded area represents the standard errors of the means. The gray dotted horizontal line (1/16) represents the chance of fixating a random item. The solid gray line represents the proportion of the two distractor shapes

The fixation data is depicted in Fig. 9 based on shape type. Generally, the proportion of fixations that landed on the more unique shape type was much higher than the chance level (the chance of randomly fixating one item among all 16 items: .0625) on the plateaus and decreased gradually as it got closer to the center of the transition (T7; see Fig. 9) where each shape type had the same number of items. The proportions of first and last fixations on the more unique shape type from the start decreased gradually as it got closer to another plateau where the other shape type was more unique, indicating that observers were more likely to fixate the more unique shape type first (Fig. 9A–B). The normalized total fixations on a particular shape varied in the same pattern (Fig. 9C).

**Fig. 10:**
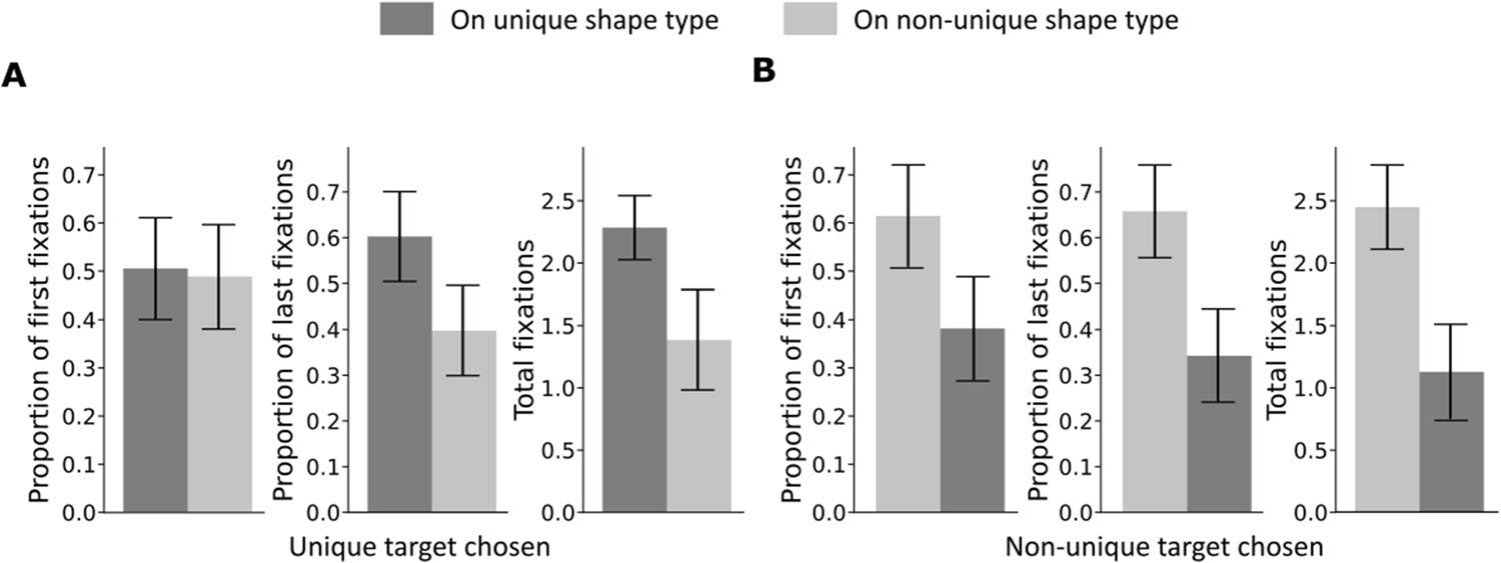
Proportions of first fixations, last fixations, and total fixations in Experiment 2 within plateau trials to unique target shape (dark bars) and non-unique target shape (light bars) (**A**) when the unique target was chosen and (**B**) when the non-unique target was chosen. The error bars represent the standard error of the mean

To investigate whether participants were more likely to fixate the shapes that matched the target they eventually selected, we analyzed the proportion of fixations on the two shape types. Table 1 and Table 2 list the absolute fixation counts (first, last, and total) for the unique versus the non-unique shape types on plateaus and the difference between these values. Table 1 contains the values for cases in which the unique target was eventually chosen and Table 2 the cases in which the non-unique target was chosen. The differences of first, last and total fixations on the unique shape type and on the non-unique shape type (see third rows in both tables) point in the direction that the eventually chosen target's shape was fixated more often, even if the non-unique target was chosen (Fig. 10).

**Table 1:** Proportions of first, last, and total fixations on two shape types on the plateaus when the unique shape target was selected

	Unique target chosen		
	First fixations	Last fixations	Total fixations
Unique shape type	.50 [.47, .54]	.6 [.58, .63]	2.3 [2.2, 2.4]
Non-unique type	.49 [.46, .52]	.39 [.37, .42]	1.4 [1.3, 1.5]
Differences	.017 [-.026, .06]	.21 [.17, .25]	0.91 [0.78, 1]

*Note.* The last row shows the difference in fixations between unique and non-unique shape types. The square brackets present the 95% highest density intervals

**Table 2:** Proportions of first, last and total fixations on two shape types on the plateaus when the non-unique shape target was selected

	Non-unique target chosen		
	First fixations	Last fixations	Total fixations
Non-unique shape type	.62 [.59, .65]	.66 [.63, .69]	2.4 [2.3, 2.5]
Unique type	.38 [.35, .41]	.34 [.31, .37]	1.1 [1,1.2]
Differences	.24 [.2, .28]	.32 [.28, .36]	1.3 [1.2, 1.5]

*Note.* The last row shows the difference in fixations between unique and non-unique shape types. The square brackets present the 95% highest density intervals

This data suggests that at some point (often early in a trial) observers might fixate on an arbitrary shape type (perhaps of an item close to the previous fixation) and then stick with this shape type until they find the target to avoid the effort of switching to another shape type (Irons & Leber, 2016).

### Discussion

The results of Experiment 2 again showed the adaptation of target choice to the varying distractor shape ratio, which was slightly delayed. The slight bias toward pentagon shapes was also replicated.

#### Comparison of Experiment 1 and Experiment 2

**Fig. 11:**
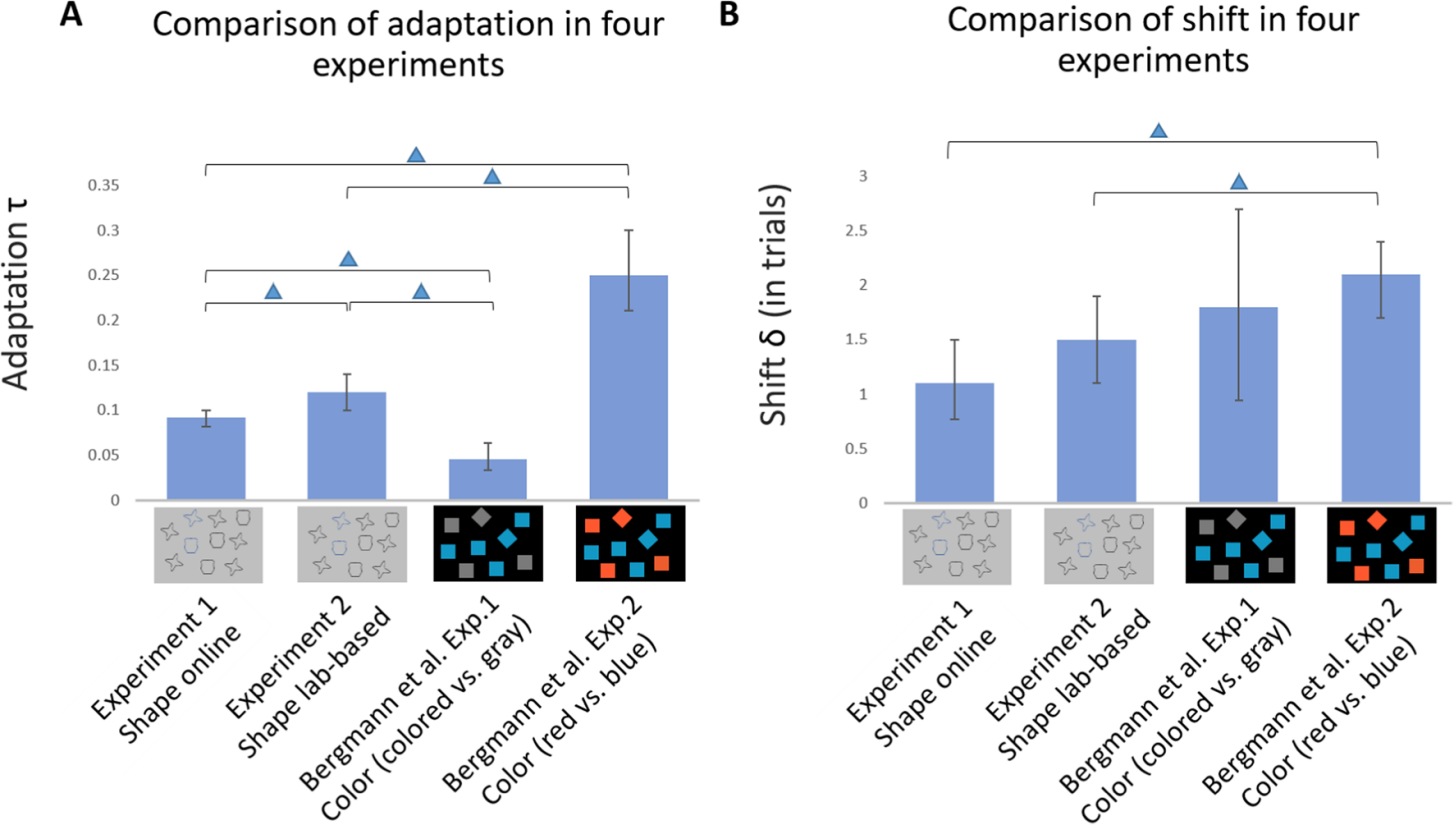
(**A**)Comparison of adaptation τ between Experiment 1 (shape online), Experiment 2 (shape lab based), Bergmann et al.’s (2020) Experiment 1 (color; colored vs. gray), and Bergmann et al.’s Experiment 2 (color; red vs. blue). The bars show the parameter posterior modes with 95% HDI error bars. The displays under the label on the *x*-axis are simplified displays of the color- and shape-based experiments for illustration. (**B**) Comparison of shift δ in trials between the four experiments. Error bars indicate the 95% HDIs. The brackets with blue triangles refer to differences whose 95% HDIs exclude zero (“no difference”). (Color figure online)

The results of the lab-based experiment showed that adaptation τ was stronger than in the online experiment with a difference of 0.030 [0.007, 0.054] (calculated as a difference of the parameter posteriors of the two experiments; see Fig. 11A). Sitting in a dimly lit and sound-attenuated room in the lab seemed to have facilitated observers’ adaptive behavior to the systematically changing regularities of shape in the environment. Participants could also have engaged in the task more seriously in the lab: there were fewer errors in the lab (2.11%) than in the online experiment (2.6%, not including trials already removed from low accuracy blocks), and in contrast to the online version, no participants or blocks had to be excluded on the basis of the 75% accuracy criterion.

The shift δ estimate was larger in the lab-based context, compared with that in the online context with a difference of 0.363 trials [−0.177, 0.923] (Fig. 11B). However, the HDI includes zero, so there remains uncertainty about this difference. The shift is likely to be larger than in the online experiment, but with an upper HDI boundary of 0.923, it is clearly below one trial. A slightly larger shift could be related to the stronger adaptation: When observers are in a mode in which they incorporate the distractor ratio more strongly into their attentional control (as shown by the higher adaptation τ), they might also be more sensitive to incorporate their selection history, leading to the lag.

The difference of the bias β estimates was small with −0.043 [−0.198, 0.124]. The RT results in Experiment 2 (lab-based) were consistent with that in Experiment 1 (online), again indicating that search difficulty generally increased as the trial went from plateaus to the transition.

#### Comparison with Bergmann et al.’s (2020) adaptation results

 In the present study, we assessed adaptive choice visual search in shape-varying contexts with a novel model. As it is difficult to judge the magnitude of our parameter estimates without a reference, we compared them with estimates obtained from that of other published experiments. Since we used the same basic paradigm as Bergmann et al. (2020), it was possible to reanalyze their data with the new model. The parameter estimated for adaptation τ and shift δ from their experiments and the experiments of the present study are depicted in Fig. 11 (note that we do not compare the bias estimates because biases toward one of two colors are not related to biases toward one of two shapes).

The adaptation τ estimate was larger in our Experiment 1 (online), compared with Bergmann et al.’s (2020) Experiment 1 with a difference of 0.046 [0.026, 0.063], but weaker than that in their Experiment 2 with a difference of −0.161 [−0.209, −0.115]. The adaptation τ estimate in our Experiment 2 (lab-based) was larger than that in Bergmann et al.’s Experiment 1, with a difference of 0.076 [0.049, 0.102], but weaker than that in their Experiment 2, with a difference of −0.128 [−0.182, −0.081]. For the shift δ estimate in our Experiment 1, it was similar but slightly smaller compared with their Experiment 1 with a difference of −0.669 trials [−1.608, 0.290] and smaller than that in their Experiment 2 with a difference of −0.910 [−1.431, −0.424]. The shift δ estimate from our Experiment 2 was similar compared with that in Bergmann et al.’s Experiment 1; the difference was estimated at −0.344, but the HDI [−1.283, 0.612] was rather large, not ruling out the difference near zero or opposite to differences. However, our Experiment 2’s shift δ was clearly smaller than that in Bergmann et al.’s Experiment 2 with a difference of −0.511 [−1.067, −0.030].

In sum, adaptation in shape-based experiments was in between the values estimated for two color experiments from Bergman et al.’s study, while the shift was slightly (less than one trial) smaller than the values estimated in their experiments. Even though the experiments from Bergmann et al. (2020) and the present study were very similar in design (e.g., same number of items in each display), many details (e.g., exact colors and shapes) seem to be important in this regard. Therefore, we could not conclude that the shape context was stronger or weaker, which was also not our aim to compare them. However, the comparison provides a rough picture about how large the adaptation and shift effects found in the present study are compared with earlier work in general.

## General discussion

In the current study, we ran two experiments to investigate attentional adaptation to a dynamically changing environment, which was realized through the distractor shape ratio change in a visual search task. Participants were free to choose either of two color targets among black distractors. Moreover, we reanalyzed the data of two similar published experiments that used color instead of shape. Since in previous studies shape was found to trigger weaker attentional capture effects than color (Adamo et al., 2010a, b; Feldmann-Wüstefeld et al., 2015; Theeuwes, 1992), and in the current search task, shape was not necessary for participants to find the target, we hypothesized that people might adapt to shape changes, but perhaps not very strongly. Observers would adapt to the changing shape ratio by preferring the color target from a smaller shape subset and would change their preferences accordingly during the transition trials as the proportion of shape inverted.

The results indicated that participants adjusted their target selections to the dynamically changing distractor shape ratio. On plateaus, they preferred to select the target that was unique in shape state and gradually switched to another shape target when the shape ratio inverted. This adaptive behavior existed in both the online experiment and the lab-based experiment. Estimated shifts revealed that there was a small delay of 1 to 1.5 trials in updating the target choices relative to the objective shape ratio of distractors throughout the trials in each experiment. The adaptation to the changing regularities and delays in updating target preferences were consistent with the results of Bergmann et al.’s (2020) color-based findings, though they differed in the degree. Adaptation in shape-based experiments was in between the values estimated for Bergmann et al.’s experiment with a “colored (blue) versus gray” manipulation and their experiment with a “red versus blue” manipulation. The gray and bluish colors in Bergmann et al.’s experiment (which also included substantial heterogeneity by using variable hues for the blue) might have provided a relatively low salience or feature contrast for observers to establish distractor subsets to guide their selection strategy. Hence, it provides an example at the low end of the range of feature contexts that enable adapting to changing color ratios. The red and blue in Bergmann et al.’s second experiment provide a much stronger color contrast (and the hues in a display were not heterogeneous), possibly providing an example at the high end of adapting to changing color ratios. Hence, the shape features we used in the present study seem to produce feature contexts which reside in the middle region of such a scale.

The results of Experiment 1 indicated that the shape dimension in a visual search task worked well in the online context, which could hint that the shape dimension is a good choice for the online experimental context. The difference between the results of the online and the lab-based experiments will be picked up at the end of this discussion.

### Adapting to shape while searching for color

As a version of the adaptive choice visual search task, our experimental design differed in an important way. In previous adaptive choice studies (Clarke et al., 2022; Irons & Leber, 2016, 2018), the target was defined by their size and color. The changing regularities of color could guide the attention and affect the target choices. In our paradigm, color was the only target-defining feature while shape was not necessary to find the target. The changing regularities in the environment were manipulated via the shape rather than the color. Color often shows stronger cueing effects in attentional capture than shape (Biderman et al., 2017) and thus it was often used as a dominant feature to study attention (Feldmann-Wüstefeld et al., 2015; Harris et al., 2015; Irons et al., 2012). The color dimension also provides a great deal of versatility in defining the distractors and the targets for attentional capture studies (Adamo et al., 2008, 2010a, b) and seems to have been the go-to feature in attention studies. In the present experiments, even though the changes occurred in the shape dimension, participants adapted to the dynamical changes, preferring the target from a smaller shape subset. Moreover, the adaptation manifested despite the fact that shape was unnecessary for the task. Hence adaptation to features of the background, even if they do not define the targets, seems not to be limited to color. This could hint at a more general mechanism that monitors the environment for statistical regularities and employs them in attention guidance.

To explain the observed behavior, we assumed that observers adapted their attentional control settings, taking the distractor context in the shape dimension into account. This brings up the question of how these attentional control settings are implemented and modulated over the course of the experiment. One possibility is that observers form target templates (cf. Liesefeld et al., 2023), and adjust them over time. In principle, such a target template could include any information that helps to identify the target. In the present case, there could be templates that include the target color, the possible shapes, and even the digits within them. However, the digits are not very reliable, as in any given trial, only two out of four possibilities are realized, and they are so small in size (smaller than 1°) that identifying them would require foveal fixation (cf. Shelchkova & Poletti, 2020; Stewart et al., 2020). Hence, they are not useful for guiding attention and are unlikely to be part of the template. Moreover, it has been shown that templates typically only include the information essential to guide attention, and for multidimensional targets, they only include the most diagnostic feature (Yu et al., 2023). In our experiment, color is the most diagnostic feature. Guiding attention toward blue items would always lead to a target.

The adaptation to the changing shape context we found shows that participants did include the shape feature dimension in their attentional control settings. A reason for this could be that shape seems immensely helpful in the plateaus of the cycles (see Fig. 1). There, one target was not only a quasi-singleton in color but also a singleton in shape, standing out strongly from its environment. This salience could have increased the general importance of shape. According to the dimensional weighting account, attentional weights for feature dimensions can be up- or down-weighted (Liesefeld & Müller, 2019; Krummenacher & Müller, 2012). Up-weighting shape because of its importance in the plateaus could have persisted over the whole cycle, leading to shape information being included in the target template. This explanation is also consistent with previous data from adaptive choice visual search studies (Bergmann et al., 2020).

With regard to the question of how the use of the shape information is adapted when the feature context changes over time, there are at least two possibilities: One is that the concrete shape of the items of the smaller subset is encoded in the target template which is updated (with some delay) when going through the cycle. A second possibility is that only the color information is encoded in the target template, and the shape information is encoded in a negative template (see Arita et al., 2012; Conci et al., 2019; Liesefeld et al., 2023; Zhang et al., 2022), suppressing the items of the larger subset (again updated with some delay).

Similar to the question about how the shape information is used, one can ask *when* it might be used in the search process. Especially since color information is known to strongly guide the deployment of attention (Adamo et al., 2010a, b; Theeuwes, 1992; Williams, 1966), it seems possible that observers prioritize color first and only include the shape information later in the search process. If this was the case, first saccades might land on the colored targets with a constant likelihood independent of the distractor schedule. We plotted the proportion of first saccades landing on the (blue) targets over the distractor cycle (see Supplement D) and found that this was not the case. Instead, already the likelihood of the first saccade landing on a target is modulated by the cycle. Consequently, shape information affects early attention guidance, consistent with the idea that it is a permanent part of the template carried over subsequent trials (and updated with respect to the changing environment).

That observers register changes in shape (similar as color) even when it is not a target-defining feature is interesting, because shape is often considered a more complex feature than color (Huang, 2020; Wolfe, 2021). Shapes consist of configurations of lower level features such as line segments and edges. Representing it in a three-dimensional feature space (like a color space) requires going to rather abstract dimension. For instance, in Huang’s (2020) approach, certain shapes can be represented in the dimensions “segmentability,” “compactness,” and “spikiness.” Our study added evidence to distractor interference emerging from shape features even though it is not a basic low-level feature like color. The changing ratio of shape states might have been extracted as a dynamical statistical regularity throughout the trials. This hints that including dynamical context regularities in attention guidance might be a general property of the visual system, not limited to the salient color dimension.

Though people adapted to the dynamically changing shape ratio of distractors, there was still a large proportion of target selections whose shape was from the larger subset. Similarly, inefficient choice was also found in earlier experiments (Bergmann et al., 2020; Irons & Leber, 2016). Irons and Leber (2016) suggested that this might be because participants were motivated to minimize cognitive effort. Our eye-tracking analysis results are consistent with this interpretation, showing that observers were more likely to fixate on the eventually chosen target shape first and before they made a response (last fixation). This was true both when the unique target and the non-unique target were selected (see Fig. 10). Hence, the shape state on which the first fixation lands, likely at a nearby random element, seems to explain many of the ultimate selections that went to the non-unique target. The total fixations also indicated that participants usually spent most fixations on searching through the set from which they ultimately report the target. Hence, at least sometimes, they might have stayed within the initially fixated shape set to avoid the effort associated with switching to the other set.

Interestingly, on some plateau trials, participants fixated on the target with the unique shape first but ended up reporting the other target (compare Table 2 and Fig. 10B). This could happen for different reasons. One is that fixation does not necessarily indicate processing at attentional levels as evidenced by the change blindness and inattentional blindness literature, where observers miss substantial changes or events despite fixating their locations (e.g., Mack & Rock, 1998; Simons & Rensink, 2005; Wolfe, 2021). Another reason could be that observers scan both bluish items before reporting one (the later fixated) of the targets. According to the instruction, they can choose and report whichever target they prefer and do not have to report the first detected target. Especially if they base their search template primarily on the blue color or if they are sensitive for the increased color salience of the targets, they might scan both of them before responding. Since there are only two blue items, scanning both of them would still be quick in compliance with the instruction to respond quickly.

As discussed by Bergmann et al. (2020), adaptation in the ACVS paradigm could arise in different ways, and not merely by selecting the target from the smaller subset to optimize search time. The drive of organisms to extract environmental regularities and employ them in diverse ways presents a challenge for approaches such as ideal-observers models. For example, in a split-half line segment task by Nowakowska et al. (2017), participants had to search through compound arrays that consisted of line segments, where one side had similar orientations (homogeneous) and the other side had highly variable orientations (heterogeneous). Some observers saccaded into and searched the more homogenous side in target-absent trials, even though this side could be excluded using only peripheral vision. In such trials, searching the more heterogeneous side would have been the more optimal strategy, which several observers consistently did not use.

In general, there seems to be much individual variability in how searchers apply strategies that may seem straightforward from theoretical considerations (Clarke et al., 2022; Irons & Leber, 2016, 2018; Nowakowska et al., 2019). Consistent with this, our experiments showed stronger adaptation for some individuals compared with others. A reason for the variability in adaptation could be that searchers employ other search strategies to varying degrees, or that they are influenced by low-level factors to different degrees. Concerning strategies, participants might prioritize blue over black elements initially during the search. The fixation data results showed that normalized first fixations to the blue items were much higher than to the black items (see Fig. S17 in Supplement D). That is, if observers employed a search template by sticking to color, they could find the target consistently well over the trial course. This would hence attenuate the adaptation to the shape context change. Concerning the influence of low-level factors, salience that arises from the spatial distribution of stimuli (e.g., a solitary item in an area with sparse distractors) or the facilitation of features selected on previous trials (intertrial priming, see, e.g., Wolfe et al., 2003) work against adaptive selections as well. How exactly these and other factors interact with each other and with adaptive choice behavior remains an open question for future research.

Another interesting aspect was that observers showed a bias toward the pentagon-like shapes, consistently in both experiments. Despite the fact that the shape stimuli in the current study were selected from the VCS space (Li et al., 2020), which strictly controls the visual similarity, low-level features such as the oriented line-segments that make up a shape might have influenced participants’ selective behavior, leading to the pentagon bias. Shape is a more complex higher level feature compared with color that has so far been used in ACVS experiments. The perception of shape is influenced by attribute such as, for example, segmentability and compactness (Huang, 2020; Wolfe, 2021; Wolfe & Bennett, 1997). Therefore, properties of the low-level features that make up a shape (e.g., orientations of the line segments) or shape attributes such as compactness or symmetry might interact in determining selection priority. In our particular case, the prominent horizontal line segment of the pentagon-like shapes and perhaps their relative compactness could have led to the observed bias.

### Adaptation delay

The estimated shift parameters showed a delay in updating target selection to the changing environment. In the online shape experiment, the shift in updating was smaller than that in the controlled laboratory environment. In the former, the impact of the feature context might have been somewhat reduced since observers are less shielded from other environmental influences than in a controlled lab setting. Consequently, they might strive to actively ignore any influences that are not related to the search for the targets in the actual display. This might make them less sensitive to any regularity between trials and instead base their choice more on the feature configuration in the actual trial display.

The delays were smaller when participants adapted to shape in our experiments compared with the adaptation to color in earlier experiments. This could mean that observers are less influenced by their prior experiences when updating attentional control settings in the shape contexts. This could be due to the low working memory capacity for shape contours like the patterns we used in the current study (Salmela et al., 2010). Basic visual features like color and orientation can be stored in visual working memory with high precision and little loss of information up to about four items (Luck, & Vogel, 1997). However, the storage capacity for even more than two shapes was found to be poor (Sakai, 2005; Salmela et al., 2010). Low working memory capacity for the shape contours might make it more difficult for observers to carry the information from previous trials. This could mean that in the shape contexts, attention control might have to rely more on the current displays.

### Consistency between online experiment and lab-based experiment

Online experiments have decades of history in psychological research (Reips, 2002) and have gained importance as an alternative to lab-based experiments during the COVID-19 pandemic. The online method is considered to have several advantages such as the possibility of recruiting demographically and culturally diverse participants, collecting large samples quickly and the ease of achieving high statistical power (Birnbaum, 2004; Dandurand et al., 2008). However, it also has disadvantages such as more variance concerning the experimental environment due to the network connection, equipment, lighting, and others (Dandurand et al., 2008; Reips, 2002). Several studies have shown that online experiment results were consistent with lab-based experiments results despite some subtle differences. Previous web-based experiments include logical-mathematical tasks (Dandurand et al., 2008), behavioral studies (Sweeney et al., 2014), psychophysical tasks (Krüger et al., 2021) and linguistic researches (Keuleers et al., 2015).

While participants in both the lab-based and online experiments of the present study showed adaptive behavior, the lab-based experiment had larger adaptation values and also faster RTs. Increased preference for selecting the target from a smaller shape subset could have benefited visual search performance (i.e., faster RTs), but other factors such as a more controlled environment in the laboratory could have contributed as well.

## Conclusion

In general, the current study has shown that observers adapted to a dynamical shape change in the environment while searching for color, even though the shape was not a target-defining dimension. The results show that participants extracted statistical regularities from the dynamically changing environment and utilized the information to guide their attention. Our model results show that the shape dimension can guide observers’ attentional control settings even when it is not a target-defining feature, to a degree within the range of what can be observed in color-based experiments. These findings add evidence to the account that humans perform statistical learning of changing regularities to support attentional guidance. Furthermore, the shape dimension works well with the online experimental context and maintains consistency between online and traditional lab-based experiments.

### Supplementary Information

Below is the link to the electronic supplementary material.

Supplementary file1 (PDF 5 MB)

## Data Availability

Data and analysis scripts are available at: https://osf.io/b3yxs/ and https://github.com/YunyunMu/AdaptingToShape.
