# Supplementary material for: Adapting attentional control settings in a shape-changing environment
Source: Atten Percept Psychophys. 2024 Jan 2;86(2):404–21. doi: 10.3758/s13414-023-02818-x (PMC10805924; doi:10.3758/s13414-023-02818-x)
Supplement: Supplementary file 1 — Supplementary file1 (PDF 5 MB) [file 13414_2023_2818_MOESM1_ESM.pdf]

Supplement A: Bayesian Analysis of Choice Data for:

Adapting attentional control settings in a shape-changing environment

Yunyun Mu, Anna Schubö, Jan Tünnermann

This document describes the Bayesian analysis of the choice data. It can be found as an (interactive) jupyter notebook at https://github.com/YunyunMu/AdaptingToShape.

The analysis is conducted using the acvsfit library (Tünnermann, 2022), which is a frontend for the probabilistic programming libraries PyMC (Salvatier, 2016) and ArviZ (Kumar, 2019). Other crucial libraries are numpy (Harris et al., 2020), seaborn (Waskom, 2021), matplotlib (Hunter, 2007) and pandas (McKinney, 2010). The versions are specified in Section 2.6.

The report of the analysis follows the steps of the BARG (Bayesian analysis reporting guidelines; Kurschke, 2021).

Note that it is assumed that the reader of this analysis report is familar with the rationale of the experiments, their design, and the terminology described in the main text.

1 Prerequisites

To (re)run the code in this analysis report, the following preparatory steps are required:

1.1. Setup folders

Make sure the following files and folders are on the same level in your filesystem:

- MuEtAl-2022-ChoiceAnalysis.ipynb (this file)
- data (fodler with the input files)
- phases (folder with the phases configuration files)
- figures (folder with images used in this notebook)
- traces (may contain preexisting trace files; can also be empty)
- main_text_figures (empty folder)
- summaries (empty folder)

The contents of these folders can be found here: https://osf.io/b3yxs/

1.2. Import the following python libraries & set the random seed

Please refer to Section 2.6 concerning how to install the required libraries in the correct versions.

In [2]:
```
from IPython.display import Image, display
from matplotlib.pylab import plt
import arviz as az
import acvsfit
import numpy as np
import pandas as pd
random_seed=1234
np.random.seed(random_seed)
```

1.3. Load the data sets

The lines below load the data sets of Experiment 1 and 2 of the present study as well as data from two experiments from Bergmann et al. (2020). From the latter, the "free choice" conditions are loaded.

In [3]:

```
df1 = acvsfit.load_data('data/MuEtAl-2022-Exp1-Online.acvs.csv')
mean1 = df1.groupby(['Participant_ID', 'Block'])['Correctness'].agg(['mean']).reset_index()
df1 = df1.merge(mean1,on=['Participant_ID', 'Block'])
data_exp1 = df1[df1['mean'] >= 0.75]
data_exp1 = data_exp1[data_exp1['Correctness']==1].reset_index(drop=True)

df2 = acvsfit.load_data('data/MuEtAl-2022-Exp2-Lab.acvs.csv')
mean2 = df2.groupby(['Participant_ID', 'Block'])['Correctness'].agg(['mean']).reset_index()
df2 = df2.merge(mean2,on=['Participant_ID', 'Block'])
data_exp2 = df2[df2['mean'] >= 0.75]
data_exp2 =  data_exp2[data_exp2['Correctness']==1].reset_index(drop=True)

data_bergmann_exp1 = acvsfit.load_data('data/BergmannEtAl-2020-Exp1-FreeChoice.acvs.csv')
data_bergmann_exp2 = acvsfit.load_data('data/BergmannEtAl-2020-Exp2-FreeChoice.acvs.csv')
```

1.3. Define the experimental cycles

The cell below reads files that contain specifications of the cycle in the different experiments. The present study contains cycles of length 99, during which the distractor set sizes go back and forth four times between the two plateaus. The cycles from the experiments by Bergmann et al. (2020) only contain one wave (from starting plateau to the opposite plateau and back to the starting plateau); see visualizations below. In all experiments, the plateaus consist of three trials and transitions of thirteen. Please refer to the main text and main text Figure 1, for information about how the displays changes over the cycle.

In [3]:

```
# Phases in both experiments of the present study:
phases = acvsfit.load_phases('phases/MuEtAll-2022-phases.json')

# Phases for Bergmann et al's (2020) experiments:
phases_bergmann_exp1 = acvsfit.load_phases('phases/BergmannEtAll-2020-Exp1-phases.json')
phases_bergmann_exp2 = acvsfit.load_phases('phases/BergmannEtAll-2020-Exp2-phases.json')
```

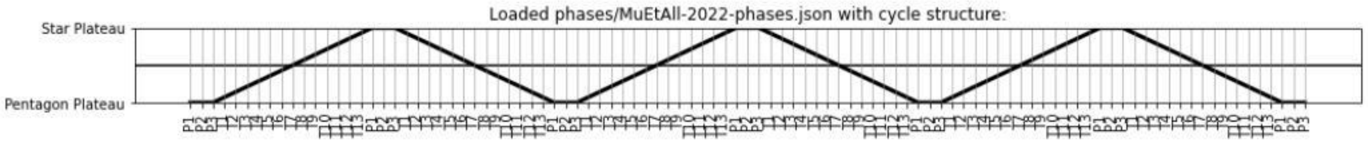

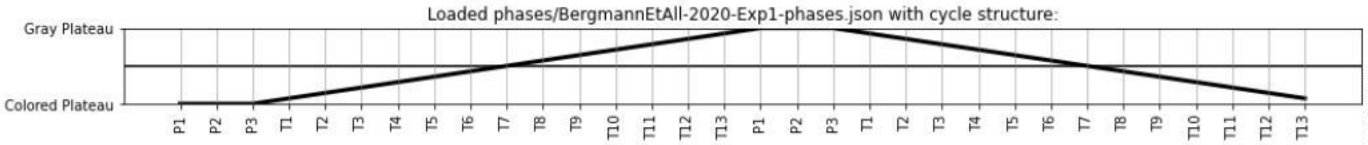

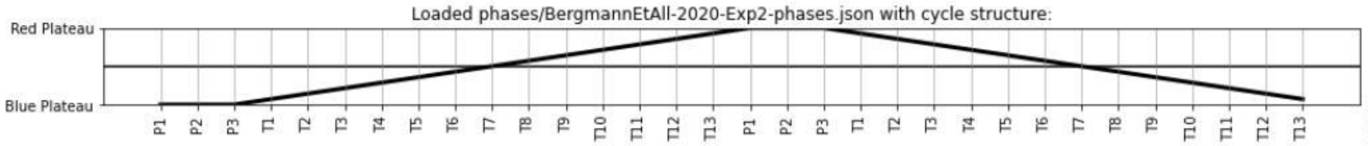

1.4. Specify plotting colors and parameters

The color dictionary below maps condition names (found in the input files) to a primary and secondary color which are used in the visualizations. The call of `az.rcParams.update` makes sure that plots and tables have the mode as point estimates and 95%-HDI to summarize distributions.

In [4]:
```
colors = {
    'Online':{0:'limegreen', 1: 'darkgreen'},
    'Lab': {0:'limegreen', 1: 'darkgreen'},
    'Bergmann Colored vs. Gray': {0: (0.36, 0.61, 0.84), 1: (0.65, 0.65, 0.65)},
    'Bergmann Blue vs. Red': {0: (0.36, 0.61, 0.84), 1: (0.93, 0.49, 0.19)}
}
az.rcParams.update({
    "plot.point_estimate": "mode",
    "stats.hdi_prob": 0.95
})
```

2 Analysis Report

The following sections contain a reproducible analysis report. The report follows the steps suggested in the BARG (Bayesian analysis reporting guidelines; Kurschke, 2021).

Preamble

A. Why a Bayesian analysis? The hierarchical Bayesian approach allows modeling the data with the appropriate data distributions (here, binomial "*k* out of *n*" responses at the different cycle levels). It allows for the estimation of parameters and their credible intervals to determine plausible values at the group and participant levels.

B. Goals of analysis. The goal of the analysis is to estimate three parameters (and their uncertainty) that meaningfully describe the adaptive choice behavior. The roles of the feature context (shape vs. color) and data recording settings (online vs. lab) are assessed by looking at the differences between estimates from the two experiments of this study and two experiments from the literature.

2.1 Explanation of the model (BARG Step 1)

A. Data variables. The dependent (predicted) variable of the analysis is the target choice. In particular, it is the count of how often one of the two targets (e.g., "Star") is selected in the repetitions of each cycle state. Within each dataset, the independent (predictor) variable is the cycle index. Over a cycle, the distractor ratios fluctuate, as described in Figure 1 of the main text.

B. Likelihood function and parameters. The model uses binomial likelihoods at each cycle index that model how often out of all repetitions of the cycle index one particular stimulus (e.g., "Star") was selected. An adaptation curve models the success rate parameter p of the binomial distributions change over the cycle index, determining which target type's selection is more likely at each cycle index. The adaptation curve (an example is shown in Figure S1) can be seen as a piecewise linear function with slopes that connect low and high plateaus. The function can be stretched and compressed in y direction and shifted in y as well as x direction. It is then passed through an inverse logit function, which maps the real numbered output into a probability range and produces the S-shaped slopes. From another perspective, the adaptation curve can be understood as a series of upward and downward bending sigmoid (logistic) functions. Formally, it is specified as

$$\text{adaptation\_curve*}(c,\tau,\delta,\beta) = \begin{cases} \left(\beta + \tau \cdot \frac{-S}{2}\right), & \text{if } c-\delta \text{ within a low plateau,} \\ \left(\beta + \tau \cdot \left(\text{cpos}(c) \cdot \frac{S}{T_{\text{len}+1}}\right)\right), & \text{if } c-\delta \text{ within a low to high transition,} \\ \left(\beta + \tau \cdot \frac{S}{2}\right), & \text{if } c-\delta \text{ within a high plateau,} \\ \left(\beta + \tau \cdot \left(\text{cpos}(c) \cdot \frac{-S}{T_{\text{len}+1}}\right)\right), & \text{if } c-\delta \text{ within a high to low transition.} \end{cases} \tag{1}$$

$$\text{adaptation\_curve}(c,\tau,\delta,\beta) = \text{invlogit}(\text{adaptation\_curve*}(c,\tau,\delta,\beta)) \tag{2}$$

where c is the cycle index. Constant S is a scaling constant (set to $S = 12$) that has been chosen so that an adaptation of $\tau = 1$ corresponds to reaching 0 and 1 in the low and high plateau over the course of the transition. T_{len} refers to the length of a transition and T_{start} to the cycle index at which the transition starts. The term cpos(c) is the position within the transition, relative to the transition center (calculated as cpos(c) = $(c-d) - T_{\text{start}} - 0.5T_{\text{len}} + 0.5$). The fractions $\frac{S}{T_{\text{len}}+1}$ and $\frac{-S}{T_{\text{len}}+1}$ are the increments from one transition cycle index to the next.

- Parameter **adaptation** τ modulates how strongly observers adapt to the distractor frequencies (with strong adaptation leading to selecting the target that shares the shape with the smaller distractor set). Zero means a flat curve that represents random choice, that is, no adaptation at all. The larger τ gets, the closer adaptation approaches that of a perfect discriminator that always selects the target feature from the smaller set. In practice, $\tau = 10$ already represents extreme adaptation, see Figure S1 (interactive) and Figure S2A.

- Parameters **shift** δ shifts the curve horizontally (for instance, for modeling lag in the update of attention control). The unit of this parameter is "trials". See Figure S1 (interactive) and Figure S2B.
- Parameter **bias** β implements a bias of the selection tendency toward one or the other shape types independent of the cycle index. Bias β is defined on the log-odds scale and varies from minus infinity to plus infinity (but extreme biases are already reached at -5 and +5, respectively). It is neutral at zero. The larger the value, the closer it approaches the exclusive selection of the decline-first shape type. The smaller the value, the closer selection approaches the exclusive selection of the other shape type. See Figure S1 (interactive) and Figure S2C.

In [5]:

```
acvsfit.interactive(phases) #, static=True)
```

```
interactive(children=(FloatSlider(value=1.0, description='Adaptation', max=10.0, step=0.01), FloatSlider
(value…
```

Figure S1. *The adaptation curve that models how the preference for the target that has the same shape as the distractors in the starting plateau changes over the cycle index. The black slopes and plateaus represent the objective relative frequency of the distractor type that has the other shape. See the main text (and main text Figure 1) for a more detailed description of the dispaly sequence over the cycle index.*

Note that for the data from Bergmann et al. (2020) which we reanalyze in the context of the present study, the cycle only consists of one and not three waves (the cycle goes from the starting plateau to the opposite plateau and back). Nevertheless, parameters obtained from these data have the same meaning and scale and can be directly compared to the experiments of the present study.

The plots blow in Figure S2 show examples for different parameter values for adaptation curves over the cycle indices of the experiments from the present study. Note that in the plots one parameter is always varied and the other two are fixed to values at which the influence of the varying paramter can be easily seen. When fitting the model to data, alls parameters are allowed to vary at the same time.

In [6]:

```
acvsfit.plot_examples(data_exp1, phases, fignum='S2', ylabel='Proportion \n "Star Selected"',
                      save='main_text_figures/Figure3.svg')
```

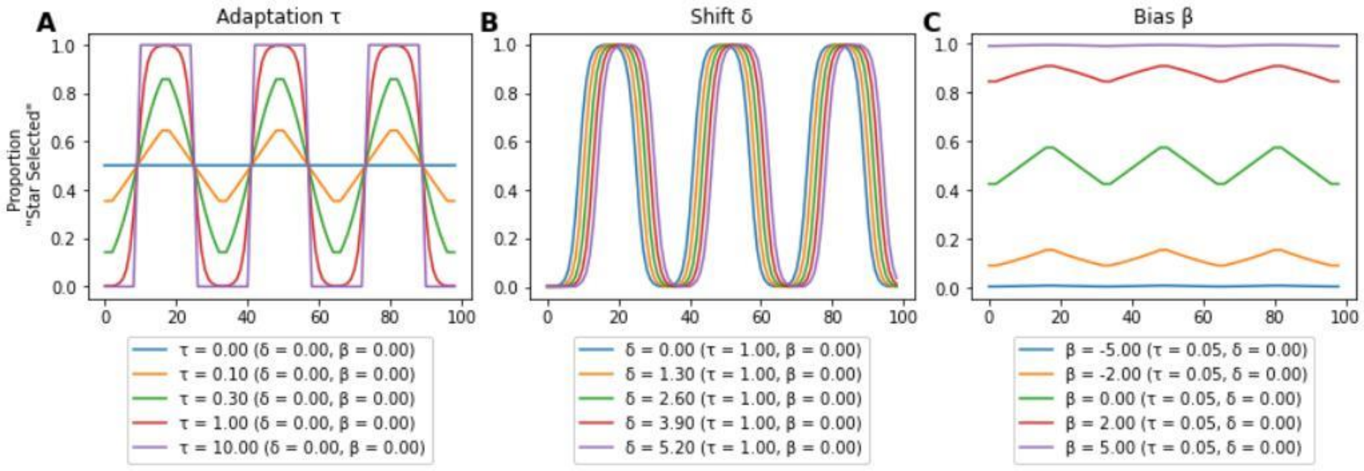


```
<IPython.core.display.Markdown object>
```

The hierarchical structure of the model is visualized below in Figure S3.

In [7]:

```
Image(filename='figures/FigureS3.png')
```

Out[7]:

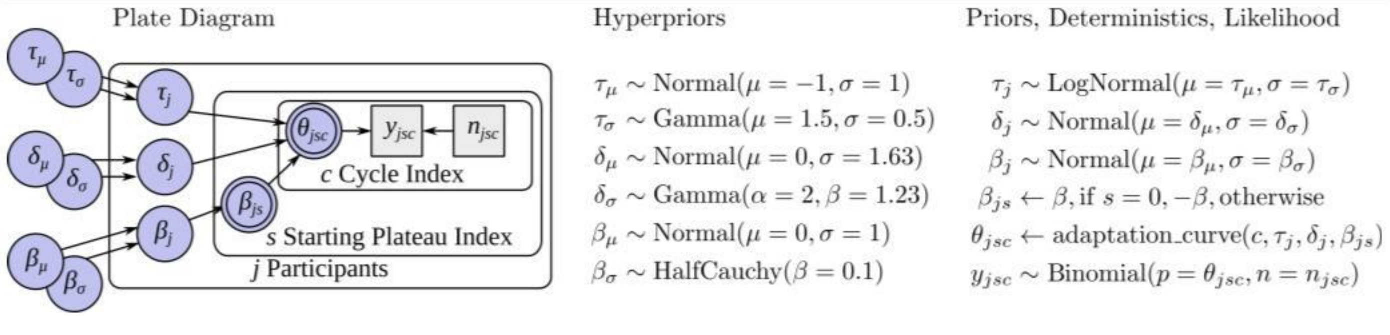

__Figure S3.__ Hierarchical model structure with (prior) distributions noted on the right. Note that the participant-level priors are implemented as non-centered parametrizations (see. e.g. McElreath, 2020). The LogNormal prior on adaptation is achieved by a non-centerd Normal wrapped in an exponential function. The plate diagram on the left follows the conventions from Lee and Wagenmakers (2014).

The cell below generate PyMC models with the cycle structure and hierarchical relationships described above.

In [8]:

```
model_exp1 = acvsfit.get_model(phases, data_exp1)
model_exp2 = acvsfit.get_model(phases, data_exp2, silent=True)
model_bergmann_exp1 = acvsfit.get_model(phases_bergmann_exp1, data_bergmann_exp1, silent=True)
model_bergmann_exp2 = acvsfit.get_model(phases_bergmann_exp2, data_bergmann_exp2, silent=True)
```

```
Auto setting shift_mu to Normal(0, %f) where SD is 1/8 of the transition length
Auto setting  shift_sigma to Gamma(α=3, β=1.23),so that its mean is 2.44
```

C. Prior distribution. The (hyper)priors are specified above in Figure S3. Below we visualize the priors these hyperpriors impose on the participant level (Figure S4).

Prior explanations & justifictaions:

The priors are default priors implemented in the current version (0.1.0) of acvsfit which were selected to be vague to let typical ACVS data covern the results. On the other hand they restrict the prior probability in to the theoretically meaningful an plausible ranges.

- Adaptation $\tau_\mu \sim$ Normal($\mu = -1, \sigma = 1$) and $\tau_\sigma \sim$ Gamma($\mu = 1.5, \sigma = 0.5$): These hyperpriors feed into the LogNormal prior on the participant level. They assign high probabilities to positive values, particularly in the range between 0 and 0.5, but there is also a long tail toward higher values (cf. Figure S4A). ACVS experiments in the literature typically contain weak to medium adaptation with the average curve alternating between 0.4 and 0.6 (the full range is 0 to 1; e.g. Irons & Leber, 2016; Irons & Leber, 2018; Bergmann et al., 2020) which roughly corresponds to a value 0.07 for our adaptation parameter; hence the range covered by the prior is very wide compared to such values. Nevertheless, large values of substantial adaptation (e.g., $\tau = 1$, where the curve reaches the plateaus over the course of the transition) and very high adaptation (e.g.,$\tau = 10$, approaching that of a perfect discriminator) are possible as well. Figure S5B shows adaptation curves drawn from the prior. As can be seen, the whole range from very flat curves with practically no adaptation to very rectangular curves of extremely high adaptation is included. Note that the prior is in the positive range only. Adaptation curves with negativeτ would be inverted with respect to the cycle. That is, selection probability would be high in plateaus for a target that has the same feature as all distractors and which does not stand out from its context. This is typically not observed, and there is no plausible mechanism that would lead to such selection behavior. Hence, the negative range is excluded from our prior adaptation (see Section 2.5 for a sensitivity analysis that looks at this).
- Shift $\delta\mu \sim$ Normal($\mu = 0, \sigma = 1.625$) and $\delta\sigma \sim$ Gamma($\alpha = 3, \beta = 9.84$):The prior on shift depends on the length of the transition. The group-level mean is a normal distribution centered at zero (hence neutral with respect to left- or rightward shifts), and its standard deviation is set$T_{Len}/8$, whereT_{Len} is the length of the transition. That is, the bulk of the prior density (2 SDs) ranges from minus a quarter of the transition to plus a quarter, or-3.25, to3.25 trials. The Gamma($\mu = 3$, $\sigma = 9.84$) ($\sigma = 9.84$ corresponds to 1/($T_{Len}/16$)) was selected because it establishes an early peaking but long-tailed prior on dispersion which is bent away from zero. The latter helps to avoid implosive shrinkage (Liddell & Kruschke, 2018 (Liddell2018); also see Section 2.5 of this report). As can be seen in Figure S4B, the prior covers a wide range of shifts on the participant level that go roughly up to half the transition length. Figure S5C illustrates adaptation curves drawn from the prior.
- Bias $\beta_\mu \sim$ Normal($\mu = 0, \sigma = 1$) and $\beta_\sigma \sim$ HalfCauchy($\alpha = 0.1$): The range of biases that can be expected in ACVS depends strongly on the concrete experiments. In our experiments with different shapes and Bergmann et al.'s (2020) experiments with different colors, we expect only slight biases toward one or the other shape (color). Nevertheless, we stick to acvsfits default prior on the bias parameter.β_μ is neutrally centered at zero (no bias), and the bulk of the distribution (2 SDs) ranges from -2 to 2, which already represents very strong biases (cf. Figure S2C). Figure S3C depicts the priors imposed on the participant level). The prior contains adaptation curves shifted all the way up or down (see Figure S5D)

In sum, all priors are selected to be vague and let the data speak. After sampling from the models (see Section 2.2), plots of prior vs. posterior probability confirmed that the former were vague with respect to the latter (see Figure S11).

In [9]:

```
acvsfit.plot_priors(model_exp1, random_seed)
```

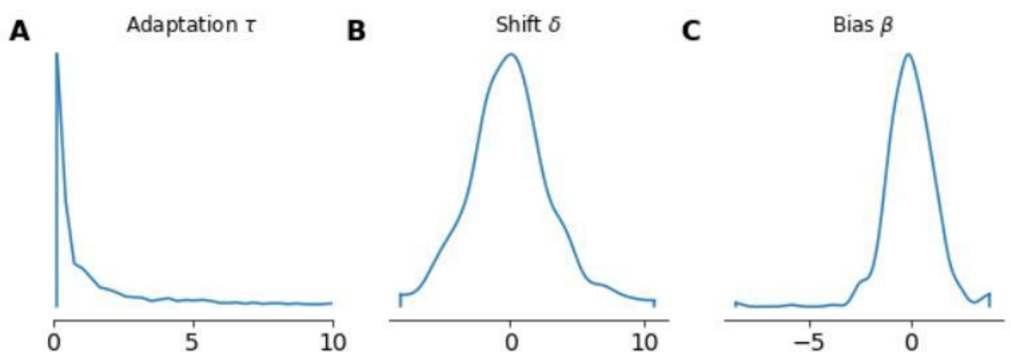

Figure S4. *Prior distributions imposed on the participant-level by the hyperpriors. Distributions are truncated at their 99% HDIs (and in pandel A the axsis is limited to the range from 0 to 10, to improve visibility of the distribution).*

D. Formal specification. A formal specification was already provided above in section 2.1B. Moreover, the source code that implements the adaptation curve and sets up the hierarchical PyMC model can be found here: https://github.com/jt-lab/acvsfit/blob/v0.1.0/src/acvsfit/acvsfit.py#L74 (https://github.com/jt-lab/acvsfit/blob/v0.1.0/src/acvsfit/acvsfit.py#L74)

E. Prior predictive check. Prior predictive simulations have already been mentioned referring to panels **B** to **D** of Figure S5 below. Panel **A** shows curves with prior samples used for all parameters. It can be seen that a wide range of possible adaptation curves is covered by the prior.

In [10]:

```
acvsfit.plot_prior_simulations_quartet(model_exp1, phases, random_seed)
```

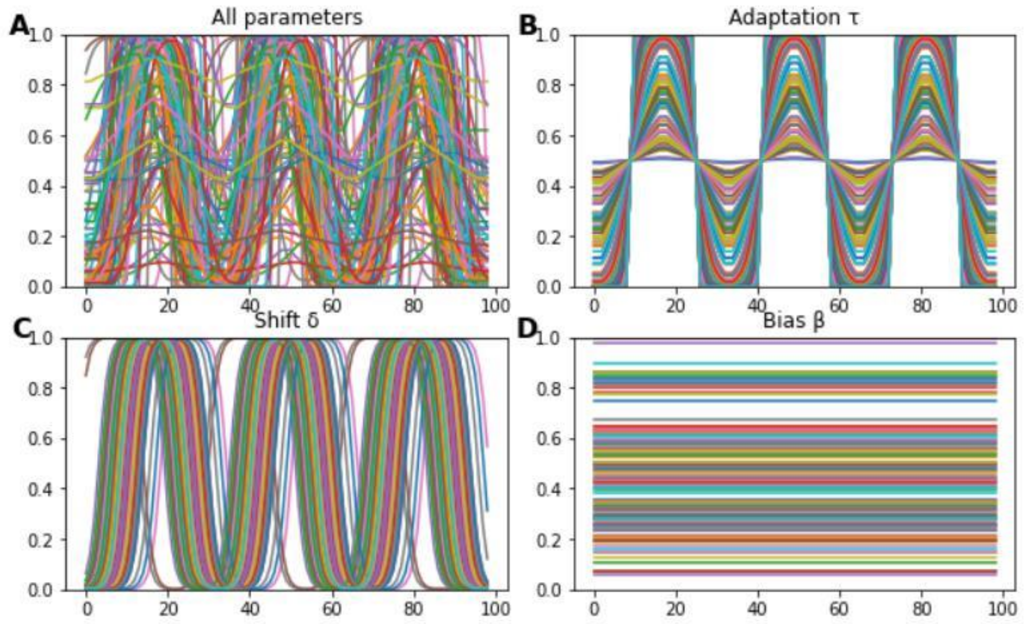

Figure S5. *Prior predictive simulations that show 100 adaptation curves drawn from the prior. Panel* ***A*** *shows the full curves with all parameters based on the prior samples. Panel* ***B*** *illustrated adaptation with samples from the prior but shift and bias were fixed to zero. Panel* ***C*** *shows the curves with the shifts from the prior but with adaptation fixed to one and bias to zero. Finally, panel* ***D*** *illustrates the bias based on the prior samples with adaptation and shift fixed to zero. Figure style adapted from Tünnermann (2022), CC BY 4.0.*

2.2 Details of the computation (BARG Step 2)

A. Software. The cell below uses the acvsfit framework to sample from the posterior. The combination of default parameters and the explicitly stated ones set up the sampling in the following way:

- Samples: NUTS (Hoffmann & Gelman, 2014)
- Initalization: jitter+adapt_diag (see here (https://docs.pymc.io/en/v3/api/inference.html))
- Tuning Samples: 1000

- Target Accept Rate: 0.75
- Samples per chain (after tuning): 20000
- Chains: 4
- Thinning (applied to reduce file sizes): Factor 4, leaving 5000 samples per chain

Note that the calls to `acvsfit.get_samples` below check whether a file (netCDF format) with samples already exists. If this is the case, all arguments are ignored and the canned samples are loaded. This allows to run the subsequent analysis steps without re-sampling every time.

In [11]:

```
trace_exp1 = acvsfit.get_samples(model_exp1, samples=20000, thin=4,
                         file='traces/MuEtAl2022-Exp1.nc', seed=random_seed)
trace_exp2 = acvsfit.get_samples(model_exp2, samples=20000, thin=4, silent=True,
                         file='traces/MuEtAl2022-Exp2.nc', seed=random_seed)
trace_bergmann_exp1 = acvsfit.get_samples(model_bergmann_exp1, samples=20000, silent=True,
                         thin=4, file='traces/BergmannEtAl2020-Exp1.nc', seed=random_seed)
trace_bergmann_exp2 = acvsfit.get_samples(model_bergmann_exp2, samples=20000,silent=True,
                         thin=4, file='traces/BergmannEtAl2020-Exp2.nc', seed=random_seed)
```

```
Loading samples from disk! Delete/rename the existing file or change "file" argument in get_samples if yo
u want to fit anew instead of loading.
```

B. MCMC chain convergence. As can be seen below in Figure S6, the chains have properly mixed and converged. Moreover, the Gelman-Rubin's R-Hat statistic (see Table S1) is close 1, indicating proper convergence.

C. MCMC chain resolution. Table S1 below contains descriptions of the ESS (effective sample size) values. Extensive tables with values for each parameter can be found in subfolder "summaries", generated by the cell further down. The ESS for the calculated differences can be seen below in Section 2.3B. Several thousand (and at least more than 1000 in the weakest case) effective samples are contained in the chains, providing proper resolution for the posterior distribution.

In [12]:

```
acvsfit.plot_trace(trace_exp1, 'Experiment 1')
```

Experiment 1

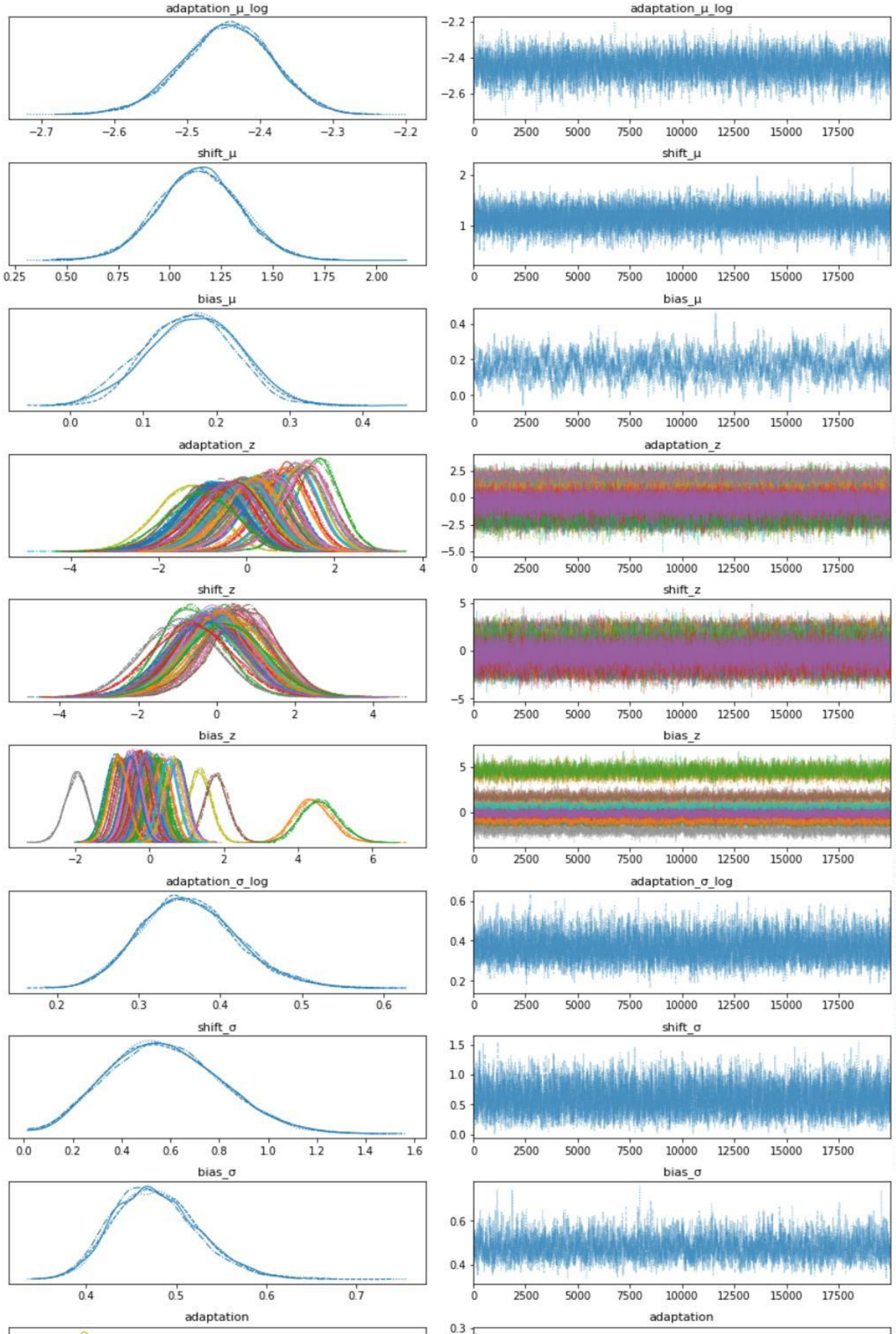

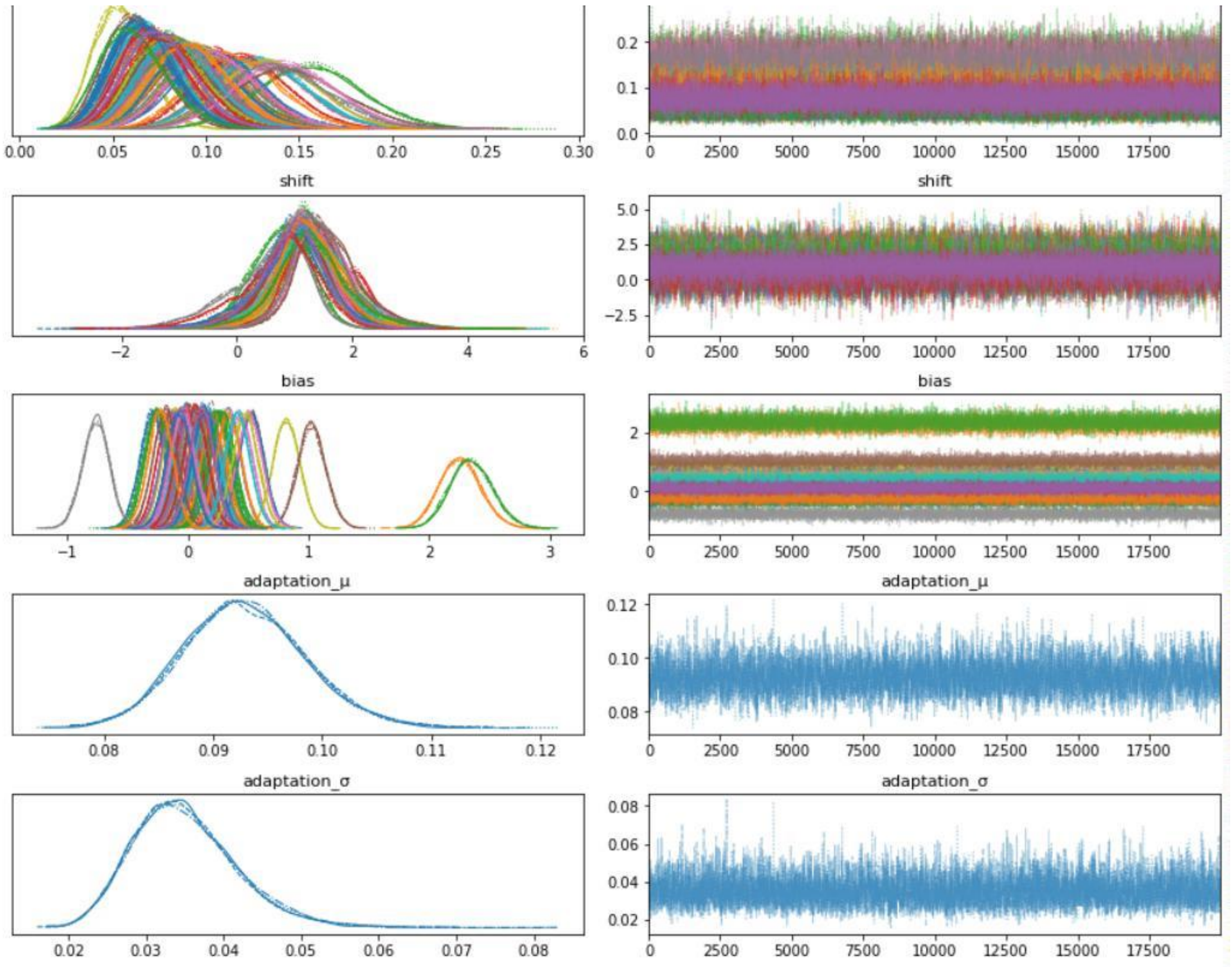

Figure S6A. *Trace plots for Experiment 1. Please view digitally with zoom if necessary.*

In [17]:
```
acvsfit.plot_trace(trace_exp2, 'Experiment 2')
```

Experiment 2

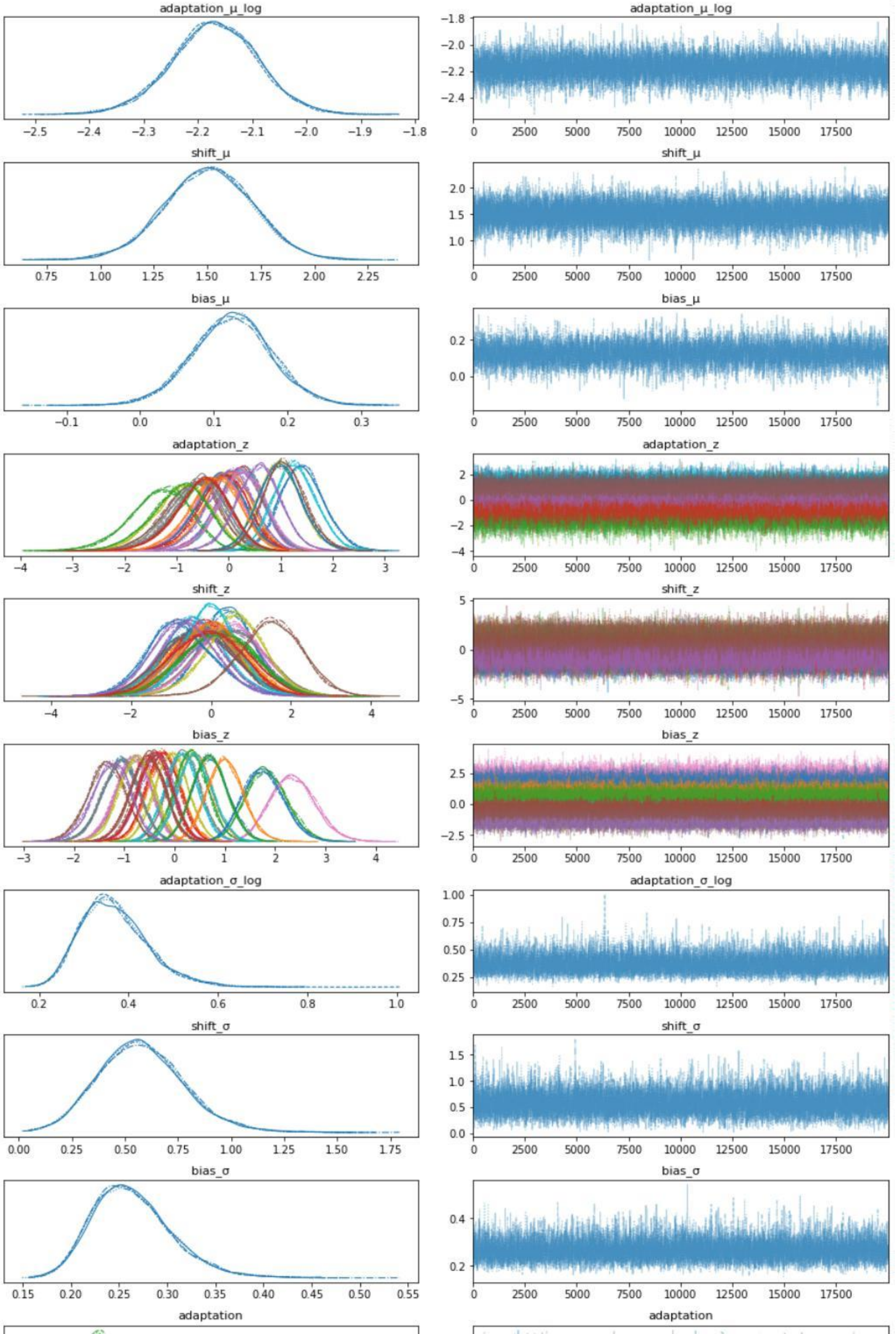

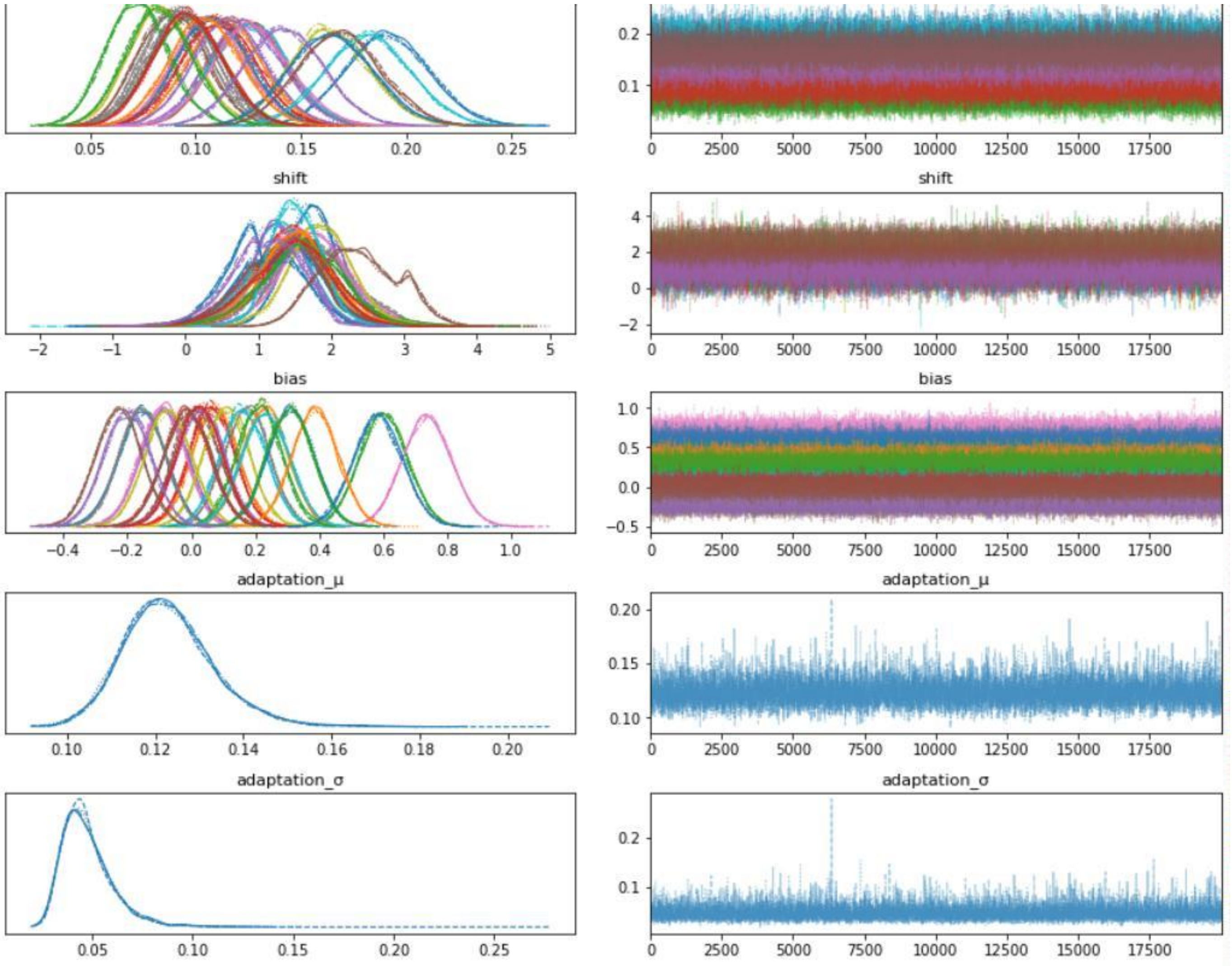

Figure S6B. *Trace plots for Experiment 2. Please view digitally with zoom if necessary.*

In [14]:

```
acvsfit.plot_trace(trace_bergmann_exp1, "Bergmann et al's Experiment 1")
```

Bergmann et al's Experiment 1

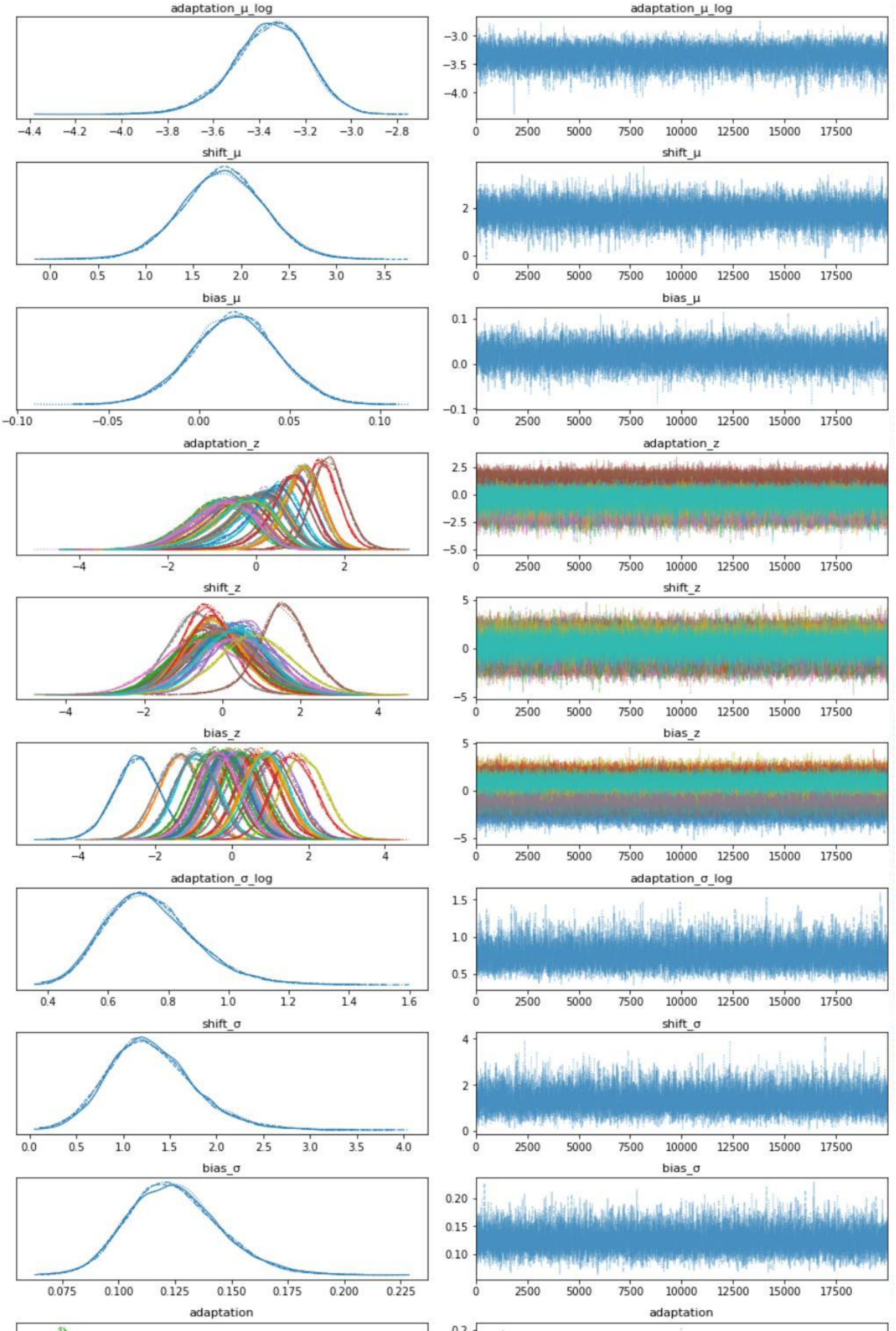

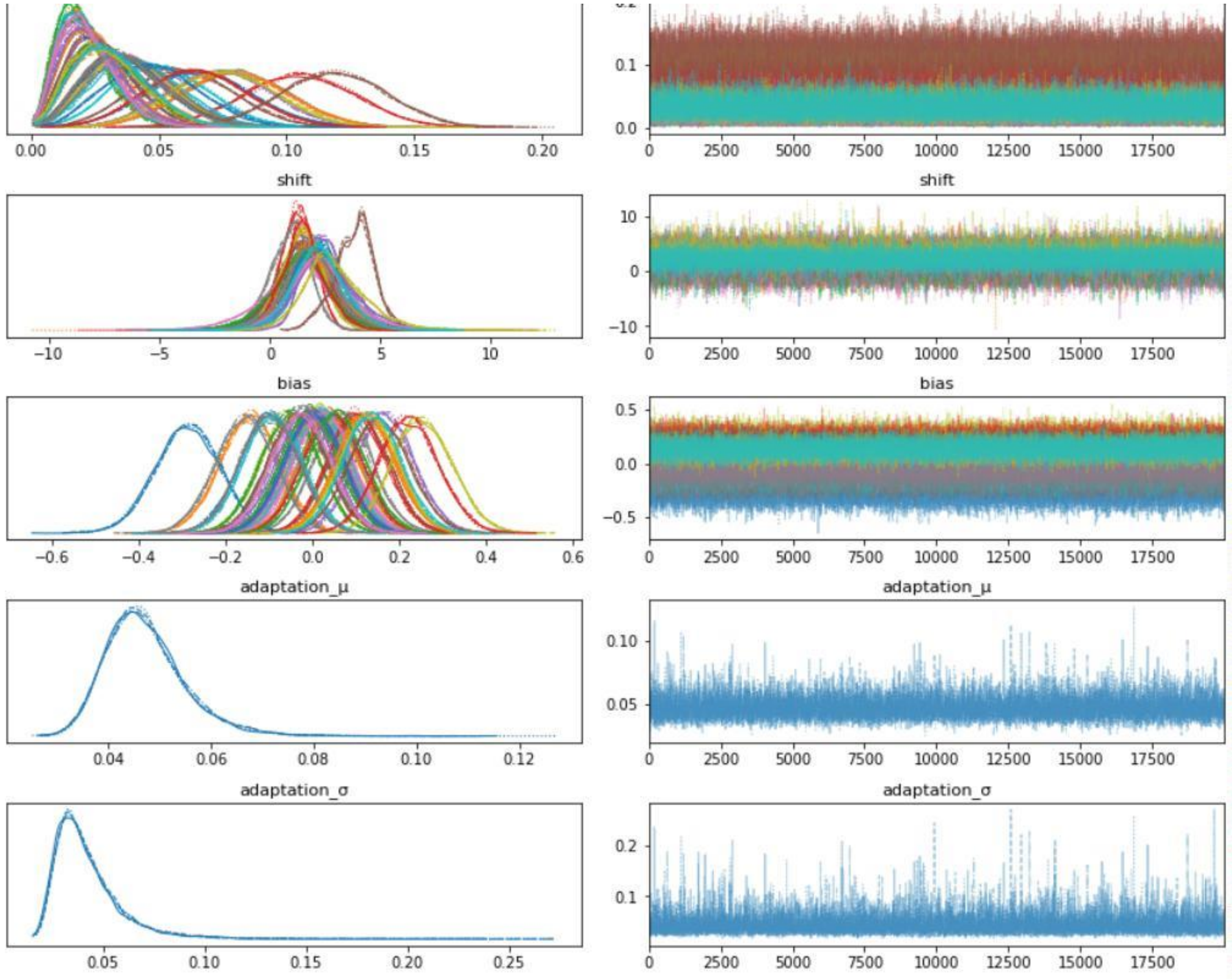

Figure S6C. *Trace plots for Bergmann et al.'s Experiment 1. Please view digitally with zoom if necessary.*

In [15]:
```
acvsfit.plot_trace(trace_bergmann_exp2, "Bergmann et al's Experiment 2")
```

Bergmann et al's Experiment 2

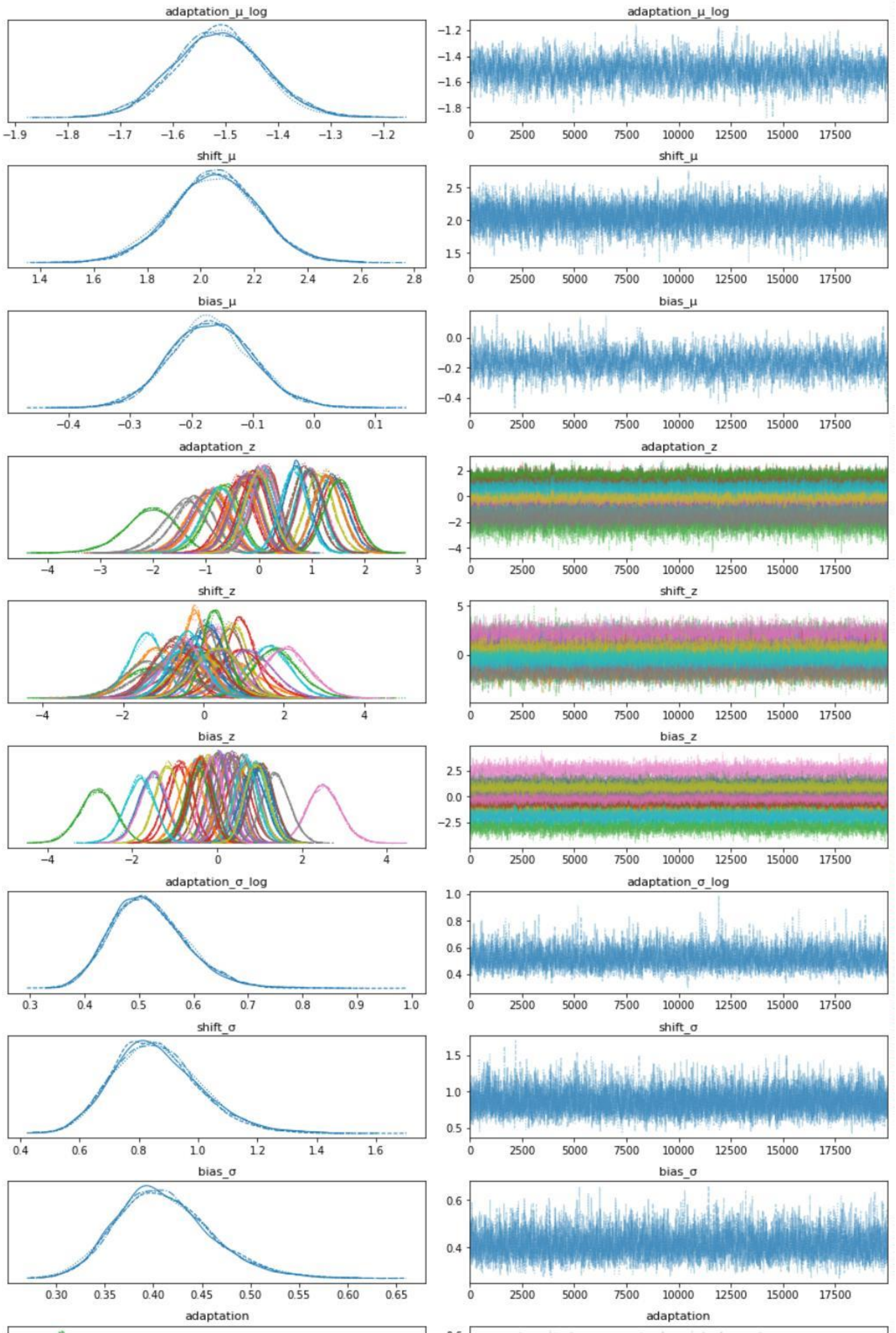

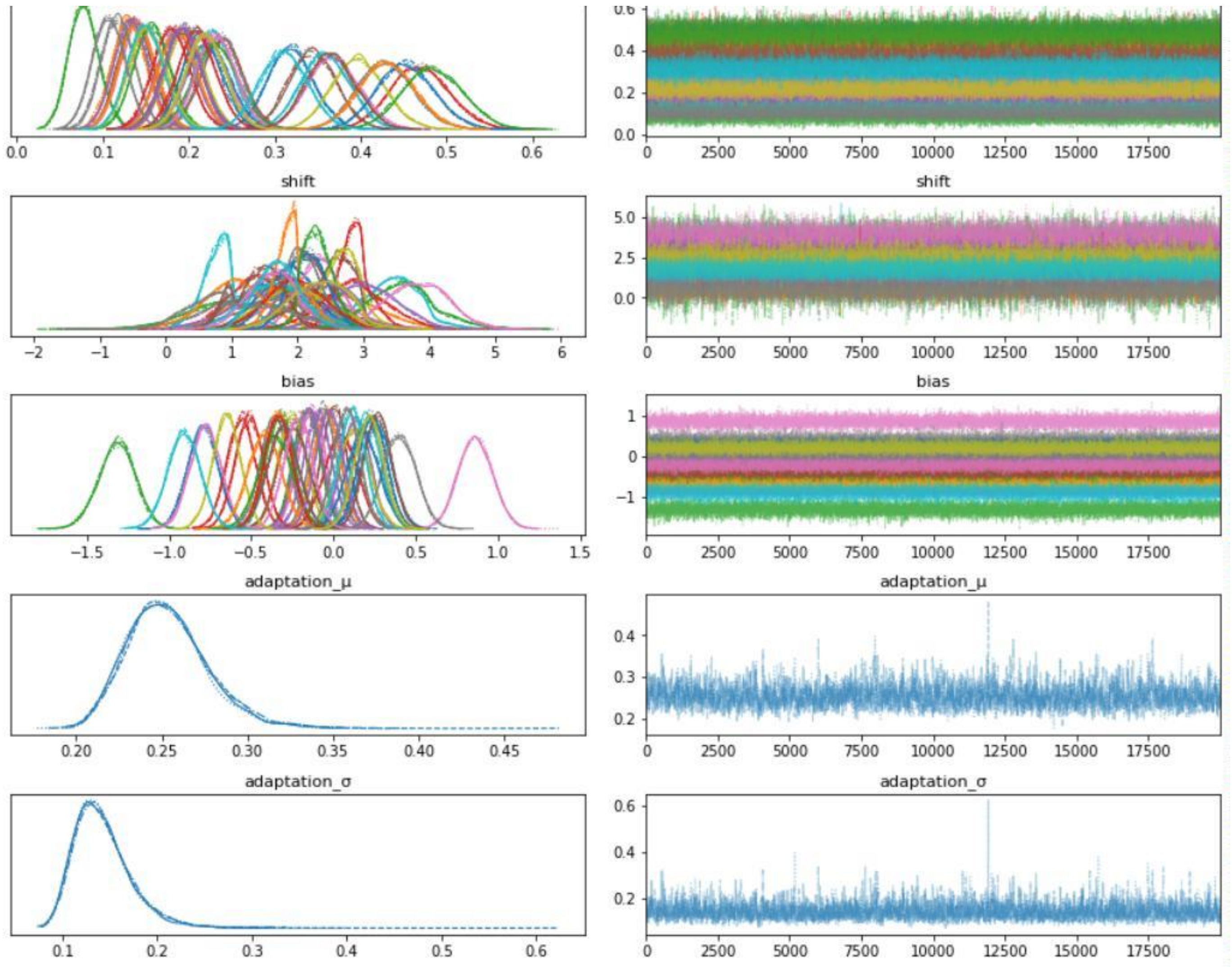

Figure S6D. *Trace plots for Bergmann et al.'s Experiment 2. Please view digitally with zoom if necessary.*

In [16]:

```
display(pd.DataFrame.merge(acvsfit.get_diagnostic(trace_exp1),
                   acvsfit.get_diagnostic(trace_exp2),
                   left_index=True, right_index=True, suffixes=['Exp1', 'Exp2']))
display(pd.DataFrame.merge(acvsfit.get_diagnostic(trace_bergmann_exp1),
                   acvsfit.get_diagnostic(trace_bergmann_exp2),
                   left_index=True, right_index=True, suffixes=['_BergExp1', '_BergExp2']))
```

	ess_bulkExp1	ess_tailExp1	r_hatExp1	ess_bulkExp2	ess_tailExp2	r_hatExp2
**count**	398.000000	398.000000	398.000000	164.000000	164.000000	164.0
**mean**	8010.864322	11579.226131	1.000025	16238.792683	17447.286585	1.0
**std**	3139.051397	3223.454246	0.000501	3312.204971	1653.295426	0.0
**min**	517.000000	1300.000000	1.000000	6014.000000	10070.000000	1.0
**25%**	7484.750000	10606.000000	1.000000	14614.250000	16697.500000	1.0
**50%**	8926.500000	12375.500000	1.000000	17873.500000	17950.500000	1.0
**75%**	9986.500000	13751.250000	1.000000	18570.500000	18660.000000	1.0
**max**	13915.000000	17686.000000	1.010000	19933.000000	19615.000000	1.0

	ess_bulk_BergExp1	ess_tail_BergExp1	r_hat_BergExp1	ess_bulk_BergExp2	ess_tail_BergExp2	r_hat_BergExp2
**count**	248.000000	248.000000	248.0	248.000000	248.000000	248.0
**mean**	18972.229839	18854.379032	1.0	8253.903226	10867.814516	1.0
**std**	1020.484004	657.613303	0.0	4008.457913	3602.904493	0.0
**min**	13566.000000	13957.000000	1.0	1308.000000	2581.000000	1.0
**25%**	18708.500000	18612.250000	1.0	3783.500000	7465.000000	1.0
**50%**	19160.500000	18968.000000	1.0	9281.000000	11855.500000	1.0
**75%**	19566.000000	19261.000000	1.0	11589.250000	13683.250000	1.0
**max**	20371.000000	19849.000000	1.0	16101.000000	17877.000000	1.0

Table S1. *Descriptions of diagnostic values for Experiment 1 and 2 (suffixes Exp1 & Exp2) of the present study and Bergmann et al.'s Experiment 1 and 2 (suffixes BergExp1 & BergExp2). r_hat = Gelman-Rubin's R-Hat statistic. Indicates convergence if close to 1; ess = effective sample size, indicate how many samples are approximately available if one accounts for autocorrelation within the chains.*

To save extensive tables with one row per varbiable with estimates and diagnostics to file, excecute the cell below. This is extremely time consuming, hence the `%%script echo skipping` was added to skip the cell by default. Remove it to execute the cell.

In [17]:

```
%%script echo skipping
def mode(values):
    return az.plots.plot_utils.calculate_point_estimate('mode', values)
az.summary(trace_exp1,  stat_funcs=[mode], extend=True )\
                                    .to_csv('summaries/summary_exp1.csv')
az.summary(trace_exp2, stat_funcs=[mode], extend=True)\
                                    .to_csv('summaries/summary_exp2.csv')
az.summary(trace_bergmann_exp1, stat_funcs=[mode], extend=True)\
                                    .to_csv('summaries/summary_bergmann_exp1.csv')
az.summary(trace_bergmann_exp2, stat_funcs=[mode], extend=True)\
                                    .to_csv('summaries/summary_exp1.csv')
```

```
skipping
```

2.3 Posterior Description (BARG Step 3)

A. Posterior Predictive Check. Figure 7 below shows posterior predictive check plots on the group level and Figure 8 show participant-level plots. The plots show that the model is able to capture the patterns in the data.

In [18]:

```
acvsfit.plot_group_ppc(trace_exp1, data_exp1, phases, colors=colors,
                                            labels=['A'], label_every_nth=2,
                                            save='main_text_figures/PostPredExp1.svg')
acvsfit.plot_group_ppc(trace_exp2, data_exp2, phases, colors=colors, labels=['B'],
                                            save='main_text_figures/PostPredExp2.svg')

f,ax = plt.subplots(1,2, figsize=(15,3))
acvsfit.plot_group_ppc(trace_bergmann_exp1, data_bergmann_exp1,
                              phases_bergmann_exp1, colors=colors, ax=ax[0], labels=['C'])
acvsfit.plot_group_ppc(trace_bergmann_exp2, data_bergmann_exp2,
                              phases_bergmann_exp2, colors=colors, ax=ax[1], labels=['D'])
```

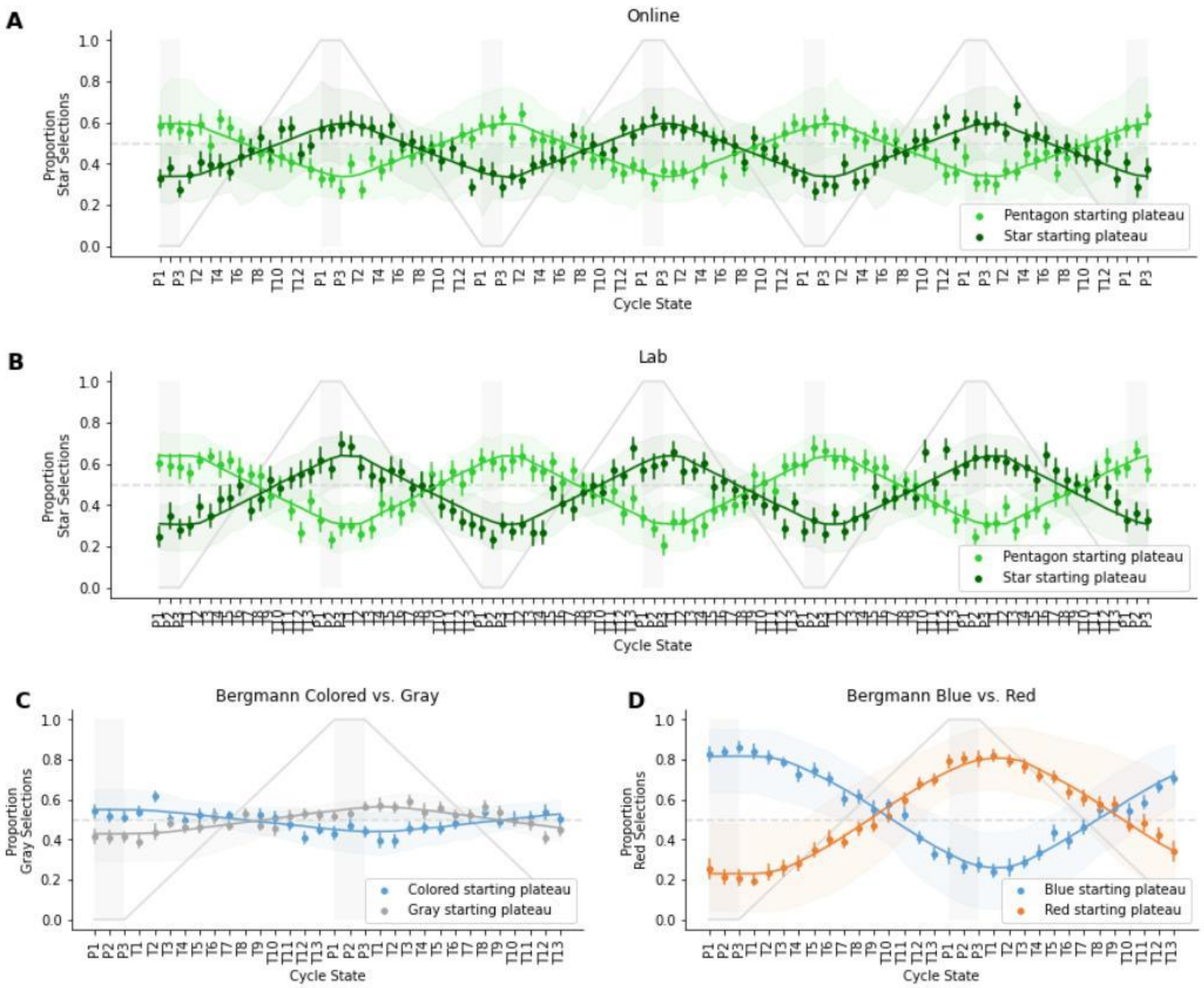

Figure S7. *Posterior predictive checks on the group level for Experiments 1 and 2 of the present study (**A** & **B**) and for Bergmann et al's (2020) free choice data (**C** & **D**). Points aggregated data as the proportion of the 'star' ('gray', 'red') selections with standard error of the mean error bars. The solid lines show the adaptation curves based on the posterior (mean of predicted selections). The shaded areas are based on 95 % HDIs of the predicted selection distributions averaged over all participants at each cycle state. The solid gray line illustrates the objective relative frequency of the 'Pentagon' distractors. The dashed gray line marks the point .5-level, which corresponds to random selections. The rectangular gray areas mark plateaus. Tick labels P1 to P3 mark trials 1 to 3 within the plateaus, and T1 to T13 mark the 13 trials of the transitions. Figure style adapted from Tünnermann (2022), CC BY 4.0.*

In [19]:

```
acvsfit.plot_participant_ppc(trace_exp1, data_exp1, phases, colors=colors)
```

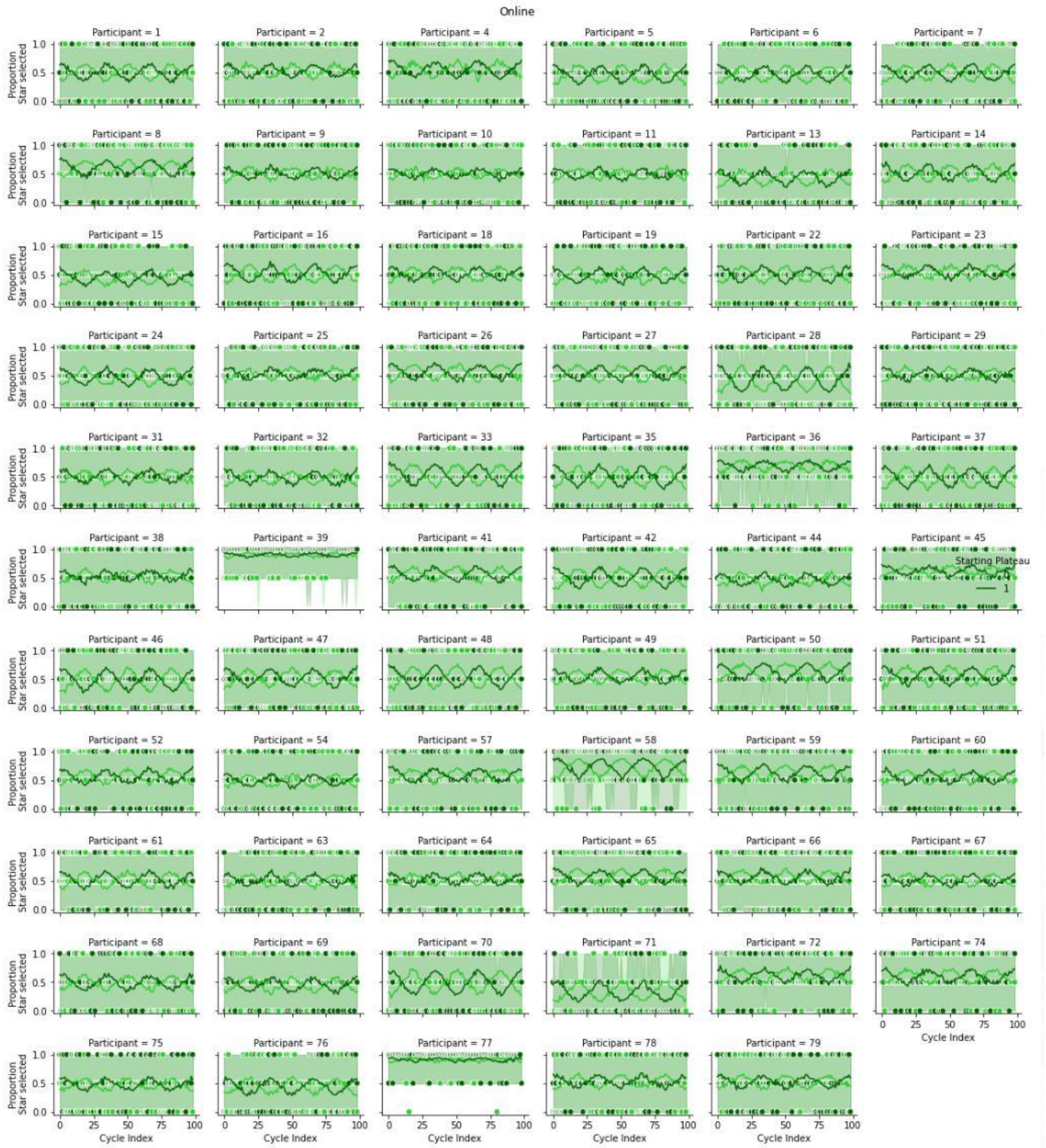

Figure S8A. *Posterior predictive checks on the participant level for Experiment 1. One plot per participant. Points show the proportion of the 'star' ('gray', 'red') selections . The shaded areas are based on 95 % HDIs of the predicted (relative) selections. Please view digitally with zoom if necessary. Figure style adapted from Tünnermann (2022), CC BY 4.0.*

Note: In Experiment 1, there are only three repetitions per cycle index. Hence, the means are 0, 0.5, or 1, and it is difficult to see that the predicted adaptation curves agree with these values. But it can be seen that for extreme cases such as participant 39 or 77, the adaptation curve correctly captures the strong biases that lead to an almost exclusive selection of one shape type. For Experiment 2 (Figure S8B) and Bergmann et al.'s (2020) data (Figure S8C & D), it is more evident that the model follows the selection trends in the data. Given the coarse data of Experiment 1, the matching fits of Experiment 2 and the Bergmann data, and reasonable average curves in Figure 7, there is no reason to distrust the model predictions for Experiment 1.

In [20]:

```
acvsfit.plot_participant_ppc(trace_exp2, data_exp2, phases, colors=colors)
```

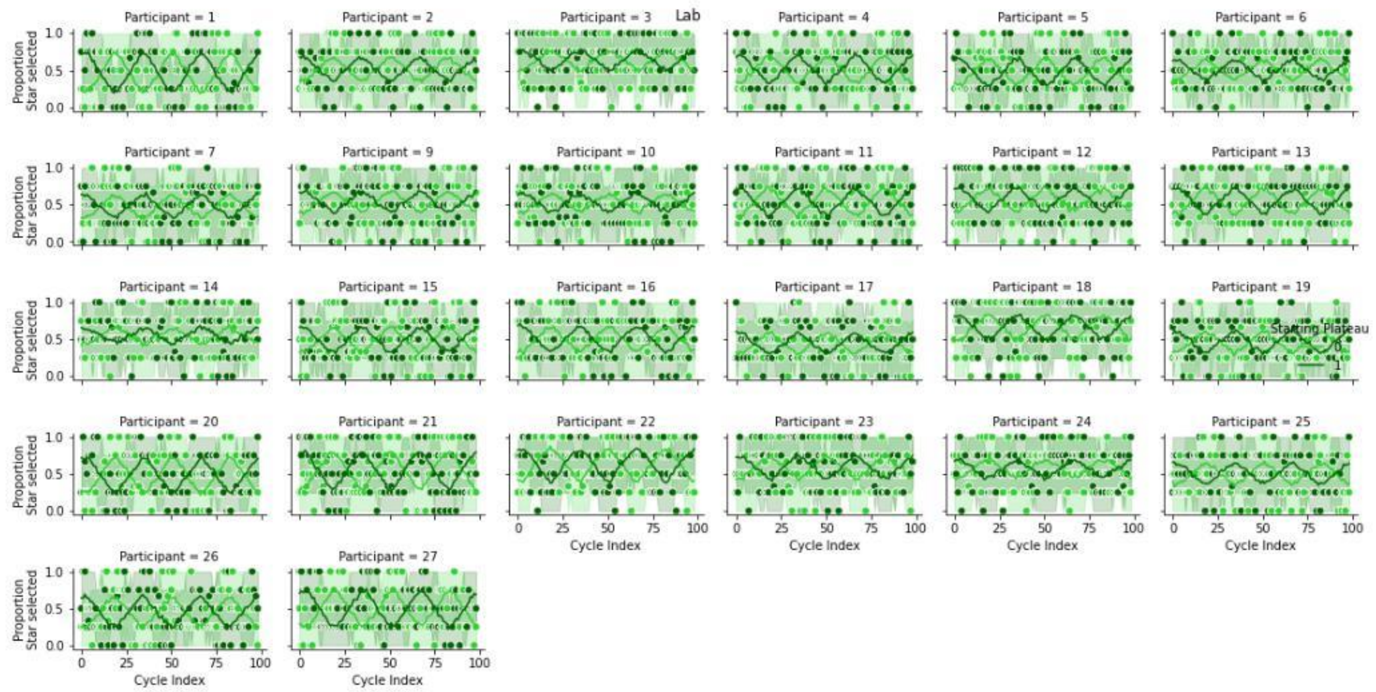

Figure S8B. *Posterior predictive checks on the participant level for Experiment 2. One plot per participant. Points show the proportion of the 'star' ('gray', 'red') selections . The shaded areas are based on 95 % HDIs of the predicted (relative) selections. Please view digitally with zoom if necessary.Figure style adapted from Tünnermann (2022), CC BY 4.0.*

In [21]:

```
acvsfit.plot_participant_ppc(trace_bergmann_exp1, data_bergmann_exp1, phases, colors=colors)
```

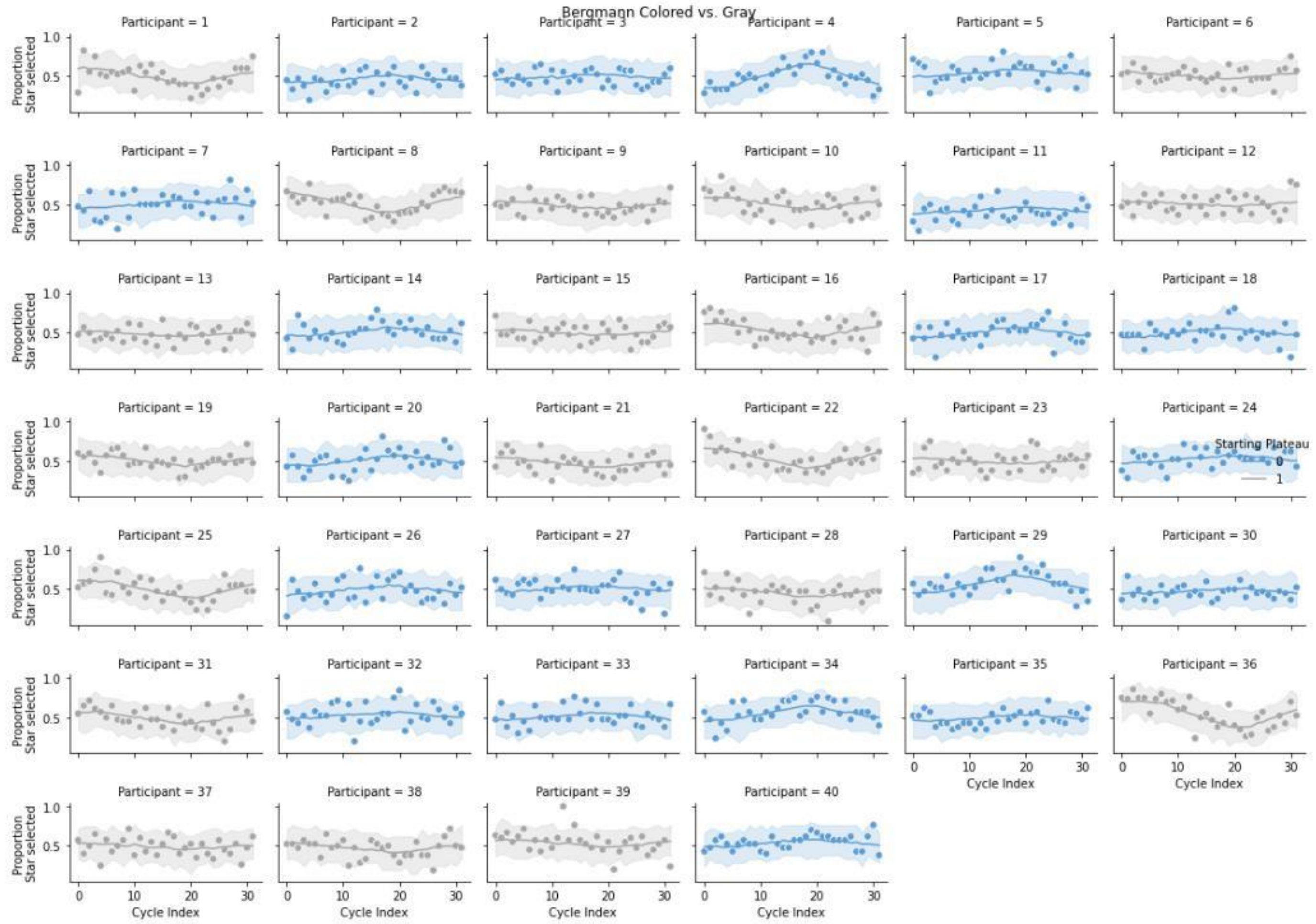

Figure S8C. *Posterior predictive checks on the participant level for Bergmann et al.'s Experiment 1. One plot per participant. Points show the proportion of the 'star' ('gray', 'red') selections . The shaded areas are based on 95 % HDIs of the predicted (relative) selections. Please view digitally with zoom if necessary.Figure style adapted from Tünnermann (2022), CC BY 4.0.*

In [22]:
```
acvsfit.plot_participant_ppc(trace_bergmann_exp2, data_bergmann_exp2, phases, colors=colors)
```

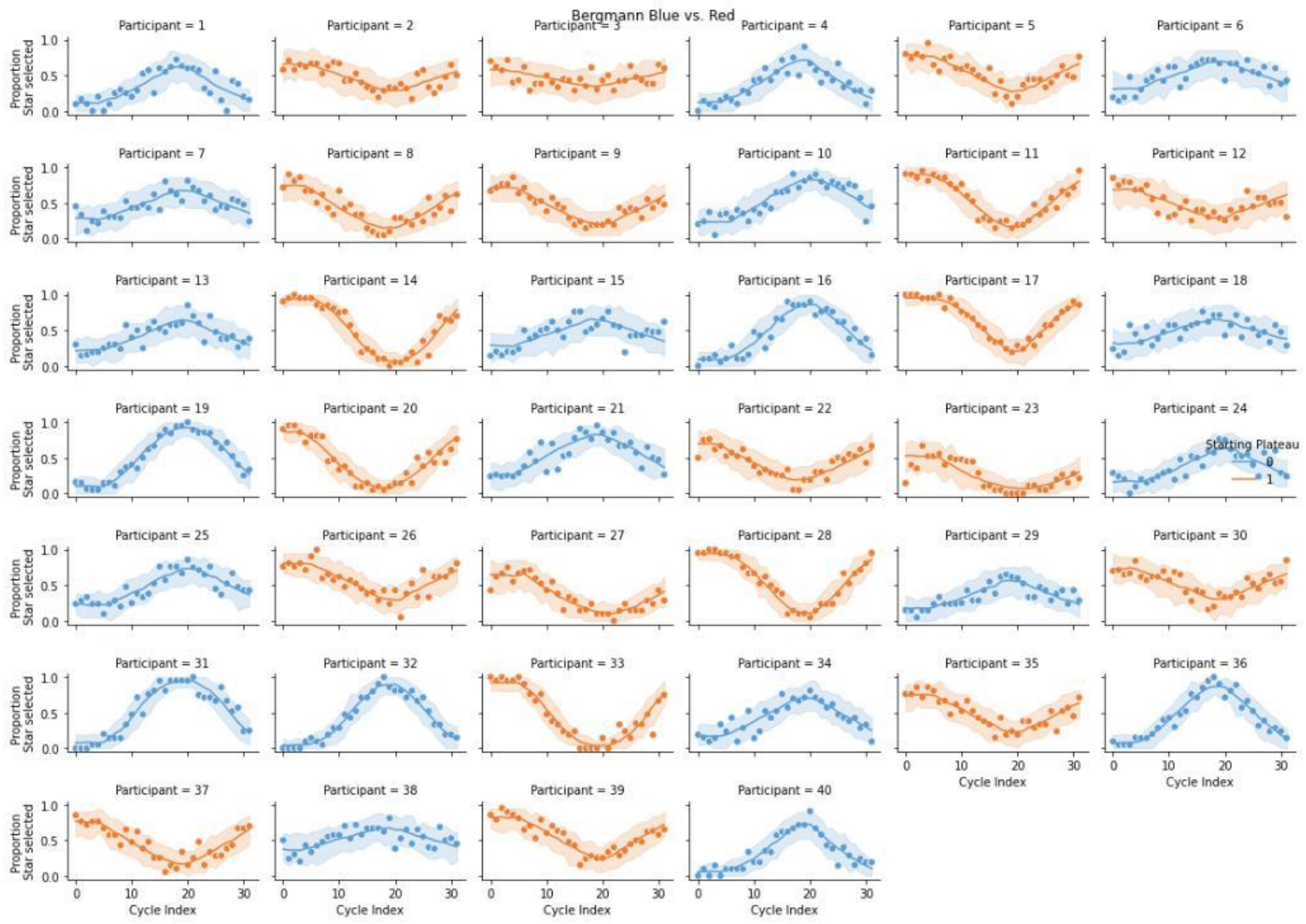

Figure S8D. *Posterior predictive checks on the participant level for Bergmann et al.'s Experiment 2. One plot per participant. Points show the proportion of the 'star' ('gray', 'red') selections . The shaded areas are based on 95 % HDIs of the predicted (relative) selections. Please view digitally with zoom if necessary.Figure style adapted from Tünnermann (2022), CC BY 4.0.*

B. Summarize posteriors of variables.

In [23]:

```
f, axs = plt.subplots(4,3, figsize=(14,14), sharex='col')
acvsfit.plot_group_posteriors(trace_exp1, data_exp1, colors=colors, ax=axs[0,:],save='figures/trace_ex
acvsfit.plot_group_posteriors(trace_exp2, data_exp2, colors=colors, ax=axs[1,:],save='figures/trace_ex
acvsfit.plot_group_posteriors(trace_bergmann_exp1, data_bergmann_exp1, colors=colors, ax=axs[2,:])
axs[2,0].set_ylabel('Bergmann \n Colored vs- Gray')
acvsfit.plot_group_posteriors(trace_bergmann_exp2, data_bergmann_exp2, colors=colors, ax=axs[3,:])
_ = axs[3,0].set_ylabel('Bergmann \n Blue vs- Red')
```

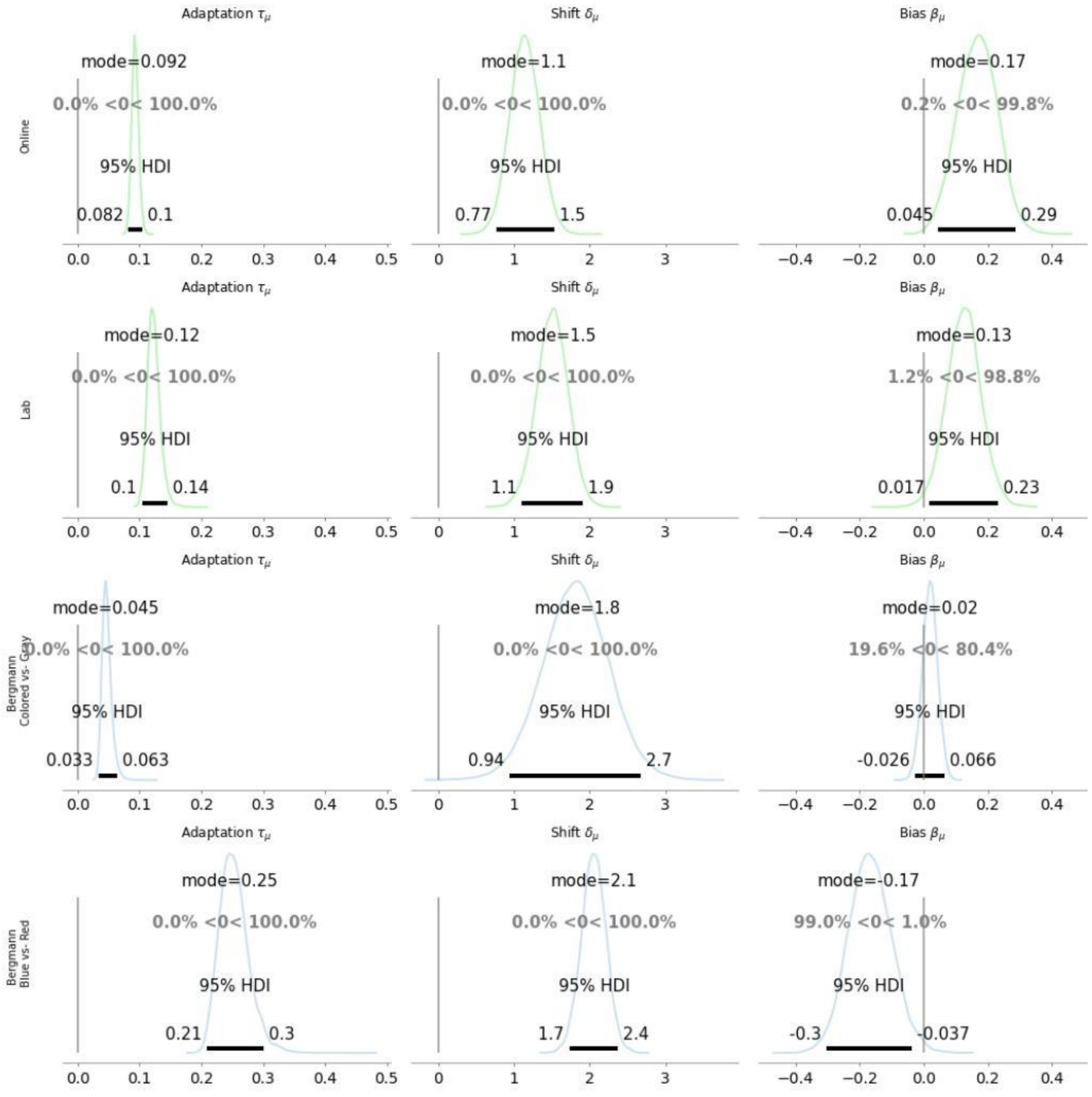

Figure S9. Posterior distributions of the group-level estimates of adaptation τ_μ , shift δ_μ , and bias β_μ for Experiment 1 (Online), Experiment 2 (Lab), and Bergmann et al.'s (2020) Experiment 1 ("colored vs. gray") and Experiment 2 ("blue vs. red").

In [24]:

```
acvsfit.plot_participant_posteriors(trace_exp1, data_exp1, colors=colors)
```

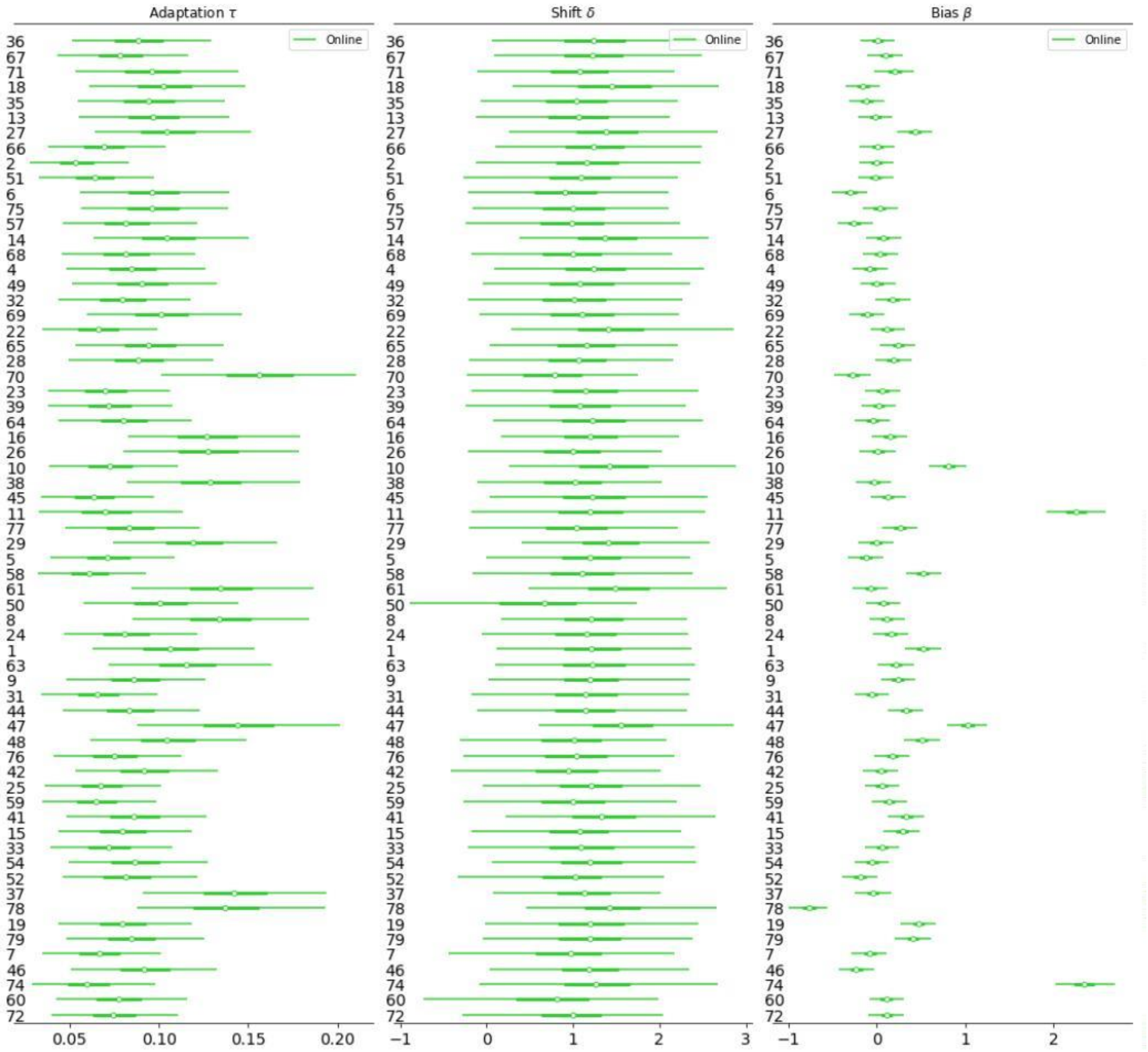

Figure S10A. *Posterior distributions of the participant-level estimates of adaptation τ, shift δ, and bias β for Experiment 1 (Online).*

In [25]:

```
acvsfit.plot_participant_posteriors(trace_exp2, data_exp2, colors=colors)
```

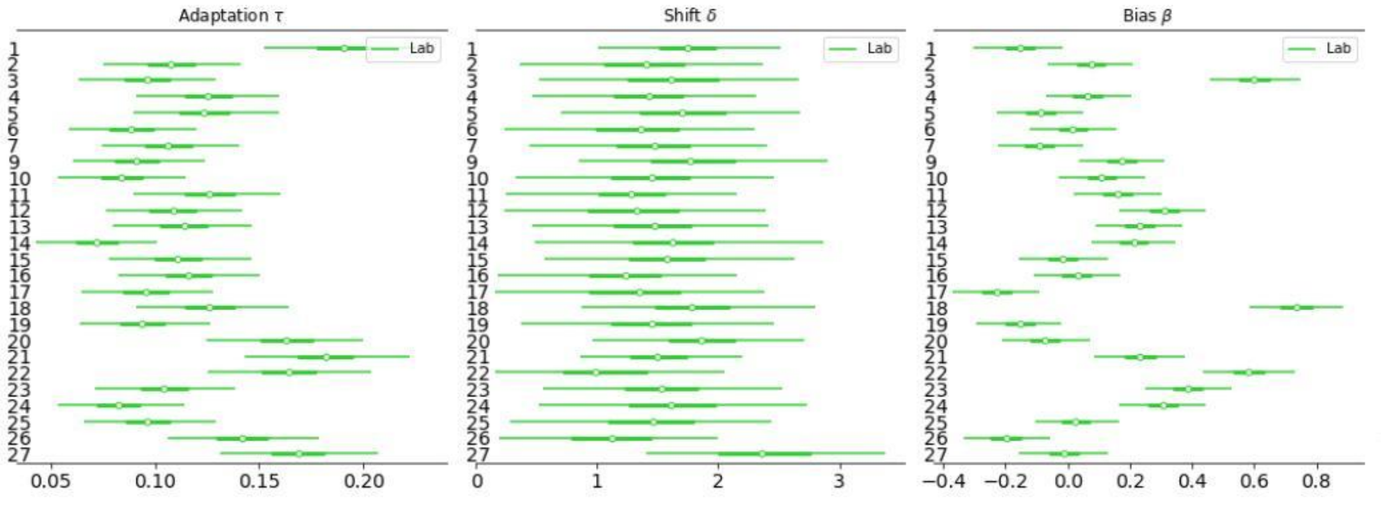

Figure S10B. *Posterior distributions of the participant-level estimates of adaptation τ , shift δ , and bias β for Experiment 2 (Lab).*

In [26]:
```
1  acvsfit.plot_participant_posteriors(trace_bergmann_exp1, data_bergmann_exp1, colors=colors)
```

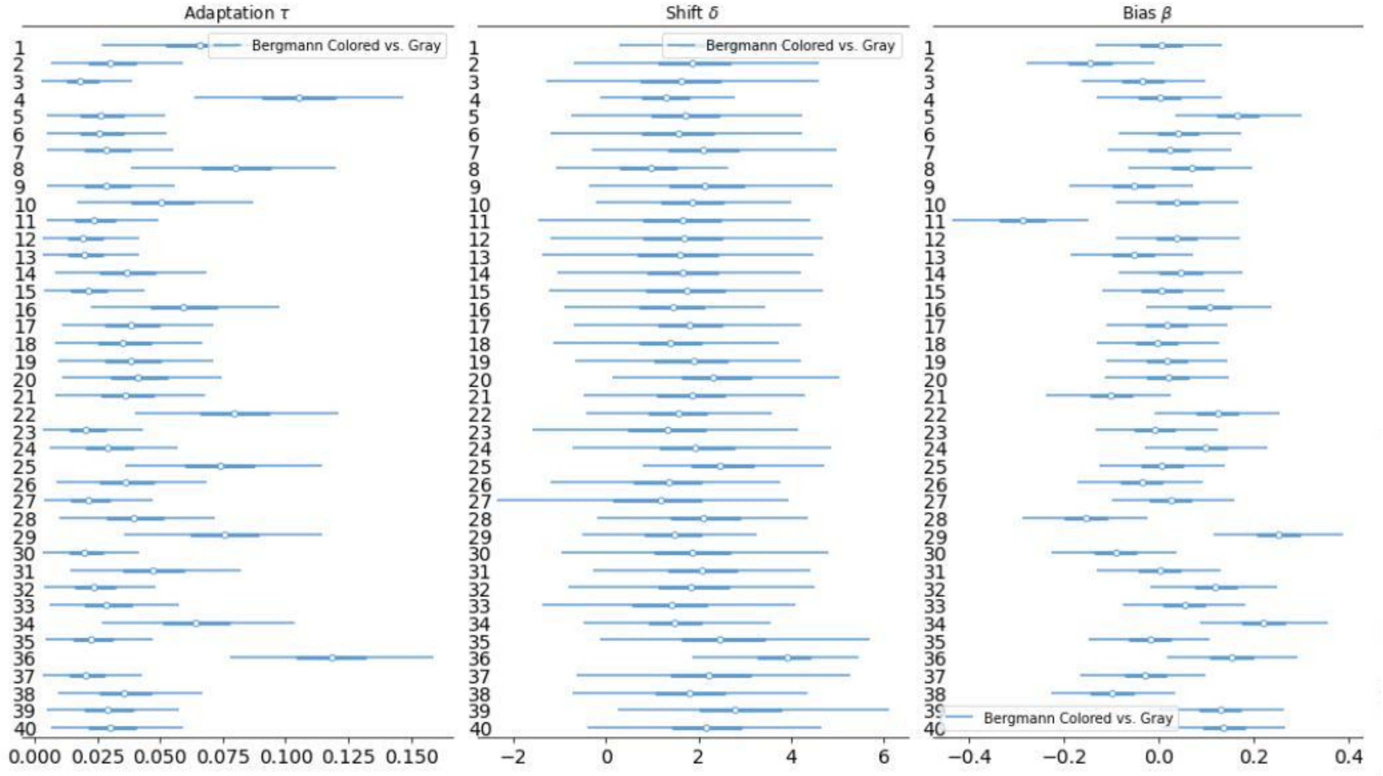

Figure S10C. *Posterior distributions of the participant-level estimates of adaptation τ , shift δ , and bias β for Bergmann et al.'s (2020) Experiment 1.*

In [27]:
```
1  acvsfit.plot_participant_posteriors(trace_bergmann_exp2, data_bergmann_exp2, colors=colors)
```

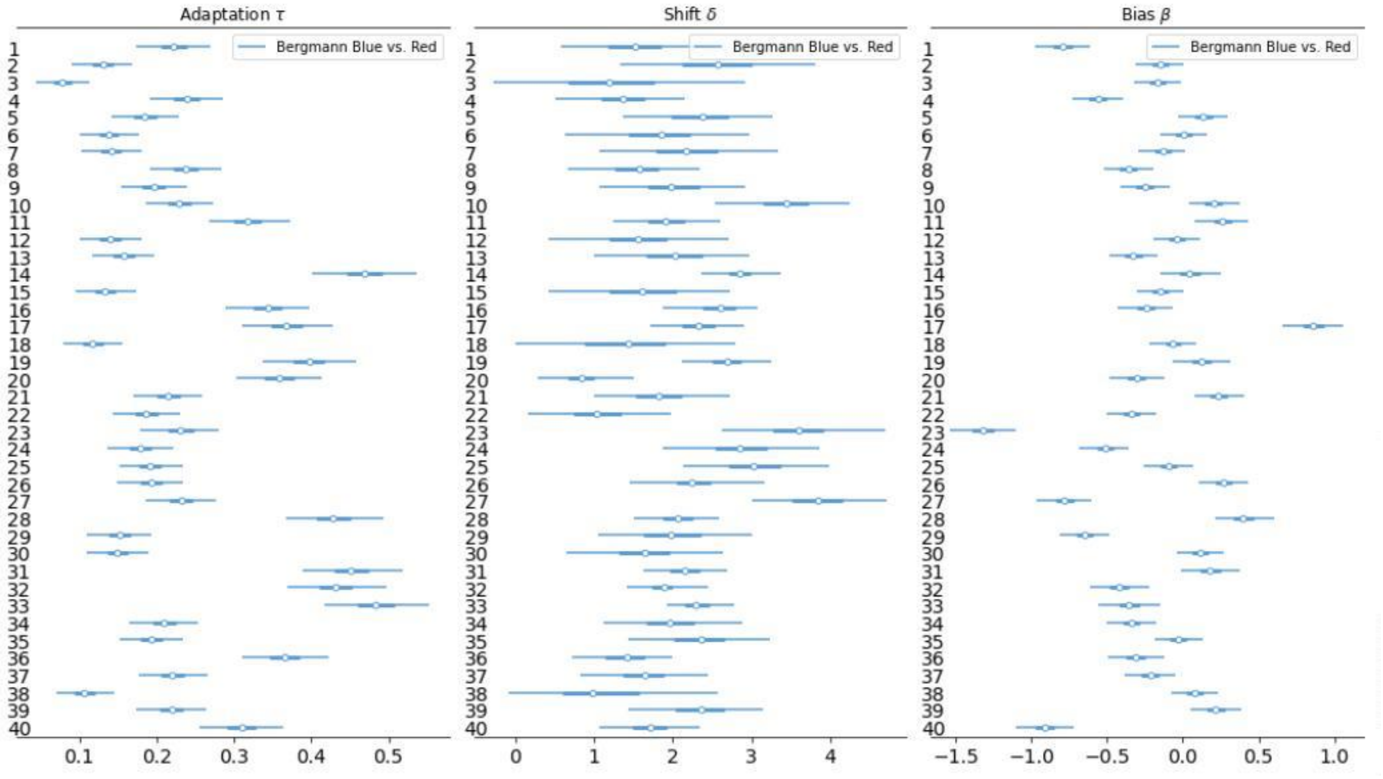

Figure S10D. *Posterior distributions of the participant-level estimates of adaptation τ , shift δ , and bias β for Experiment 2 ("blue vs. red").*

In [28]:

```
acvsfit.plot_priors_vs_posteriors(trace_exp1, colors['Online'][0],
                          'Experiment 1 (Online)', ax_lims={'bias_σ' : (0,2)})
acvsfit.plot_priors_vs_posteriors(trace_exp2, colors['Online'][0],
                          'Experiment 2 (Lab)', ax_lims={'bias_σ' : (0,2)})
acvsfit.plot_priors_vs_posteriors(trace_bergmann_exp1, colors['Bergmann Colored vs. Gray'][1],
                          "Bergmann et al.'s Experiment 1 (Colored vs Gray)", ax_lims={'bias_σ' : (0,2)})
acvsfit.plot_priors_vs_posteriors(trace_bergmann_exp2, colors['Bergmann Blue vs. Red'][0],
                          "Bergmann et al.'s Experiment 2 (Blue vs Red)", ax_lims={'bias_σ' : (0,2)})
```

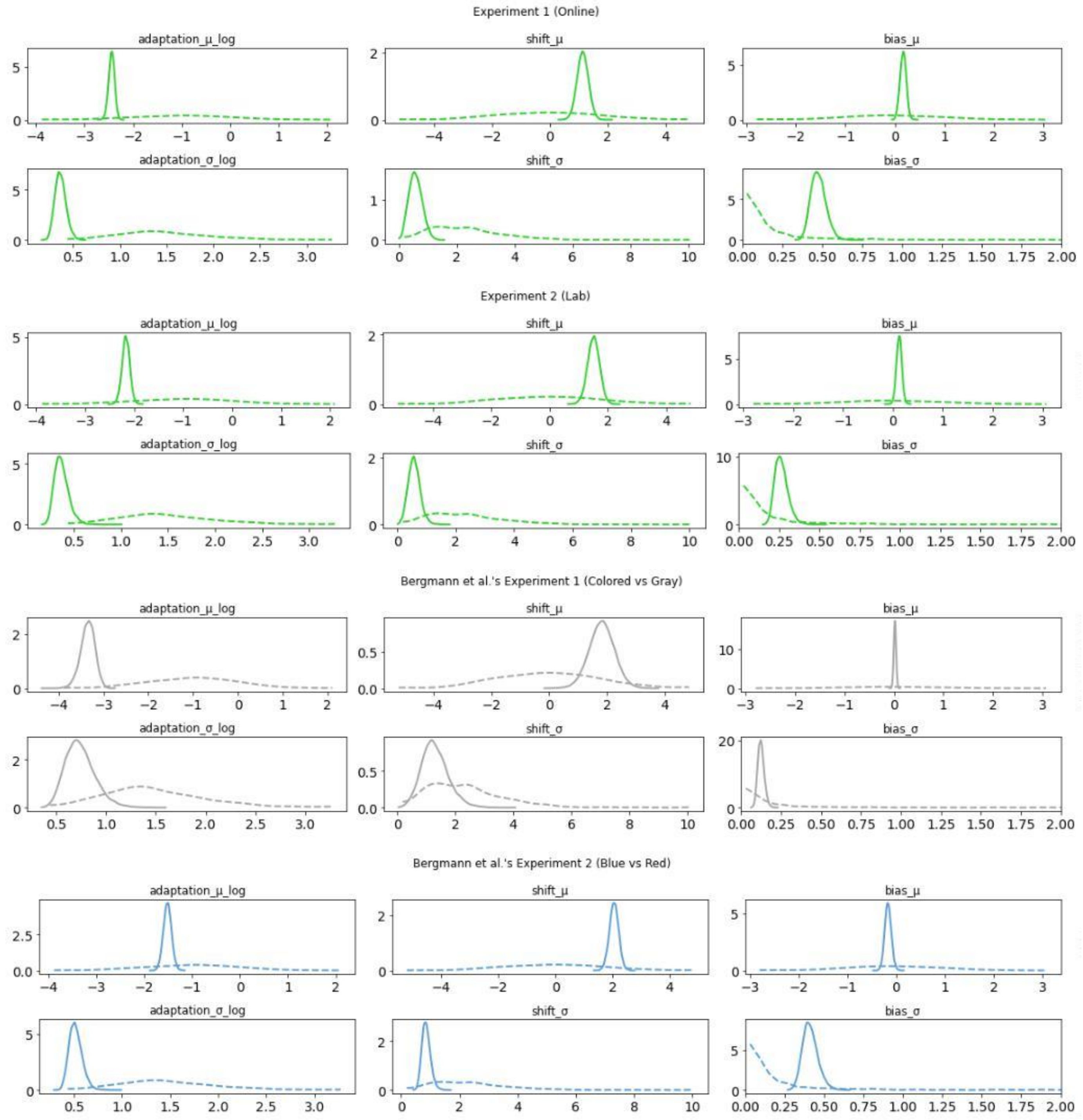

Figure S11. *Plots of the prior distributions (dashed lines) relative to the posterior distributions (solid lines) for Experiments 1 and 2 of the present study and Experiments 1 and 2's free choice data from Bergmann et al. (2020). As can be seen, the posteriors are much narrower than the priors and shifted, confirming that the data governed the posteriors.*

In addition to the visualizations in Figures S9 to S11, the last cell in Section 2.2C produces extensive tables with sampling statistics but also the mode and 95%-HDIs of every parameter in the model. They are saved as .csv-files to a subfolder called 'summaries'.

The cell below implements the calculation of differences between the experiments and produces a table with the estimates. It is encapsulated in a function so that it can be called in the upcoming sensitivity analysis to generate the quantities of interest.

In [29]:

```
def comparisons(texp1, texp2, tberg_exp1, tberg_exp2):
    datadict = {
        "Shift:E1_minus_E2": (texp1.posterior['shift_μ'].sel(Condition='Online')
                                           - texp2.posterior['shift_μ'].sel(Condition='Lab')),
        "Shift:E1_minus_BergE1": (texp1.posterior['shift_μ'].sel(Condition='Online')
                                                    - tberg_exp1.posterior['shift_μ']\
                                       .sel(Condition='Bergmann Colored vs. Gray')),
        "Shift:E1_minus_BergE2": (texp1.posterior['shift_μ'].sel(Condition='Online')
                                                     - tberg_exp2.posterior['shift_μ']\
                                       .sel(Condition='Bergmann Blue vs. Red')),
        "Shift:E2_minus_BergE1": (texp2.posterior['shift_μ'].sel(Condition='Lab')
                                                    - tberg_exp1.posterior['shift_μ']\
                                       .sel(Condition='Bergmann Colored vs. Gray')),
        "Shift:E2_minus_BergE2": (texp2.posterior['shift_μ'].sel(Condition='Lab')
                                                    - tberg_exp2.posterior['shift_μ']\
                                       .sel(Condition='Bergmann Blue vs. Red')),
        "Bias:E1_minus_E2": (texp1.posterior['bias_μ'] - texp2.posterior['bias_μ']),
        "Bias:E1_minus_BergE1": (texp1.posterior['bias_μ'] - tberg_exp1.posterior['bias_μ']),
        "Bias:E1_minus_BergE2": (texp1.posterior['bias_μ'] - tberg_exp2.posterior['bias_μ']),
        "Bias:E2_minus_BergE1": (texp2.posterior['bias_μ'] - tberg_exp1.posterior['bias_μ']),
        "Bias:E2_minus_BergE2": (texp2.posterior['bias_μ'] - tberg_exp2.posterior['bias_μ'])

    }
    if 'adaptation_μ_dim_0' in texp1.posterior:
        datadict.update({
          "Adapt.:E1_minus_E2": (texp1.posterior['adaptation_μ'] - texp2.posterior['adaptation_μ']),
          "Adapt.:E1_minus_BergE1": (texp1.posterior['adaptation_μ'] - tberg_exp1.posterior['adaptatio
          "Adapt.:E1_minus_BergE2": (texp1.posterior['adaptation_μ'] - tberg_exp2.posterior['adaptatio
          "Adapt.:E2_minus_BergE1": (texp2.posterior['adaptation_μ'] - tberg_exp1.posterior['adaptatio
          "Adapt.:E2_minus_BergE2": (texp2.posterior['adaptation_μ'] - tberg_exp2.posterior['adaptatio
        })
    else:
        datadict.update({
            "Adapt.:E1_minus_E2": (texp1.posterior['adaptation_μ'].sel(Condition='Online')
                                        - texp2.posterior['adaptation_μ'].sel(Condition='Lab')),
            "Adapt.:E1_minus_BergE1": (texp1.posterior['adaptation_μ'].sel(Condition='Online')
                                                    - tberg_exp1.posterior['adaptation_μ']\
                                                    .sel(Condition='Bergmann Colored vs. Gray')),
            "Adapt.:E1_minus_BergE2": (texp1.posterior['adaptation_μ'].sel(Condition='Online')
                                                    - tberg_exp2.posterior['adaptation_μ']\
                                                   .sel(Condition='Bergmann Blue vs. Red')),
            "Adapt.:E2_minus_BergE1": (texp2.posterior['adaptation_μ'].sel(Condition='Lab')
                                                    - tberg_exp1.posterior['adaptation_μ']\
                                                   .sel(Condition='Bergmann Colored vs. Gray')),
            "Adapt.:E2_minus_BergE2": (texp2.posterior['adaptation_μ'].sel(Condition='Lab')
                                                    - tberg_exp2.posterior['adaptation_μ']\
                                                   .sel(Condition='Bergmann Blue vs. Red'))
        })
    dataset = az.convert_to_inference_data(datadict)
    return dataset



dataset = comparisons(trace_exp1, trace_exp2, trace_bergmann_exp1, trace_bergmann_exp2)



def mode(values):
    return az.plots.plot_utils.calculate_point_estimate('mode', values)

az.summary(dataset, stat_funcs=[mode], extend=True)
```

Out[29]:

	mean	sd	hdi_2.5%	hdi_97.5%	mcse_mean	mcse_sd	ess_bulk	ess_tail	r_hat	mode
**Shift:E1_minus_E2**	-0.363	0.281	-0.923	0.177	0.003	0.002	10503.0	15463.0	1.00	-0.346
**Shift:E1_minus_BergE1**	-0.669	0.483	-1.608	0.290	0.004	0.003	15061.0	17319.0	1.00	-0.660
**Shift:E1_minus_BergE2**	-0.910	0.256	-1.431	-0.424	0.003	0.002	5628.0	10396.0	1.00	-0.896
**Shift:E2_minus_BergE1**	-0.305	0.485	-1.283	0.612	0.004	0.003	17453.0	18557.0	1.00	-0.344
**Shift:E2_minus_BergE2**	-0.546	0.262	-1.067	-0.030	0.003	0.002	7593.0	13041.0	1.00	-0.511
**Bias:E1_minus_E2**	0.043	0.082	-0.124	0.198	0.003	0.002	845.0	2648.0	1.00	0.040
**Bias:E1_minus_BergE1**	0.148	0.067	0.018	0.278	0.003	0.002	591.0	1600.0	1.01	0.149
**Bias:E1_minus_BergE2**	0.334	0.094	0.142	0.506	0.003	0.002	807.0	2248.0	1.01	0.340
**Bias:E2_minus_BergE1**	0.105	0.059	-0.014	0.219	0.001	0.001	6747.0	10430.0	1.00	0.102
**Bias:E2_minus_BergE2**	0.291	0.087	0.123	0.466	0.002	0.001	2189.0	4249.0	1.00	0.299
**Adapt.:E1_minus_E2[0]**	-0.030	0.012	-0.054	-0.007	0.000	0.000	6820.0	11031.0	1.00	-0.029
**Adapt.:E1_minus_BergE1[0]**	0.046	0.010	0.026	0.063	0.000	0.000	6635.0	10838.0	1.00	0.047
**Adapt.:E1_minus_BergE2[0]**	-0.161	0.025	-0.209	-0.115	0.001	0.000	1347.0	2688.0	1.00	-0.153
**Adapt.:E2_minus_BergE1[0]**	0.076	0.013	0.049	0.102	0.000	0.000	9905.0	12721.0	1.00	0.076
**Adapt.:E2_minus_BergE2[0]**	-0.130	0.026	-0.182	-0.081	0.001	0.000	1491.0	2870.0	1.00	-0.128

Table S2. *Comparisons between experiments. Descriptions of the posteriors (via mode and 95%-HDI boundaries) and diagnostic values (r_hat = Gelman-Rubin's R-Hat statistic. Indicates convergence if close to 1; ess = effective sample size, indicate how many samples are approximately available if one accounts for autocorrelation within the chains).*

2.4 Report of decisions (if any) and their criteria (BARG Step 4)

Our analysis does not include any point decisions. We set out to quantify the amount of adaptation, shift, and bias in the different experiments. As can be seen in Figure S9, the group-level estimates indicate substantial shift and adaptation clearly different from zero, with zero outside the 95 % HDI ranges. However, we conceive assessment of these posteriors not as all-or-nothing decisions but as a gradual assignment of the plausibility of different parameter values. Note that the group-level biases are estimated closer to zero, but as the participant-level estimates (see Figure S10) illustrate, there are substantial individual differences with some people going toward extreme biases toward one or the other shape (or color in the Bergmann experiments). This is not unexpected, as the bias might be driven by idiosyncratic preferences or strategies.

Table 2 above quantifies the differences between the experiments, again assigning plausibilities to how much the experiments differ in adaptation, shift, and bias (see discussion in the main text). Again, no point decisions are made at this stage.

2.5 Report of sensitivity analysis (BARG Step 5)

In the present research, we used priors that are default priors of the acvsfit framework. They are broad so that they can accommodate a lot of different experimental data and so that they are only slightly informed to discourage implausible ranges (see Section 2.1C). However, three questions concerning these priors arose after fitting the model, which are addressed in the sensitivity analysis.

1. The prior of shift_mu ~ Normal(0, σ=1.625), the hyperprior on the shift paramter, seems rather narrow with the standard deviation of 1.625 determined automatically by the acvsfit framework (based on the transition length), given that Bergmann et al.'s (2020) Experiment 2 data leads to shift_mu estimates of 2. To be sure that the shift_mu was not selected to restrictive, we refit the model with a substantially broader shift_mu ~ Normal(0, σ=3) prior.
2. Piloting of the acvsfit framework has shown that sometimes the shift estimates suffer from implosive shrinkage. That is, the shift_sigma piles up against zero, and the participant-level estimates all produce the same distributions. This is not necessarily a problem but since we are working with data from individuals whose psychological variables are highly likely to vary interindividually, the default prior implemented in acvsfig does take this into account using a Gamma distribution with alpah=3, shifting the prior slighly away from values at zero. In this sensitivity analysis, we explore what happens if we move the prior back towad values close to zeror. The rationale behind this is twofold: (1) It might help to further improve the acvsfit framework by checking whether implosive shrinkage occurs with the present data. (2) Implosive shrinkage could also result from the fact that observers in the experiments really have little variability in their shifts. Perhaps, the discrete nature of the trials leads to shifts close to integer values (for instance, observers could base their decisions on the nth-last trial; shifts would hten cluster around integer values). If there is such a theoretical reason for implosive shrinkage, it would be interesting to

know if and how allowing for it (or discouraging it, as with the current default prior) would change the outcomes or interpretations of the present study. To that end, we set the prior to Gamma($\alpha = 1$,$\beta = 3$) (equivalent to Exponential($\beta = 3$)), which consititutes a very narrow distribution peaking at zero.

3. The default prior on the adaptation parameter disables the negative range. As explained in Section 2.1C, the reason for this is that negative adaptation is highly unlikely. However, the fact the prior lies only in the positive range could produce a bias toward observing adaptation when there is none, at least when the data is weak. As can be seen in Section 2.3, especially in the Bergmann et al. (2020) Experiment 1 data, the adaptation estimate is small (yet shifted away from zero), and the adaptation curves rather flat (cf. Figure S7C and Figure 8, blue/gray plots). To verify that these small adaptation scores are not driven by the prior, we refit the model with a neutral adaptation_mu ~ Normal($\mu = 0$,$\sigma = 1$) and adaptation_sd ~ HalfCauchy($\alpha = 0.1$) as hyperprior, which leads to neutral priors on adaptation which do not impose any bias toward the

The cell below fits the different variants explained above and saves them to disk.

In [ ]:
```
sensitivity_analysis = [
    {
        'name': 'broad_shift_mu',
        'priors': {'shift_mu' : "pm.Normal('shift_μ', 0, 3, dims=['Condition'])"},
        'links': None
    },
    {
        'name': 'tighter_shift_sigma', # To address point 2 above
        'priors': {'shift_sigma': "pm.Gamma('shift_σ', alpha=1, beta=3, dims=['Condition'])"},
        'links': None
    },
    {
        'name': 'neutral_adaptation', # To address point 3 above
        'priors': {
            'adaptation_mu_log':
                "pm.Normal('adaptation_mu_neutral', mu=0, sigma=1, dims=['Condition'])",
            'adaptation_sigma_log':
                "pm.HalfCauchy('adaptation_sd_neutral', beta=0.1, dims=['Condition'])"
        },
        'links' : {'adaptation' : lambda x: x}
    },
]

datasets = [('exp1', phases, data_exp1), ('exp2', phases, data_exp2),
            ('exp1_bergmann', phases_bergmann_exp1, data_bergmann_exp1),
            ('exp2_bergmann', phases_bergmann_exp2, data_bergmann_exp2)]

for s in sensitivity_analysis:
    for d in datasets:
        print('Getting samples for ' + s['name'])
        model = acvsfit.get_model(d[1], d[2], custom_priors=s['priors'],
                                  custom_links=s['links'], silent=True)

        acvsfit.get_samples(model, samples=20000, thin=5,
                file='traces/sens_%s_%s.nc'%(s['name'],d[0]), seed=random_seed, silent=True, compress=
```

The cell below loads the traces from disk.

In [32]:

```python
default = [trace_exp1, trace_exp2, trace_bergmann_exp1, trace_bergmann_exp2]

neutral_adapt = [az.from_netcdf('traces/sens_neutral_adaptation_exp1.nc'),
                 az.from_netcdf('traces/sens_neutral_adaptation_exp2.nc'),
                 az.from_netcdf('traces/sens_neutral_adaptation_exp1_bergmann.nc'),
                 az.from_netcdf('traces/sens_neutral_adaptation_exp2_bergmann.nc')]

for trace in neutral_adapt:
    trace.rename({'adaptation_mu_neutral': 'adaptation_μ'}, inplace=True)
    trace.rename({'adaptation_sd_neutral': 'adaptation_σ'}, inplace=True)

broad_shift_mu = [az.from_netcdf('traces/sens_broad_shift_mu_exp1.nc'),
                  az.from_netcdf('traces/sens_broad_shift_mu_exp2.nc'),
                  az.from_netcdf('traces/sens_broad_shift_mu_exp1_bergmann.nc'),
                  az.from_netcdf('traces/sens_broad_shift_mu_exp2_bergmann.nc')]

tight_shift_sigma = [az.from_netcdf('traces/sens_tighter_shift_sigma_exp1.nc'),
                     az.from_netcdf('traces/sens_tighter_shift_sigma_exp2.nc'),
                     az.from_netcdf('traces/sens_tighter_shift_sigma_exp1_bergmann.nc'),
                     az.from_netcdf('traces/sens_tighter_shift_sigma_exp2_bergmann.nc')]
```

In [33]:

```python
f, ax = plt.subplots(6,4, figsize=(16,8), sharex='row')

traces = [default, broad_shift_mu, tight_shift_sigma, neutral_adapt]
var_names = ['adaptation_μ', 'adaptation_σ', 'shift_μ','shift_σ' ,'bias_μ' ,'bias_σ']

for t,trace in enumerate(traces):
    for v,var_name in enumerate(var_names):
        az.plot_forest(trace, var_names=[var_name], combined=True, ax=ax[v,t])
        ax[v,t].set_yticklabels([])
        ax[v,t].get_legend().remove()
        ax[v,t].set_title(None)
        ax[v,0].set_ylabel(var_name)

ax[0,0].set_title('Default')
ax[0,1].set_title('Broader shift_μ')
ax[0,2].set_title('Tighter shift_σ')
ax[0,3].set_title('Neutral adaptation_μ')

plt.tight_layout()
```

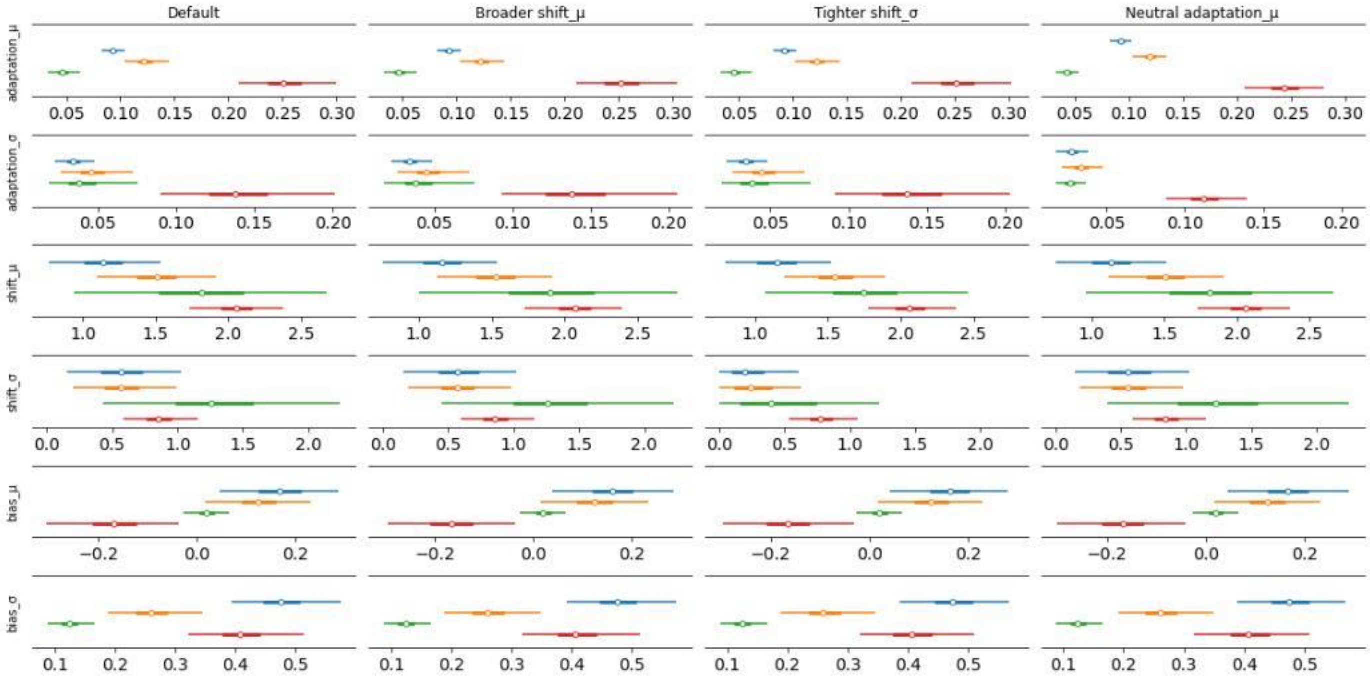

Figure S12. *Overview of the group-level estimates under the different priors of the sensitivity analysis. (colors: blue: default, orange: broader sigma_μ, green: tighter shift_σ, red: neutral adaptation.)*

The cell below performs all the comparisons of our original analysis using the traces oibtained with the different priors of the sensitivity analysis.

In [34]:

```
clist = [comparisons(*default), comparisons(*broad_shift_mu),
         comparisons(*tight_shift_sigma), comparisons(*neutral_adapt)]

f,ax = plt.subplots(3,1, figsize=(14, 20))
az.plot_forest(clist, combined=True, var_names=['Adapt'], filter_vars='like', ax=ax[0])
az.plot_forest(clist, combined=True, var_names=['Shift'], filter_vars='like', ax=ax[1])
az.plot_forest(clist, combined=True, var_names=['Bias'], filter_vars='like', ax=ax[2])

for c in range(3):
    ax[c].legend(['Neutral adapt.', 'Tight shift_σ', 'Broad shift_μ', 'Default/original'])

plt.tight_layout()
```

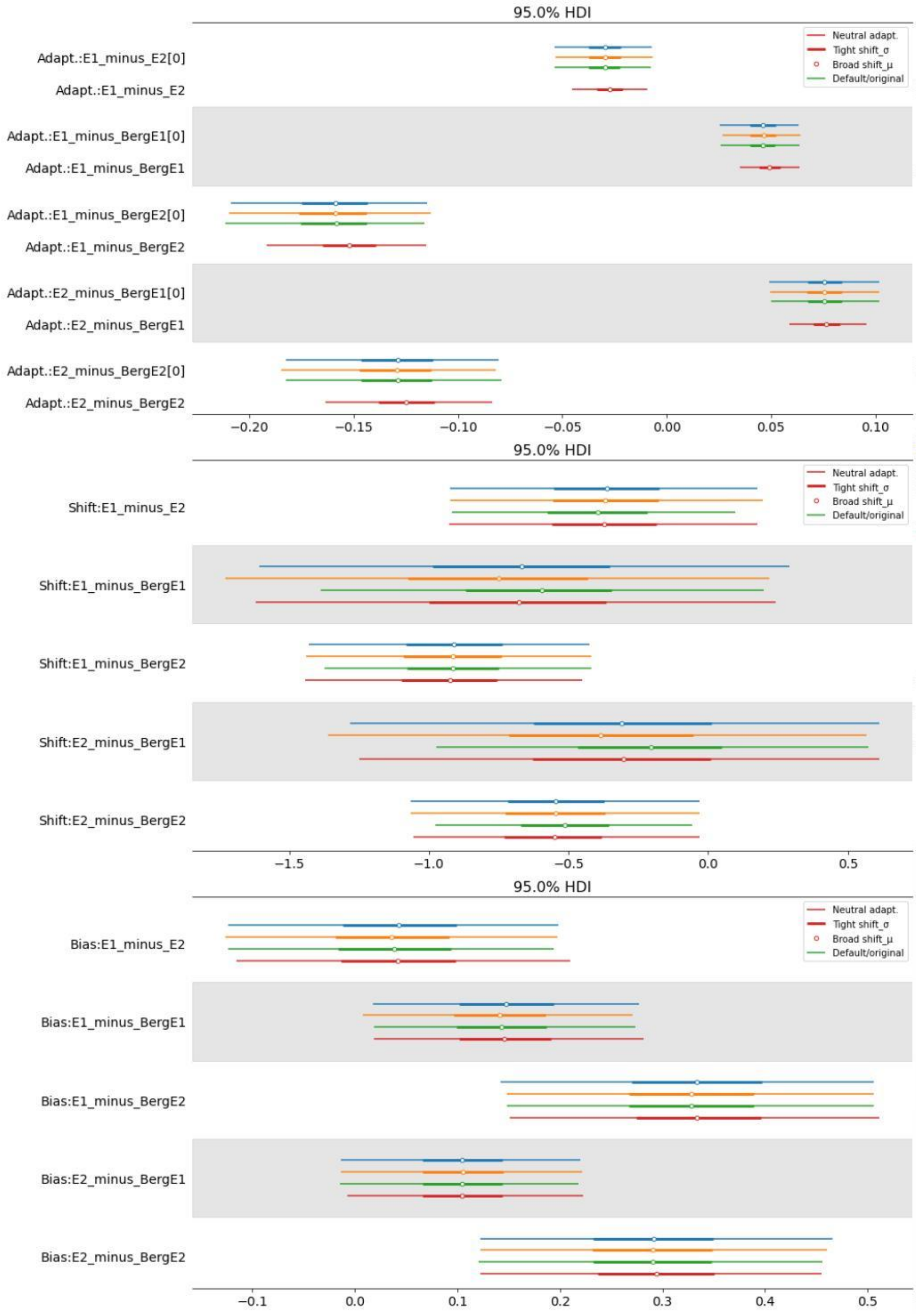

Figure S13. *Estimates of the differences between the experiments under the different priors of the sensitivity analysis.*

At first glance, none of the prior alternatives leads to substantial changes in the results. At a closer look, some observations can be made. Concerning the three points listed at the start of Section 2.5:

1. The broader prior on shift_μ does not change any of the estimates depicted in Figure S12, nor are the comparisons between experiments affected (Figure S13). Hence, the original prior was not too restrictive.
2. The tighter prior for shift_σ has moved the shift_σ estimates closer to zero, as can be seen in Figure S12 (row 4, column 3). Figure S14A and B are following up on this. As can be seen (using the data from Experiment 1), the shift_σ posterior is piled up against zero, and the participant-level shift estimates (plot bundle labeled "shift") show virtually the same distribution for each individual. Figure S14B shows the default prior, which we used in the original analysis. As can be seen there, shift_σ is not piled up against zero, and the participant-level shifts show some variability. The implosive shrinkage with the tight shift_σ occurs similarly in Experiment 2's data and Experiment 1 from Bergmann et al. (2020) but not for their Experiment 2 (which led to the strongest adaptation of all data sets we considered). Hence, this effect might occur when relatively low adaptation leads to strong uncertainty about the shift. As can be seen in Figures S12 and S13 (green lines), the other parameters and the estimated differences are not affected by using one or the other prior. These findings show that the original prior is helpful to maintain variability in the shift parameter between the individuals, but on the group level, either prior leads to the same results.
3. The hyperpriors that impose neutral (centered at zero) normal priors on the participant levels lead to slight differences in the estimates of the adaptation_μ and adaptation_σ parameters compared to the other priors (cf. Figure S12 (rightmost column) and in the estimated differences between experiments (Figure S13, red lines). In Figure S14C, it can be seen that even with data that only shows weak adaptation (Bergmann et al.'s Experiment 1), the distribution of adaptation_μ does not reach into the negative range, and the modes of the participant-level "adaptation" parameters are positive as well (only a small part of the distributions lies in the negative range, indicating that for them there remains some plausibility of small negative adaptation values). For all other experiments, the participant-level distributions are entirely positive (not shown in the figure). With respect to concern (3) explained in Section 2.5, it is not the case that the original prior, which only resides in the positive range, spuriously drives the adaptation scores when adaptation is weak.

In [35]:

```
def mode(values):
    return az.plots.plot_utils.calculate_point_estimate('mode', values)
az.summary(trace_exp1,var_names=['adaptation', 'shift', 'bias'], stat_funcs=[mode], extend=True)\
                                    .to_csv('summaries/summary_exp1.csv')
az.summary(trace_exp2,var_names=['adaptation', 'shift', 'bias'], stat_funcs=[mode], extend=True)\
                                    .to_csv('summaries/summary_exp2.csv')
```

In [42]:

```python
#pd.set_option('display.max_rows', 100)
summary_df = pd.read_csv('summaries/summary_exp1.csv')
display(summary_df)
print('Participant 2 had weak adaptation')
print(summary_df.iloc[[1]])
print('Participant 28 had strong adaptation')
print(summary_df.iloc[[22]])
```

	Unnamed: 0	mean	sd	hdi_2.5%	hdi_97.5%	mcse_mean	mcse_sd	ess_bulk	ess_tail	r_hat	mode
**0**	adaptation[0, 0]	0.089	0.020	0.051	0.129	0.000	0.000	10001.0	11266.0	1.0	0.087
**1**	adaptation[1, 0]	0.079	0.019	0.043	0.117	0.000	0.000	8367.0	11923.0	1.0	0.074
**2**	adaptation[2, 0]	0.098	0.023	0.054	0.144	0.000	0.000	10528.0	13804.0	1.0	0.094
**3**	adaptation[3, 0]	0.104	0.023	0.061	0.149	0.000	0.000	8621.0	11685.0	1.0	0.100
**4**	adaptation[4, 0]	0.095	0.021	0.055	0.137	0.000	0.000	6265.0	8824.0	1.0	0.092
**...**	...	...	...	...	...	...	...	...	...	...	...
**190**	bias[60]	-0.075	0.102	-0.284	0.116	0.001	0.001	10919.0	14535.0	1.0	-0.051
**191**	bias[61]	-0.230	0.101	-0.422	-0.027	0.001	0.001	10709.0	13620.0	1.0	-0.245
**192**	bias[62]	2.350	0.174	2.020	2.700	0.002	0.001	12169.0	16137.0	1.0	2.331
**193**	bias[63]	0.109	0.101	-0.085	0.311	0.001	0.001	9928.0	14571.0	1.0	0.108
**194**	bias[64]	0.113	0.100	-0.090	0.305	0.001	0.001	11443.0	15514.0	1.0	0.118

195 rows × 11 columns

```
Participant 2 had weak adaptation
        Unnamed: 0   mean     sd  hdi_2.5%  hdi_97.5%  mcse_mean  mcse_sd  \
1  adaptation[1, 0]  0.079  0.019     0.043      0.117        0.0      0.0

   ess_bulk  ess_tail  r_hat   mode
1    8367.0   11923.0    1.0  0.074
Participant 28 had strong adaptation
         Unnamed: 0   mean     sd  hdi_2.5%  hdi_97.5%  mcse_mean  mcse_sd  \
22  adaptation[22, 0]  0.157  0.028     0.101      0.211        0.0      0.0

    ess_bulk  ess_tail  r_hat   mode
22   10675.0   12184.0    1.0  0.157
```

In [37]:

```python
#pd.set_option('display.max_rows', 100)
summary_df = pd.read_csv('summaries/summary_exp2.csv')
#display(summary_df)
print('Participant 1 had strong adaptation')
print(summary_df.iloc[[0]])
print('Participant 25 had weak adaptation')
print(summary_df.iloc[[23]])
```

```
Participant 1 had strong adaptation
        Unnamed: 0   mean    sd  hdi_2.5%  hdi_97.5%  mcse_mean  mcse_sd  \
0  adaptation[0, 0]  0.191  0.02     0.152      0.231        0.0      0.0

   ess_bulk  ess_tail  r_hat   mode
0   18246.0   18933.0    1.0  0.189
Participant 25 had weak adaptation
         Unnamed: 0   mean     sd  hdi_2.5%  hdi_97.5%  mcse_mean  mcse_sd  \
23  adaptation[23, 0]  0.097  0.016     0.066      0.129        0.0      0.0

    ess_bulk  ess_tail  r_hat   mode
23   18767.0   18316.0    1.0  0.096
```

In [38]:
```
az.plot_trace(tight_shift_sigma[0], var_names=['shift'], filter_vars='like')
plt.suptitle('A: With tighter prior on shift_σ (Experiment 1 data)')
```

Out[38]:
```
Text(0.5, 0.98, 'A: With tighter prior on shift_σ (Experiment 1 data)')
```

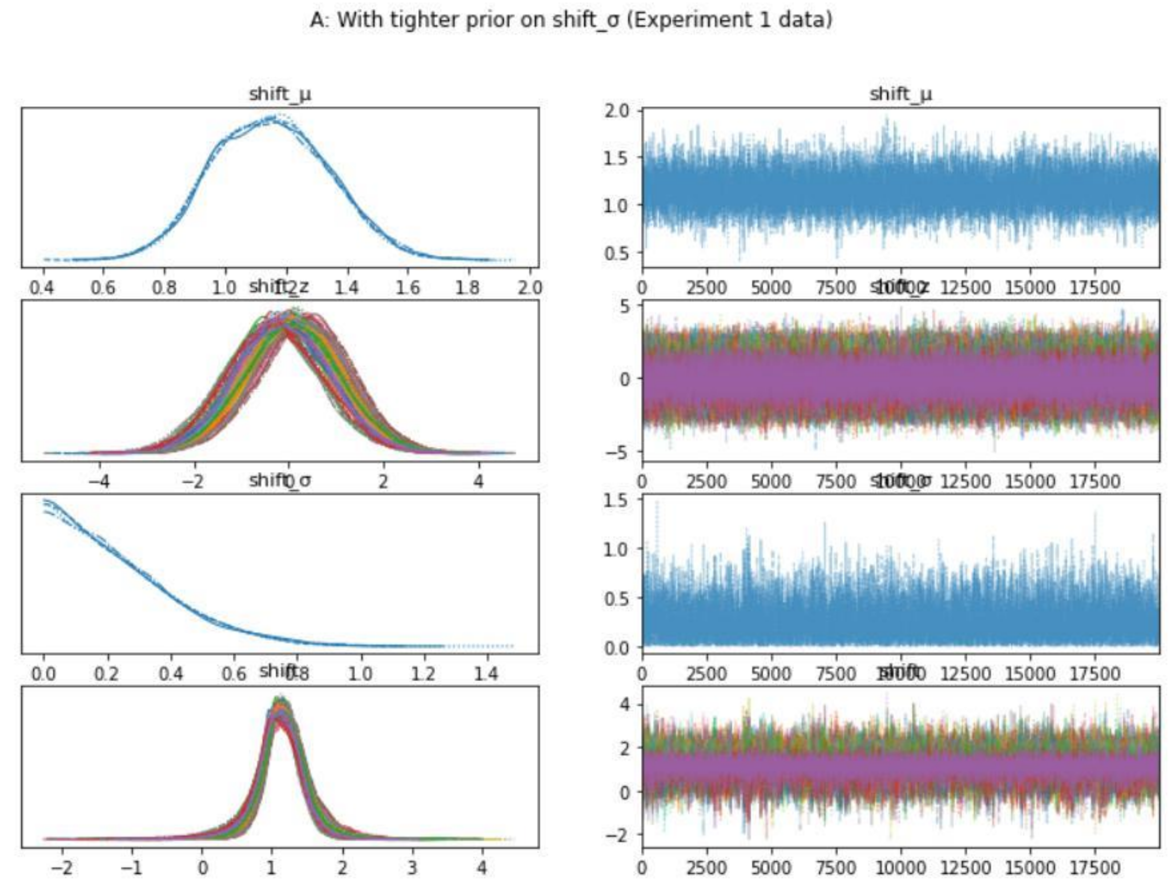

Figure S14A. *Trace plots of the shift parameters when using the tight prior on shift_σ .*

In [39]:
```
az.plot_trace(default[0], var_names=['shift'], filter_vars='like')
plt.suptitle('B: With with Gamma-prior that discourages zero σ (Experiment 1 data)')
```

Out[39]:
```
Text(0.5, 0.98, 'B: With with Gamma-prior that discourages zero σ (Experiment 1 data)')
```

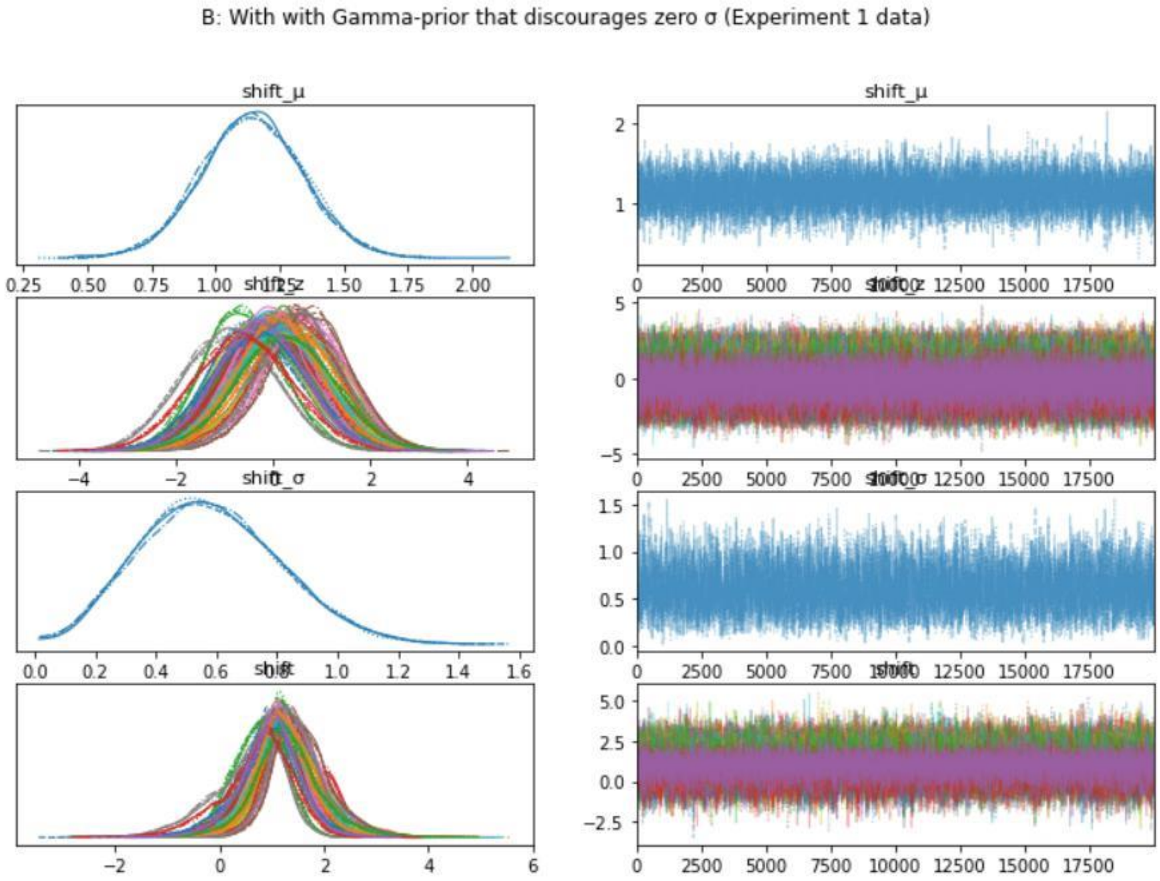

Figure S14B. *Trace plots of the shift parameters when using the Gamma-prior that discourages very small values close to zero.*

In [40]:
```
az.plot_trace(neutral_adapt[1], var_names=['adaptation'], filter_vars='like')
plt.suptitle("C: With neutral adaptaion prior (Bergmann et al's Experiment 2 data)")
```

Out[40]:
```
Text(0.5, 0.98, "C: With neutral adaptaion prior (Bergmann et al's Experiment 2 data)")
```

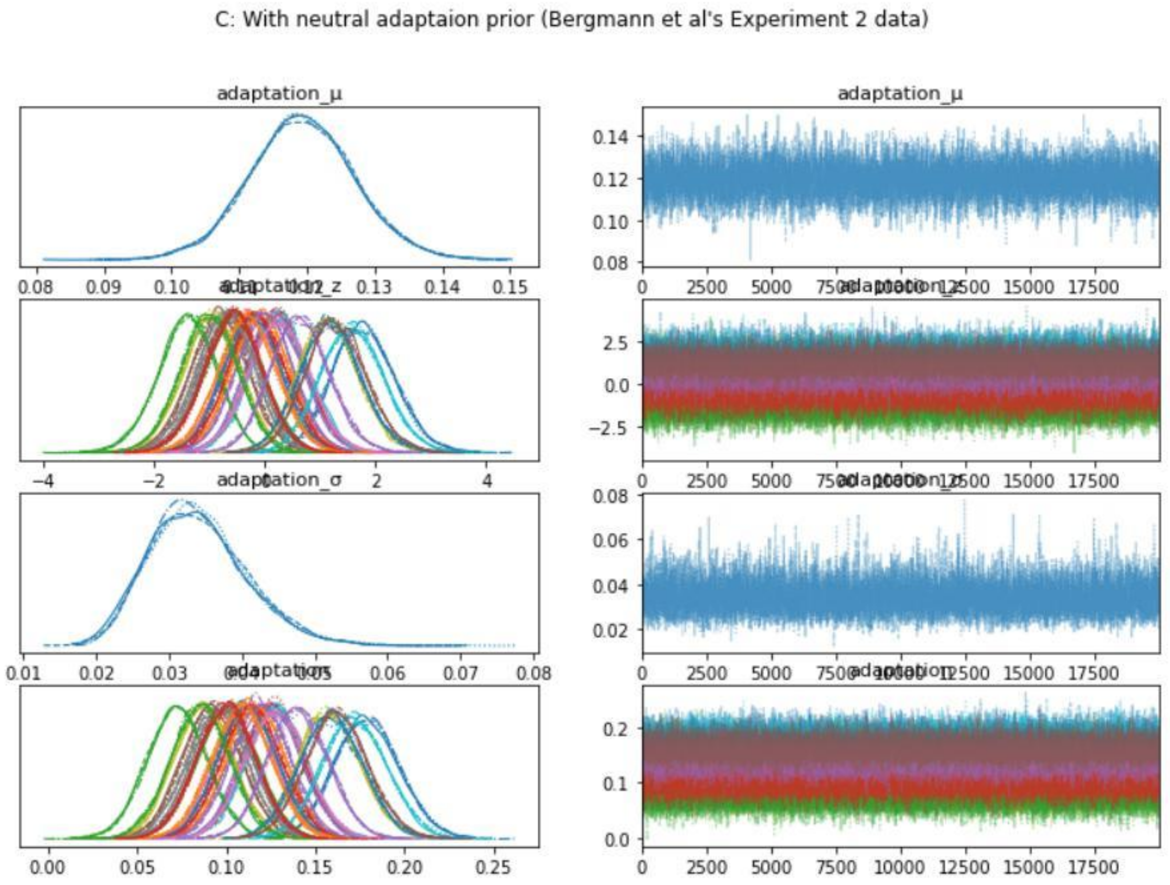

Figure S14C. *Plots shows distributions and traces obtained with the neutral adaptation prior.*

2.6 Reproducibility (BARG Step 6)

A. Software and installation. Running this analysis requires a Python 3 environment with the *acvsfit* library; to perform the analysis in the interactive notebook, a **Juypter/IPython** (https://jupyter.org/) setup ist required . The acvsfit framework is built on top of the probabilistic programming and visualization libraries *PyMC* and *ArviZ*. In addition, the *seaborn* plotting library is used. Installing the acvsfit package via the Python package installer pip (pypi.org) should pull all dependencies.

B. Software version details. The following version numbers were originally used. It is suggested to use these versions to exactly reproduce the results. However, other versions might still work. If different versions are used, the results should be checked against the original version of this notebook.

- acvsfit: 0.1.0
- PyMC: 4.0.0
- ArviZ: 0.12.1
- Seaborn: 0.12.0
- IPython: 7.23.1

You can remove the '#' in the cell below and execute it to install acvsfit and its dependencies.

In [41]:
```
# !pip install git+https://github.com/jt-lab/acvsfit.git@v0.1.0
```

C. Script and data. The Jupyter notebook you are looking at contains the whole analysis procedure. The datasets are stored in the subfolder 'data' relative to this file. In addition, the 'phases' folder is required, which contains .json-files that specify the phases of the experiment cycles.

F. "Runs as posted". The notebook and data files were downloaded to different PCs to confirm that the analysis runs through as posted.

G. MCMC chains for time-intensive runs. The subfolder 'traces' contains traces with the MCMC chains that are produced by this analysis. If such a subfolder with traces exists (i.e., if you download the notebook and the folder with the preexisting traces), the `get_samples` function used in several cells will load the traces instead of resampling the model. The traces can also be loaded manually using the ArviZ function `from_netcdf(filename)` . They then can be inspected using ArviZ.

H. Reproducible MCMC. The analysis shown in this notebook was performed using a fixed seed for the random number generators involved (`random_seed = 1234`). As long as this seed is maintained, the notebook will produce the exact same output. Note that at some places it might be of interest to see plots created with different seeds. For instance, when looking at prior predictive simulations, seeing different random samples might help to better understand the variability contained in the prior. The seed can then be manually changed by passing another interger (or `None` , for random seeds) to the `seed` argument of the acvsfit functions.

References

Bergmann, N., Tünnermann, J., & Schubö, A. (2020). Which search are you on? Adapting to color while searching for shape. *Attention, Perception, & Psychophysics, 82*(2), 457–477. https://doi.org/10.1016/j.tics.2012.06.010 (https://doi.org/10.1016/j.tics.2012.06.010)

Harris, C. R., Millman, K. J., Van Der Walt, S. J., Gommers, R., Virtanen, P., Cournapeau, D., ... & Oliphant, T. E. (2020). Array programming with NumPy. *Nature, 585*(7825), 357–362. https://doi.org/10.1038/s41586-020-2649-2 (https://doi.org/10.1038/s41586-020-2649-2)

Hoffman, M. D., & Gelman, A. (2014). The No-U-Turn sampler: adaptively setting path lengths in Hamiltonian Monte Carlo. *Journal of Machine Learning Research, 15*(1), 1593–1623. https://www.jmlr.org/papers/volume15/hoffman14a/hoffman14a.pdf (https://www.jmlr.org/papers/volume15/hoffman14a/hoffman14a.pdf)

Irons, J. L., & Leber, A. B. (2016). Choosing attentional control settings in a dynamically changing environment. *Attention, Perception, & Psychophysics, 78*(7), 2031–2048. https://doi.org/10.3758/s13414-016-1125-4 (https://doi.org/10.3758/s13414-016-1125-4)

Irons, J. L., & Leber, A. B. (2018). Characterizing individual variation in the strategic use of attentional control. *Journal of Experimental Psychology: Human Perception and Performance, 44*(10), 1637–1654. https://doi.org/10.1037/xhp0000560 (https://doi.org/10.1037/xhp0000560)

Lee, M. D., & Wagenmakers, E. J. (2014). Bayesian cognitive modeling: A practical course. Cambridge University Press.

Kumar, R., Carroll, C., Hartikainen, A., & Martín, O. A. (2019). ArviZ a unified library for exploratory analysis of Bayesian models in Python. *Journal of Open Source Software, 4*(33). https://joss.theoj.org/papers/10.21105/joss.01143 (https://joss.theoj.org/papers/10.21105/joss.01143)

Kruschke, J. K. (2021). Bayesian analysis reporting guidelines. *Nature Human Behaviour, 5*(10), 1282–1291. https://www.nature.com/articles/s41562-021-01177-7 (https://www.nature.com/articles/s41562-021-01177-7)

McElreath, R. (2020). Statistical rethinking: A Bayesian course with examples in R and Stan. Chapman and Hall/CRC. https://doi.org/10.1201/9780429029608 (https://doi.org/10.1201/9780429029608)

McKinney, W. (2010, June). Data structures for statistical computing in python. In *Proceedings of the 9th Python in Science Conference (Vol. 445, No. 1, pp. 51–56)*. https://conference.scipy.org/proceedings/scipy2010/pdfs/mckinney.pdf (https://conference.scipy.org/proceedings/scipy2010/pdfs/mckinney.pdf)

Liddell, T. M., & Kruschke, J. K. (2018). Analyzing ordinal data with metric models: What could possibly go wrong?. *Journal of Experimental Social Psychology, 79*, 328–348.

Salvatier, J., Wiecki, T. V., & Fonnesbeck, C. (2016). Probabilistic programming in Python using PyMC3. *PeerJ Computer Science, 2*, e55. https://peerj.com/articles/cs-55.pdf (https://peerj.com/articles/cs-55.pdf)

Waskom, M. L. (2021). Seaborn: statistical data visualization. *Journal of Open Source Software, 6*(60), 3021. https://doi.org/10.21105/joss.03021 (https://doi.org/10.21105/joss.03021)

Hunter, John D. (2007), Matplotlib: A 2D graphics environment. *Computing in Science & Engineering 9.03* 90–95. https://doi.ieeecomputersociety.org/10.1109/MCSE.2007.55 (https://doi.ieeecomputersociety.org/10.1109/MCSE.2007.55)

Supplement B: Adaptation over the time

To quantify how the adaptative behavior changed over the time, we fitted the data in the first and the last blocks on the participant level (and averaged the differences over all participants). The same procedure was applied to the data of Experiment 1 and 2 separately. The estimated adaptation τ, shift δ and bias β (see Table S3) reflected that participants adapted to the shape proportion change of distractors in the first blocks as well as in the last block in both experiments (Figure S15; S16).

Table S3

Estimated adaptation τ, shift δ and bias β in the first and last blocks in both experiments

	**Adaptation τ**	**Shift δ**	**Bias β**
Exp. 1 Block 1	0.082 [0.064, 0.1]	0.076 trials [-0.82, 0.97]	0.17 [0.0015, 0.3]
Exp. 1 Block 4	0.1 [0.084, 0.13]	0.86 trials [0.18, 1.6]	0.12 [-0.0071, 0.25]
Exp. 2 Block 1	0.11 [0.078, 0.15]	1.8 trials [0.64, 3]	0.13 [-0.0044, 0.28]
Exp. 2 Block 8	0.13 [0.097, 0.17]	1.4 trials [0.42, 2.3]	0.1 [-0.025, 0.22]

Note. Modes and 95% HDIs of adaptation τ, shift δ and bias β estimates in the first and last blocks in Exp.1 and Exp.2.

As Figure S15 and Figure S16 show, in both experiments estimates of adaptation were stronger in the last block compared to the first. The differences are 0.021 [-0.0042, 0.05] for Exp. 1 and 0.016 [-0.039, 0.072] for Exp. 2, with zero (“no-difference”) within the 95% HDIs, indicating some uncertainty in this estimate. However, 95.1% (Exp. 1) and 74.5% (Exp. 2) of the probability density are in the positive range, that is, in favor of a higher adaptation in the last block.

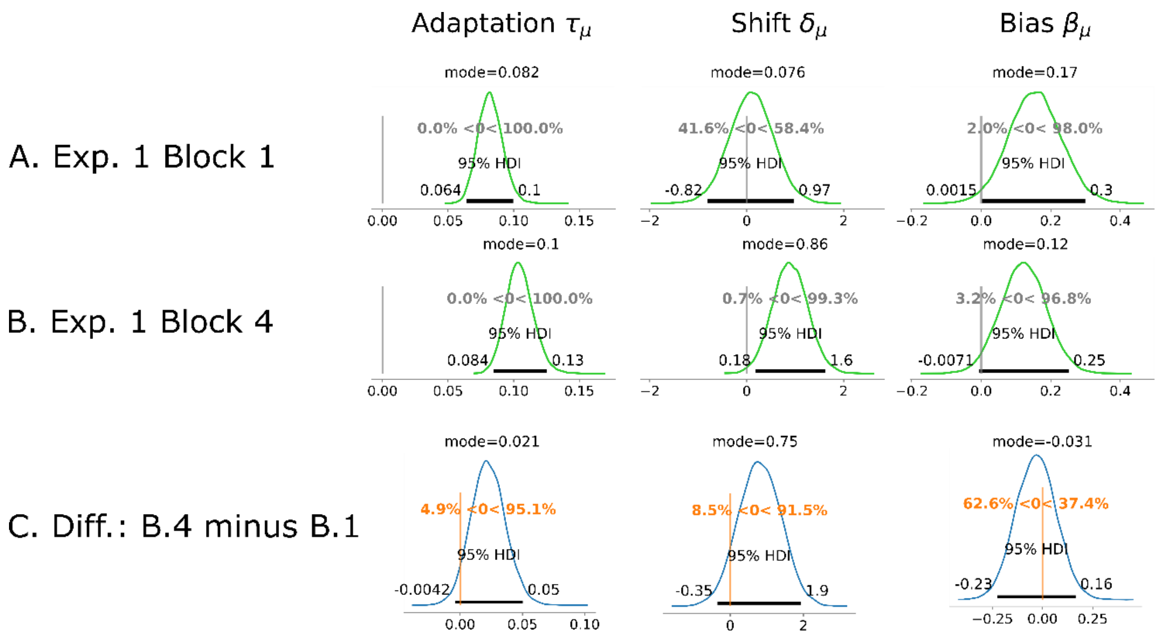

Figure S15. Group-level posterior distributions of adaptation τ, shift δ, and bias β estimates in **A.** block one, **B.** block four, and **C.** the difference of parameter estimates between two blocks in Experiment 1. The horizontal black bars and the numbers on their ends indicate the boundaries of 95% highest-density interval (HDI). The grey percentages compared to zero represent the proportion of posterior distributions that were smaller (left) and larger (right) than zero.

The model did not find much evidence for Shift δ in block one of Exp. 1. One possible reason could be that adaptation was low in the beginning of the experiment. Therefore, with low adaptation, the shift can also not be estimated with great certainty. For instance, if adaptation is zero, shift would be meaningless as an extreme case.

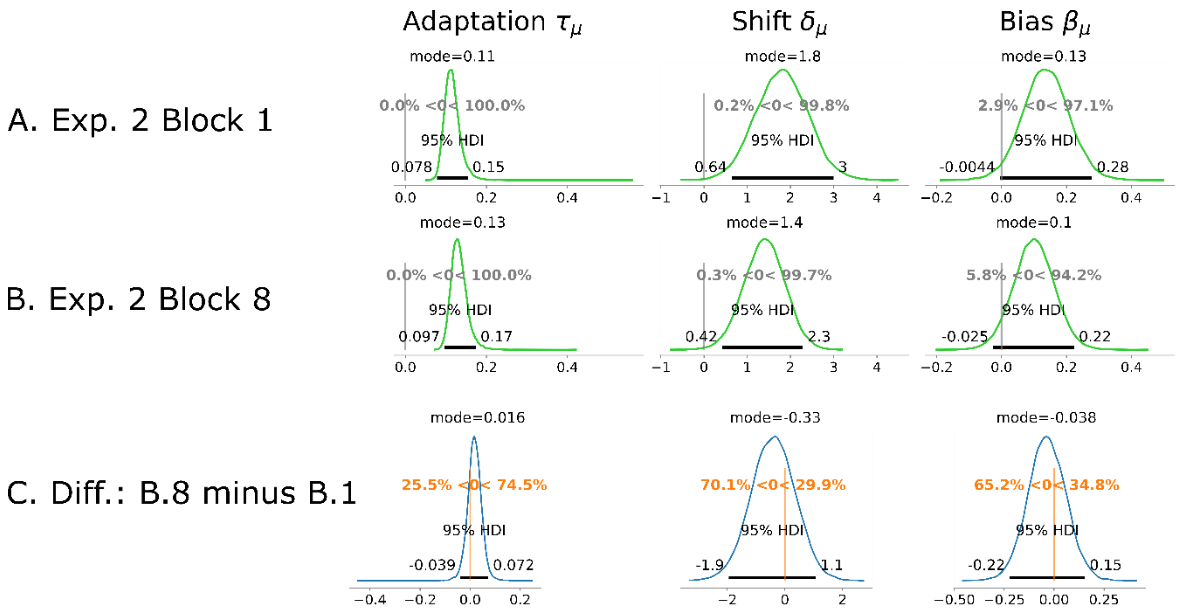

Figure S16. Group-level posterior distributions of adaptation τ, shift δ, and bias β estimates in **A.** block one, **B.** block eight, and **C.** the difference of parameter estimates between two blocks in Experiment 2. Other details were the same as in Figure S15.

Supplement C: Color chromaticity

Note that chromaticity *xy* (see Table S4) was not measured with a scientific spectrometer but with a display colorimeter typically used in display calibration (X-Rite i1Display Pro colorimeter; X-Rite GmbH, Germany). Luminance was measured with a luminance meter (LS-100, Konica Minolta Inc., Japan).

Table S4

Color details of two hues and the background used in the visual search task

Color	RGB	*xy*	Luminance
Blue	29, 73, 153	0.217, 0.221	29.89 cd/m^2
Black	0, 0, 0	0.240, 0.237	0.252 cd/m^2
Gray	191, 191, 191	0.298, 0.313	78.56 cd/m^2

Note. RGB, *xy* values and the luminance of blue, black and the background gray.

Supplement D: First fixations to blue and black items

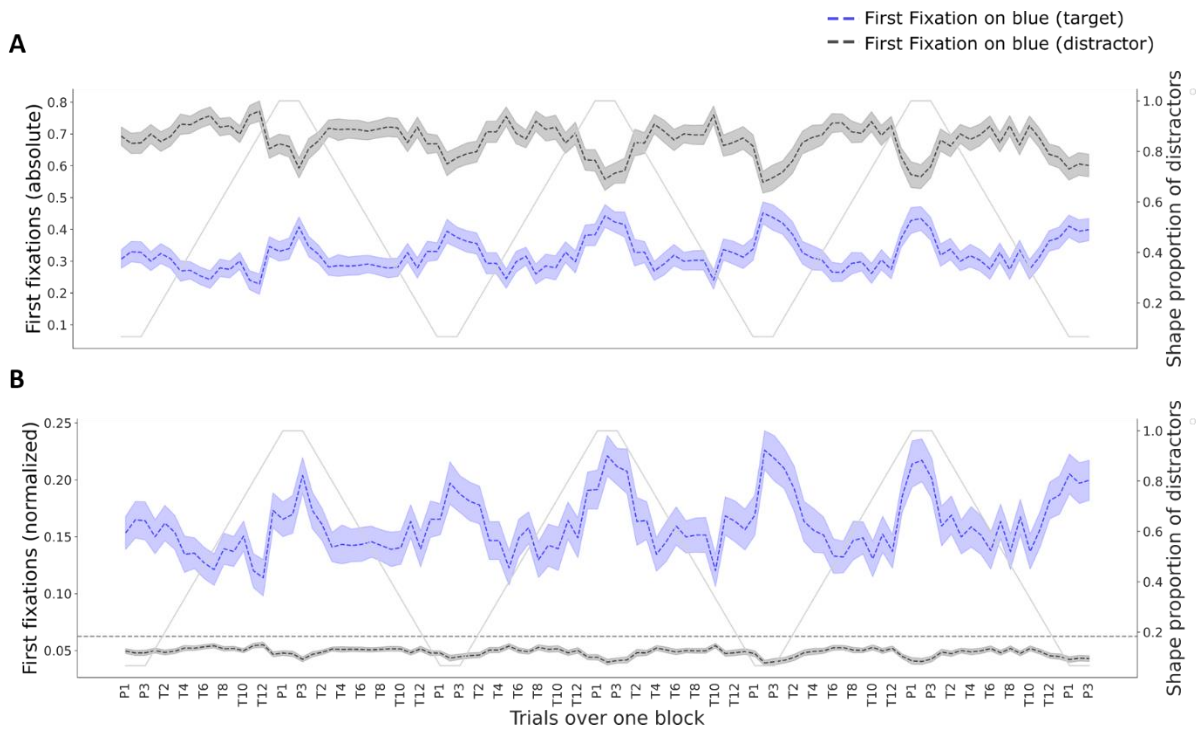

Figure S17. Fixation data from Experiment 2. **A**. Absolute proportions and **B.** normalized proportions of first fixations on blue targets and black distractors, averaged across trials in one block. The shaded area represents the standard errors of the means. The gray dotted horizontal line (1/16) represents the chance of fixating a random item. The solid gray lines represent the proportion of the two distractor shapes.
